# Fluorescent Biaryl Uracils with C5-Dihydro- and Quinazolinone Heterocyclic Appendages in PNA

**DOI:** 10.3390/molecules25081995

**Published:** 2020-04-24

**Authors:** Ali Heidari, Arash Ghorbani-Choghamarani, Maryam Hajjami, Robert H. E. Hudson

**Affiliations:** 1 Department of Chemistry, The University of Western Ontario, London, ON N6A 5B7, Canada; aheidar2@uwo.ca; 2 Department of Chemistry, Faculty of Science, Ilam University, Ilam 69315516, Iran; mhajjami@yahoo.com

**Keywords:** peptide nucleic acid, nucleobase-modified PNA, solid-phase synthesis, dihydroquinazolinone, quinazolinone, quinazolinone based nucleobase, fluorescent probes

## Abstract

There has been much effort to exploit fluorescence techniques in the detection of nucleic acids. Canonical nucleic acids are essentially nonfluorescent; however, the modification of the nucleobase has proved to be a fruitful way to engender fluorescence. Much of the chemistry used to prepare modified nucleobases relies on expensive transition metal catalysts. In this work, we describe the synthesis of biaryl quinazolinone-uracil nucleobase analogs prepared by the condensation of anthranilamide derivatives and 5-formyluracil using inexpensive copper salts. A selection of modified nucleobases were prepared, and the effect of methoxy- or nitro- group substitution on the photophysical properties was examined. Both the dihydroquinazolinone and quinazolinone modified uracils have much larger molar absorptivity (~4–8×) than natural uracil and produce modest blue fluorescence. The quinazolinone-modified uracils display higher quantum yields than the corresponding dihydroquinazolinones and also show temperature and viscosity dependent emission consistent with molecular rotor behavior. Peptide nucleic acid (PNA) monomers possessing quinazolinone modified uracils were prepared and incorporated into oligomers. In the sequence context examined, the nitro-substituted, methoxy-substituted and unmodified quinazolinone inserts resulted in a stabilization (∆T_m_ = +4.0/insert; +2.0/insert; +1.0/insert, respectively) relative to control PNA sequence upon hybridization to complementary DNA. All three derivatives responded to hybridization by the “turn-on” of fluorescence intensity by ca. 3-to-4 fold and may find use as probes for complementary DNA sequences.

## 1. Introduction

**Figure 1:**
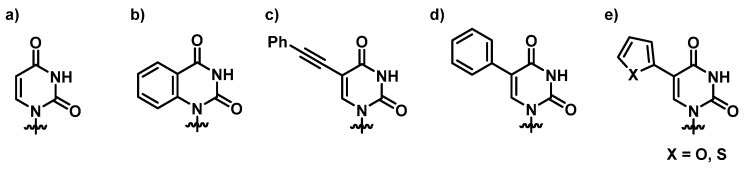
Selected examples of uracil nucleobase (**a**) structural modifications: ring fusion (**b**), extension of conjugation (**c**), biaryl (**d**) and heterocycle biaryl (**e**) derivatives. Ph = phenyl.

Since the 1980s, much effort has been devoted to exploiting fluorescence techniques in the detection and study of nucleic acids. While aromatic-based amino acids (phenylalanine, tyrosine and tryptophan) possess intrinsic fluorescence, natural nucleic acids are essentially nonfluorescent [1,2]. In order to facilitate studies with nucleic acids, structurally modified oligonucleotides that incorporate a luminophore have been pursued aggressively. There is a wide range of methods used to produce fluorescent oligonucleotides, for example, by the substitution of a structural feature—such as the nucleobase—with a fluorophore, or by appending a chromophore to the nucleobase or sugar-phosphate backbone [3]. An especially fruitful approach has been to expand the nucleobase by ring fusion or to extend the conjugation through an ethylene or ethyne bridge and/or by direct coupling to another aromatic moiety via a biaryl bond [4,5]; these routes overwhelmingly employ precious metal catalysts. The 5-heteroaromatic uracils are attractive targets as modified nucleobases since they have been shown to retain their hydrogen bonding ability to the complementary nucleobases but have the potential to be fluorescent and possess molecular rotor properties that enable them to be “turn on” probes of nucleic acid structure [6,7] (Figure 1).

**Figure 2:**
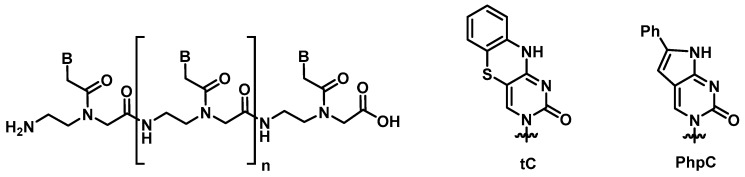
Generic structure of a peptide nucleic acid oligomer (PNA, left) and two examples of fluorescent nucleobases. B = nucleobase, Ph = phenyl.

Peptide nucleic acid (PNA) is an oligonucleotide analog capable of forming highly stable complexes with its target nucleic acid [8]. One of the attractive features of PNA is its hybridization properties—a combination of high affinity and high selectivity [9] that enable its use as a probe molecule [10]. Numerous modified nucleobases have been incorporated into PNA [11]; two notable brightly fluorescent cytosine analogs are 3,5-diaza-4-oxophenothiazine (tC) [12] and phenylpyrrolocytosine (PhpC) [13], each involving multi-step ring construction syntheses (Figure 2).

With interest in simplifying the synthesis of a uracil with a biaryl-type bond to a heterocycle, we turned to the single-pot condensation of 5-formyluracil derivatives [14] with substituted 2-aminobenzamides [15]. This route has the advantage of ease of synthesis that permitted the rapid preparation and evaluation of a selection of substituted quinazolinone-uracil derivatives.

## 2. Results

### 2.1. Synthesis of Dihydroquinazolinone-Based Uracil Scaffold

**Scheme 1:**
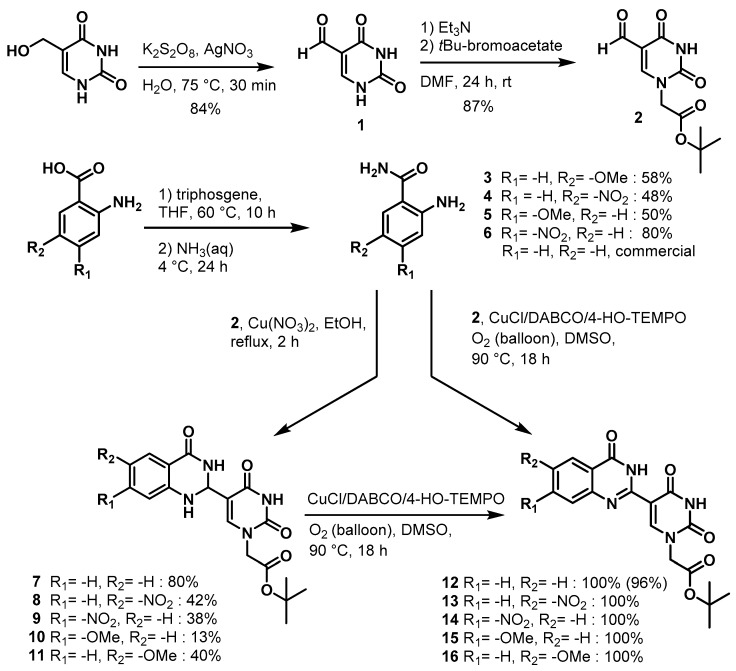
Synthesis of modified quinazolinone-based uracil scaffolds. 4-HO-TEMPO = 4-hydroxy-2,2,6,6-tetramethylpiperidin-1-oxyl; DABCO = (1,4-diazabicyclo[2.2.2]octane).

Herein, we describe a simple and convenient route for the synthesis of dihydroquinazolinone-uracil and quinazolinone-uracil scaffolds (Scheme 1), as well as an evaluation of their basic photophysical properties. The synthesis of the both the dihydroquinazolinone- and quinazolinone-uracils started with 5-formyluracil (**1**), accessed from 5-hydroxymethyluracil as previously described [14]. Next, 5-formyluracil (**1**) was alkylated under standard conditions with *tert*-butyl bromoacetate to give *tert*-butyl (5-formyluracil-1-yl)acetate (**2**) with high yield (Scheme 1).

In order to examine the effect of electron-withdrawing and electron-donating groups on the fluorescent properties of dihydroquinazolinone and quinazolinone compounds, methoxy- and nitro-substituted 2-aminobenzamide compounds were synthesized from commercially available 2-aminobenzoic acids (Scheme 1). The best yields of the 2-aminobenzamides (**3**–**6**) were achieved by performing their synthesis in two steps, i.e., the isolation of the isatoic anhydride intermediate after treatment with triphosgene and then doing the ammonolysis at a lower temperature. Subsequently, the 5-formyluracil (**2**) was condensed with the various 2-aminobenzamide compounds using copper (II) nitrate catalysis [15] to give substituted dihydroquinazolinone compounds (**7**–**11**). Compounds **7**–**11** were oxidized in high yield to the corresponding quinazolinones **12**–**16**, under optimized conditions, vide infra.

### 2.2. Synthesis of Quinazolinone-Based Uracil Scaffolds

**Scheme 2:**
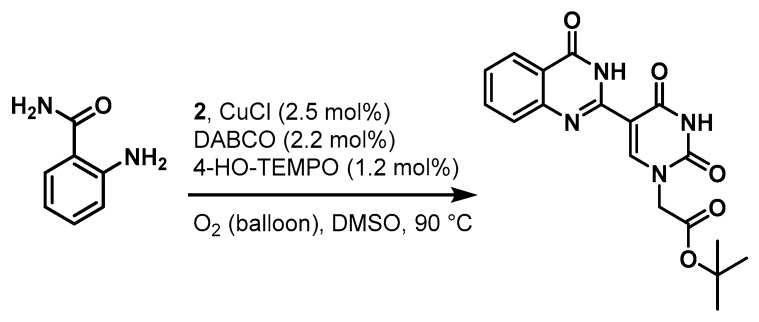
CuCl/DABCO/4-HO-TEMPO-catalyzed one-pot aerobic oxidative synthesis of quinazolinone modified uracil.

Substituted quinazolines have been synthesized by a number of methods, including the condensation of 2-aminobenzylamines with aldehydes using copper (II) nitrate in refluxing ethanol followed by subsequent treatment with a stoichiometric oxidant [16]. It has been observed in cases that the Lewis acid catalyst for the condensation may also promote oxidation. Catalytic aerobic oxidation (CuCl/DABCO/4-HO-TEMPO) has been developed for the preparation of substituted quinazolines [17], and we used this as a starting point to determine the optimal conditions for the quinazolinone-uracils of interest (Scheme 2).

We investigated the optimal conditions for the reaction of commercially available anthranilamide (R_1_ = H, R_2_ = H) (1 equiv) with *N*1-alkylated-5-formyluracil **2** (1 equiv). Firstly, the two starting materials were allowed to condense in situ, and then 4-hydroxy-2,2,6,6-tetramethylpiperidin-1-oxyl (4-HO-TEMPO) (0.2 equiv) was added as the catalyst, and the reaction system was charged with an oxygen atmosphere (balloon) [18]. The target molecule **12** was obtained, but with poor yield due to presumed decomposition of the dihydroquinazolinone intermediate (Table 1, entry **1**). To our delight, the reaction was dramatically improved when CuCl was used in combination with a monodentate *N*-containing ligand such as Et_3_N, DABCO (1,4-diazabicyclo[2.2.2]octane), or DMAP (4-dimethylaminopyridine) (Table 1, entries **4**–**8**). Entry seven demonstrates the need for an oxygen atmosphere for high conversions. The optimized conditions, (Table 1, entry **8**) were also used for the oxidation of dihydroquinazolinones **7**–**11** to quinazolinones **12**–**16**, respectively (Scheme 1).

### 2.3. Photophysical Properties

UV-Vis spectra and fluorescence measurements of compounds **7**–**16** have been made in different solvents in order to characterize their basic photophysical properties (Table 2 and Table 3). Determinations of UV-vis absorption molar extinction coefficients (ε) were made only in DMSO due to limited solubility. Overall, the values generally indicate that the quantum yield in ethanol is greater than that in other solvents for dihydroquinazolinone compounds (Table 2). Additionally, the emission is bathochromically shifted in less polar solvents (THF) versus polar solvents (EtOH). However, since dihydroquinazolinone compounds (**7**–**11**) were only very weakly emissive, they were not pursued further for use in PNA monomers or oligomers.

**Figure 3:**
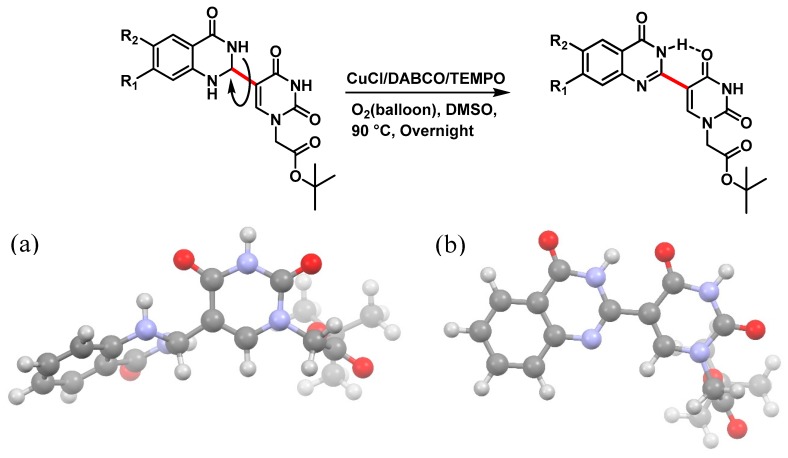
Top: chemical structures and dehydrogenation conditions. Bottom: molecular representations of the structures at minimum energy states; most energetically favorable tautomer for (**a**) dihydroquinazolinone (**b**) and quinazolinone.

The planarity and rigidity of the molecular structure plays a major role in determining the fluorescence properties of aromatic compounds [19]. Thus, the dehydrogenation of dihydroquinazolinone compounds **7**–**11** (Scheme 1 and Scheme 2) was done to increase conjugation and molecular rigidity. It was envisioned that the quinazolinone structure would favor being coplanar with the uracil by an intramolecular hydrogen bond (Figure 3a,b) and this would potentially benefit the fluorescence quantum yield. Computational studies of the two structures (Figure 3a,b) was done at the Hartree-Foch 6-31+G* level to determine the most favorable tautomer and the minimum energy conformation. The calculated structures show that the planes defined by the dihydroquinazolinone and uracil have a severe twist (~70°) about the biaryl bond (shown in red, Figure 3a), whereas the quinazolinone and uracil exists in a coplanar geometry with a hydrogen bond (predicted length 1.99 Å) between the amide proton and C4-oxygen.

The trends in the fluorescence quantum yields largely support this notion, i.e., that the quinazolinone-uracils (**12**–**16**) are more emissive than the dihydroquinzolinone congeners (**7**–**11**) (Table 3). However, the nitro-substituted quinazolinone compounds also tended to be less soluble, and measurements could not be made in ethanol. We also investigated the effects of temperature and environmental viscosity (ethanol versus glycerol) on the fluorescent properties (Table 3). The increase in fluorescence quantum yield when moving from ethanol (or other less viscous solvents) to the viscous glycerol is consistent with a reduced loss of excited state energy through intramolecular rotations, particularly with respect to the biaryl bond. Further evidence of the molecular rotor behavior is shown by the increase in quantum yield as the temperature is lowered.

### 2.4. PNA Monomer Synthesis and Oligomerization

**Scheme 3:**
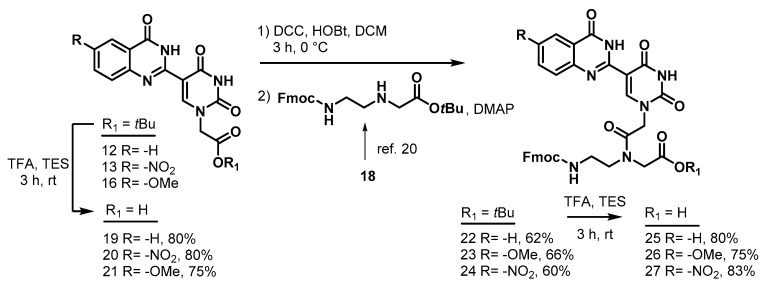
Synthesis of quinazolinone-uracil PNA monomers.

The quinazolinone-based uracil scaffolds were designed to retain the usual hydrogen bonding ability to a complementary nucleobase. In order to test the ability of these modified bases to respond to hybridization, i.e., the transition from a relatively unstructured single-stranded probe to the more highly structured environment of duplex, a selection of PNA monomers were prepared. A priori, it was not known how quinazolinone ring substitution would affect hybridization (stacking) interactions or fluorescence properties in the context of oligomers; thus, the unsubstituted quinazolinone-based uracil scaffold (**12**), the nitro-substituted quinazolinone (**13**) and the methoxy-substituted quinzaolinone (**16**) were chosen to prepare PNA monomers (Scheme 3).

Proceeding to monomer synthesis and oligomerization, the *tert*-butyl esters of the quinazolinone based uracil scaffolds (**12**, **13** and **16**) were converted to the free acids (**19**, **20** and **21**) by acidolysis with trifluoroacetic acid in the presence of triethylsilane. The free base of the *tert*-butyl-Fmoc-based aminoethylglycine backbone was liberated from the hydrochloride salt (**18**) immediately prior to use [20]. The quinazolinone-based nucleobase acetic acid was then converted to the active ester, in situ, by treatment with a solution of hydroxybenzotriazole (HOBt) and dicyclohexylcarbodiimide (DCC). The active ester of the nucleobase derivative was then coupled to the *tert*-butyl-Fmoc-based aminoethylglycine backbone in the presence of DMAP to give the monomer esters with usual yields. Finally, the monomers (**25**, **26** and **27**) were produced by the acidolysis of the *tert*-butyl esters, as previously done. The monomers were characterized using ^1^H and ^13^C NMR spectroscopy, as is usual for PNA monomer signals, indicating the presence of rotomers (see supplemental data). Additionally, the compounds were identified by high-resolution mass spectrometry (HRMS).

With monomers **25**, **26** and **27** in hand, PNA sequences were prepared by automated peptide synthesis (Table 1). The three PNA monomers performed well, with no significant difference between them and commercially available standard PNA monomers (Table S1 and Page **S5**–**S9**). As is commonly done, all of the oligomers were constructed with a C-terminal lysine in order to impart water solubility. Once the oligomers were in hand, thermal stability (T_m_) analysis with complementary DNA and PNA was undertaken.

The unmodified PNA control sequence hybridization with complementary PNA showed excellent agreement with our previous report (67.5 °C) [20]. The unmodified quinazolinone showed a slight decrease in the thermal stability of the duplex compared to that for an unmodified PNA (~∆Tm = −1.0 °C per insert), which may be useful in pseudocomplementary PNA applications as a fluorescent reporter, although this requires further study. On the other hand, both the nitro- and methoxy-substituted quinazolinones showed an increase in T_m_ values when hybridized to the PNA strand. (Table 4). Similar results were observed for hybridization with complementary (underlined) DNA (5′-AGTGATCTACCT-3′): the nitro-substituted quinazolinone had the greatest stabilizing effect (∆T_m_ = +4.0 °C per insert); next was methoxy-substituted quinazolinone (∆T_m_ = +2.0 °C per insert), while the unsubstituted quinazolinone gave a slight stabilization (∆T_m_ = +1.0 °C per insert).

### 2.5. Fluorescence Properties Analysis

**Figure 4:**
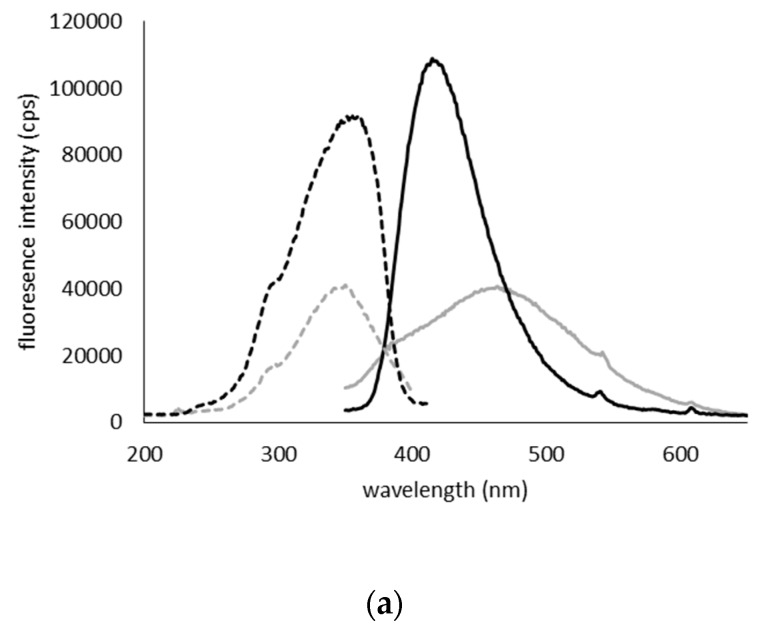

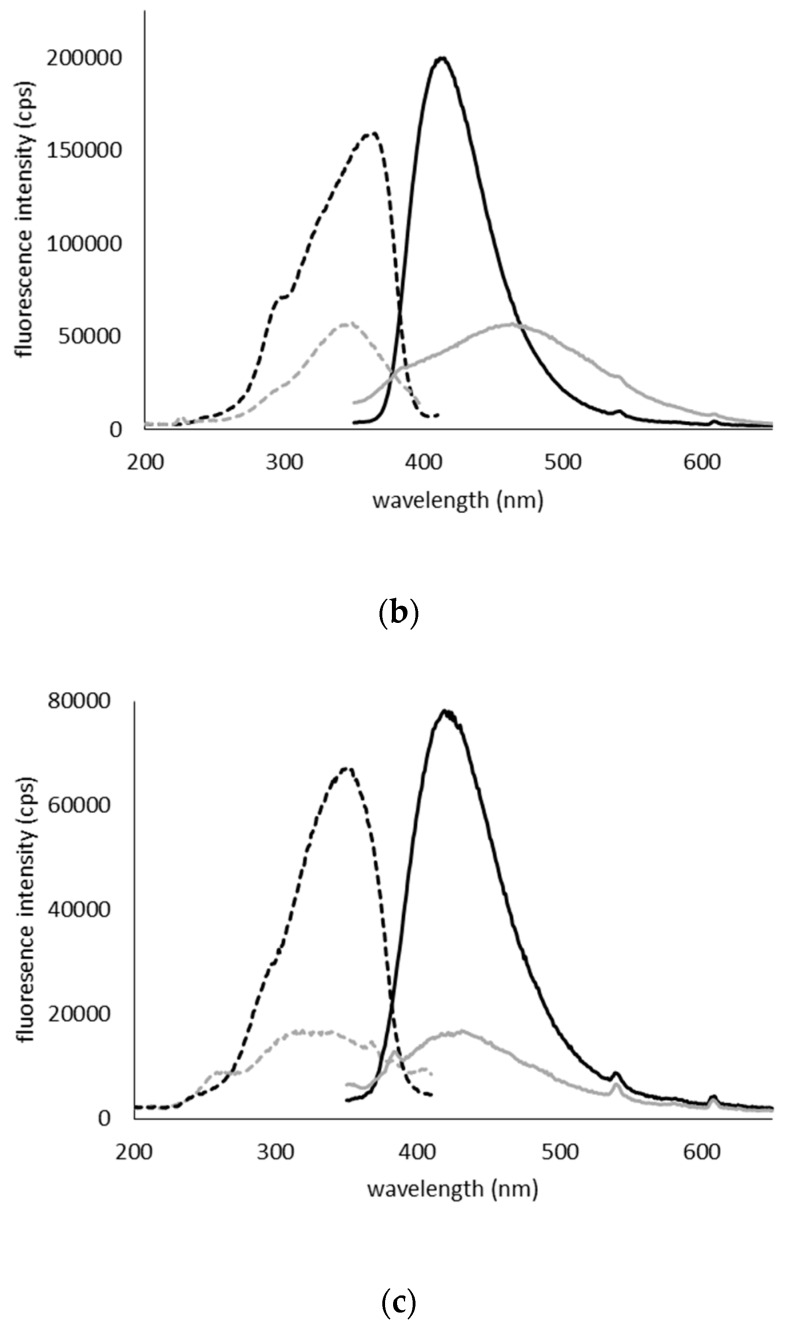
Fluorescence excitation spectra (broken lines) and emission spectra (solid lines) for the single-stranded probe containing PNAs (grey lines) and for the heteroduplexes formed with complementary PNA (black lines) at 2 µM strand concentration; 100 mM sodium phosphate buffer, pH = 7.0, 23 °C. (**a**) H-GTAGA**^Q^U**CACT-Lys-NH_2_, (**b**) H-GTAGA**^Q^U^(MeO)^**CACT-Lys-NH_2_, (**c**) H-GTAGA**^Q^U^(NO2)^**CACT-Lys-NH_2_.

The steady-state fluorescence excitation and emission spectra were measured for single-stranded PNA **^Q^U**, **^Q^U**^(OMe)^ and **^Q^U**^(NO2)^ and heteroduplexes with fully complementary DNA (Figure 4a–c). Trends similar to those in the fluorescence studies at the submonomer level were observed, that is, the nitro-substituted quinazolinone uracil scaffold shows the weakest fluorescent intensity while the methoxy-substituted has the highest. However, each of the nucleobase fluorophores displayed a “turn-on” response of approximately 3-to-4 fold to hybrid formation. Interestingly, the **^Q^U**^(NO2)^ oligomer displayed the largest fluorescence “turn-on” response, without a notable change in the peak wavelength. The **^Q^U**^(NO2)^ oligomer also shows the greatest increase in the stability of the complex as measured by the T_m_ value. A plausible explanation is that the **^Q^U**^(NO2)^ oligomer has relatively stronger stacking interactions, leading to both a more stabilized hybrid and a relatively more rigid structure, providing a slightly better fluorescence turn-on effect. The more electron-rich parent quinazolinone and 6-methoxyquinazolinone both displayed a hypsochromic shift in the emission for the hybrid, which likely reflects the change in the polarity of the environment of the fluorophore. Nonetheless, all three fluorophores showed marked increases in emission upon hybridization, which is consistent with increased rigidity in the duplex and the molecular rotor behavior of biaryl-chromophores. The degree of fluorescence intensity in quinazolinone uracil scaffolds appears to weakly correlate with the helix-stabilizing ability of the base. The most stable heteroduplex occurs with **^Q^U**^(NO2)^-modified PNA.

## 3. Materials and Methods

### 3.1. Synthesis and Characterization

**5-Formyluracil (1)**


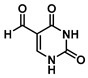


5-Hydroxymethyluracil (4.0 g, 28 mmol), was dissolved in 100 mL water by heating to 75 °C. The solution was allowed to cool to 40 °C, and then K_2_S_2_O_8_ (13.6 g, 49.6 mmol) and AgNO_3_ (0.14 g, 0.8 mmol) were added and the product began to slowly precipitate. The reaction was stirred for 15 min at 35 °C and then for 10 min at room temperature. The mixture was held at 4 °C for 1 h, after which time the product was collected by filtration, washed with cold water and dried to yield **1** (3.2 g, 22.7 mmol, 84%) as a white solid. The spectra matched the literature values [14]. ^1^H NMR (400 MHz, DMSO-*d*_6_) δ 11.96 (s, 1H), 11.50 (s, 1H), 9.74 (s, 1H), 8.13 (s, 1H). ^13^C}^1^H} NMR (101 MHz, DMSO-*d*_6_) δ 186.9, 162.9, 150.9, 149.7, 110.6.

***tert*-Butyl (5-formyluracil-1-yl)acetate (2)**


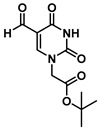


5-Formyluracil **1** (2.0 g, 14.3 mmol) was added to 50 mL dry dimethylformamide (DMF), heated to 40 °C to dissolve and then allowed to cool to room temperature. Triethylamine (NEt_3_, 2.0 mL, 14.3 mmol) was added to the solution in one portion. *tert*-Butyl bromoacetate (2.11 mL, 14.3 mmol) was added dropwise to the stirred mixture over 30 min and then stirring was continued for 24 h under an N_2_ atmosphere. The solvent was removed in vacuo and the residue was extracted with ethyl acetate and water. The organic layer was washed with brine, dried by Na_2_SO_4_ and concentrated in vacuo to give **2** as a pure, white solid product (3.16 g, 12.43 mmol, 87%). Spectroscopic analysis conformed to previous reports [21]. ^1^H NMR (400 MHz, DMSO-*d*_6_) δ 11.92 (s, 1H), 9.79 (s, 1H), 8.51 (s, 1H), 4.56 (s, 2H), 4.02 (q, *J* = 7.1 Hz, 1H), 1.98 (s, 1H), 1.42 (s, 12H). ^13^C}^1^H} NMR (101 MHz, DMSO-*d*_6_) δ 186.8, 167.0, 164.1, 163.7, 162.7, 154.1, 152.4, 150.4, 110.7, 102.6, 82.8, 60.3, 50.5, 50.4, 28.0, 27.9, 14.5.

**2-Amino-5-methoxybenzamide (3)**


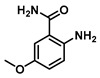


2-Amino-5-methoxybenzoic acid (0.35 g, 2.5 mmol) was dissolved in 4 mL tetrahydrofuran (THF) and heated to 60 °C, and then triphosgene (0.25 g, 0.84 mmol) was added and the reaction was stirred for 10 h. The reaction progress was monitored by TLC analysis. The solution cooled down to room temperature, and the isatoic anhydride intermediate was filtered and collected. Cold 1N ammonia was added to the collected intermediate, and the mixture was stirred for 24 h at 4 °C. To the crude solution were added 10 mL of EtOAc/Hexanes (1:1) mixture, and the resulting precipitate was filtered and washed sequentially with water and diethyl ether to give 0.240 g (1.4 mmol, 58%) of Compound **3** as a brown solid. Spectra matched those reported by the Integrated Spectral Database System of Organic Compounds (AIST, Tokio, Japan). ^1^H NMR (400 MHz, DMSO-*d*_6_) δ 7.75 (s, 1H), 7.10 (d, *J* = 2.9 Hz, 1H), 7.08 (s, 1H), 6.84 (dd, *J* = 8.9, 2.9 Hz, 1H), 6.64 (d, *J* = 8.9 Hz, 1H), 6.12 (s, 2H), 3.68 (s, 3H). ^13^C}^1^H} NMR (101 MHz, DMSO-*d*_6_) δ 171.4, 149.6, 144.9, 120.3, 118.2, 114.4, 112.9, 56.0.

**2-Amino-5-nitrobenzamide (4)**


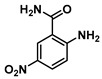


2-Amino-5-nitrobenzoic acid (0.785 g, 4.5 mmol) was dissolved in 10 mL of THF and heated to 60 °C, triphosgene (0.45 g, 1.5 mmol) was added and the reaction was stirred for 10 h. The reaction progress was monitored by TLC analysis. The solution was cooled to room temperature, and the isatoic anhydride intermediate was filtered and collected. Cold 1N ammonia was added to the collected intermediate, and the mixture was stirred for 24 h at 4 °C. The turbid solution was filtered and washed with water and ether to give 0.430 g (2.3 mmol, 48%) of Compound **4** as a yellow solid. Spectra matched those reported by the Integrated Spectral Database System of Organic Compounds (AIST, Japan). ^1^H NMR (400 MHz, DMSO-*d*_6_) δ 8.56 (d, *J* = 2.6 Hz, 1H), 8.21 (s, 1H), 8.02 (dd, *J* = 9.2, 2.6 Hz, 1H), 7.90 (s, 2H), 7.41 (s, 1H), 6.80 (d, *J* = 9.2 Hz, 1H). ^13^C}^1^H} NMR (101 MHz, DMSO-*d*_6_) δ 170.1, 156.2, 135.2, 128.0, 126.9, 116.4, 112.5.

**2-Amino-4-methoxybenzamide (5)**


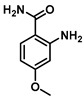


2-Amino-4-methoxybenzoic acid (0.501 g, 3.0 mmol) was dissolved in 6 mL THF and heated to 60 °C, triphosgene (0.3 g, 1 mmol) was added and the reaction was stirred for 10 h. The reaction progress was monitored by TLC analysis. Cold 1N ammonia was added to the collected intermediate, and the mixture was stirred for 24 h at 4 °C. The turbid solution was filtered and washed with water and ether to give 0.25 g (1.5 mmol, 50%) of Compound **5** as a white solid. Spectra matched those reported by the Integrated Spectral Database System of Organic Compounds (AIST, Japan). ^1^H NMR (400 MHz, DMSO-*d*_6_) δ 7.49 (d, *J* = 8.8 Hz, 1H), 6.74 (s, 2H), 6.20 (d, *J* = 2.6 Hz, 1H), 6.07 (dd, *J* = 8.8, 2.6 Hz, 1H), 3.70 (s, 3H).

**2-Amino-4-nitrobenzamide (6)**


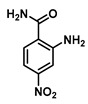


2-Amino-4-nitrobenzoic acid (0.549 g, 3.0 mmol) was dissolved in 6 mL THF and, heated to 60 °C, triphosgene (0.3 g, 1 mmol) was added, the reaction was stirred for 5 h and the reaction progress was monitored by TLC. The solution was cooled to room temperature, and the isatoic anhydride intermediate was filtered and collected. Cold 1N ammonia was added to the collected intermediate, and the mixture was stirred for 24 h at 4 °C. The turbid solution was filtered and washed with water and diethyl ether to give 0.430 g (2.3 mmol, 80%) of Compound **6** as an orange solid. Spectra matched those reported by the Integrated Spectral Database System of Organic Compounds (AIST, Japan). ^1^H NMR (400 MHz, DMSO-*d*_6_) δ 8.03 (s, 1H), 7.74 (d, *J* = 8.6 Hz, 1H), 7.57 (d, *J* = 2.4 Hz, 1H), 7.46 (s, 1H), 7.25 (dd, *J* = 8.6, 2.4 Hz, 1H), 7.02 (s, 2H).

***tert*-Butyl 2-(5-(4-oxo-1,2,3,4-tetrahydroquinazolin-2-yl)uracil-1-yl)acetate (7)**


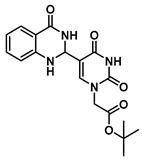


To a solution of 0.50 g (2.0 mmol) of **2** dissolved in 10 mL of reflux ethanol were added 36 mg (0.13 mmol) of Cu(NO_3_)_2_·3H_2_O and 0.14 g (1.0 mmol) of 2-aminobenzamide. The reaction mixture was stirred for 2 h and analyzed by TLC. The solution was cooled to room temperature, and the resulting precipitate was filtered and washed sequentially with EtOH and MeOH to give **7** (0.298 g, 0.8 mmol, 80%) as a white solid. ^1^H NMR (400 MHz, DMSO-*d*_6_) δ 11.62 (s, 1H), 7.95 (d, *J* = 1.8 Hz, 1H), 7.82 (s, 1H), 7.62 (dd, *J* = 7.8, 1.6 Hz, 1H), 7.25 (ddd, *J* = 8.5, 7.2, 1.7 Hz, 1H), 6.78 (d, *J* = 8.1 Hz, 1H), 6.75–6.66 (m, 2H), 5.68 (d, *J* = 2.0 Hz, 1H), 4.48 (s, 2H), 1.40 (s, 9H). ^13^C}^1^H} NMR (101 MHz, DMSO-*d*_6_) δ 167.5, 163.9, 163.3, 150.9, 148.2, 144.5, 133.7, 127.9, 117.9, 115.3, 112.9, 82.2, 60.4, 50.1, 28.1. HRMS (ESI/Q-TOF) *m*/*z*: [M]^+^ calculated for C_18_H_20_N_4_O_5_, 372.1434; found, 372.1428.

***tert*-Butyl 2-(5-(6-nitro-4-oxo-1,2,3,4-tetrahydroquinazolin-2-yl)uracil-1-yl) acetate (8)**


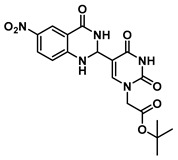


To a solution of 0.50 g (2.0 mmol) of **2** dissolved in 10 mL of refluxing ethanol, 36.2 mg (0.15 mmol) of Cu(NO_3_)_2_·3H_2_O and 0.18 g (1 mmol) of 2-amino-5-nitrobenzamide were added, and the reaction mixture was stirred for 2 h and monitored by TLC. The solution was cooled to room temperature, and the resulting precipitate was filtered and washed with EtOH and MeOH to give **8** (176 mg, 0.42 mmol, 42%) as a yellow solid. ^1^H NMR (400 MHz, DMSO-*d*_6_) δ 11.67 (s, 1H), 8.41 (dd, *J* = 8.3, 2.4 Hz, 2H), 8.30 (s, 1H), 8.09 (dd, *J* = 9.1, 2.8 Hz, 1H), 7.81 (s, 1H), 6.84 (d, *J* = 9.1 Hz, 1H), 5.85 (t, *J* = 2.1 Hz, 1H), 4.47 (s, 2H), 1.39 (s, 9H). ^13^C}^1^H} NMR (101 MHz, DMSO-*d*_6_) δ 167.7, 162.9, 161.6, 152.4, 150.9, 144.4, 137.5, 129.2, 124.5, 114.9, 113.0, 112.9, 82.3, 61.0, 49.9, 28.1. HRMS (ESI/Q-TOF) *m*/*z*: [M]^+^ calculated for C_18_H_19_N_5_O_7_, 417.1284; found, 417.1271.

***tert*-Butyl 2-(5-(7-nitro-4-oxo-1,2,3,4-tetrahydroquinazolin-2-yl)uracil-1-yl)acetate (9)**


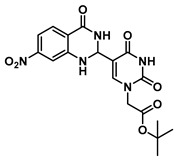


To a solution of 0.50 g (2.0 mmol) of **2** dissolved in 10 mL of refluxing ethanol, 36.2 mg (0.15 mmol) of Cu(NO_3_)_2_·3H_2_O and 0.18 g (1 mmol) of 2-amino-4-nitrobenzamide were added, and the reaction mixture was stirred for 2 h and monitored by TLC. The solution was cooled down to room temperature, and the resulting precipitate was filtered and washed with EtOH and MeOH to give **9** (160 mg, 0.38 mmol, 38%) as a yellow solid. ^1^H NMR (600 MHz, DMSO-*d*_6_) δ 11.62 (s, 1H), 8.34 (s, 1H), 7.79 (d, *J* = 8.5 Hz, 1H), 7.74 (s, 1H), 7.59 (d, *J* = 2.3 Hz, 1H), 7.43–7.37 (m, 2H), 5.73 (s, 1H), 4.43 (s, 2H), 1.34 (s, 9H). ^13^C}^1^H} NMR (101 MHz, DMSO-*d*_6_) δ 166.5, 162.9, 161.6, 152.4, 150.9, 144.4, 137.5, 129.2, 124.5, 114.8, 113.0, 112.9, 82.3, 61.0, 49.9, 27.3. HRMS (ESI/Q-TOF) *m*/*z*: [M]^+^ calculated for C_18_H_19_N_5_O_7_, 417.1284; found, 417.1270.

***tert*-Butyl 2-(5-(7-methoxy-4-oxo-1,2,3,4-tetrahydroquinazolin-2-yl)uracil-1-yl)acetate (10)**


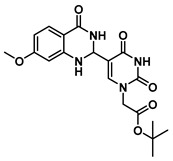


To a solution of 0.25 g (1.0 mmol) of **2** dissolved in 10 mL of refluxing ethanol, 18.1 mg (0.075 mmol) of Cu(NO_3_)_2_·3H_2_O and 83 mg (0.5 mmol) of 2-amino-4-methoxybenzamide were added, and the reaction mixture was stirred for 10 h and monitored by TLC. The solution was cooled to room temperature, and the resulting precipitate was filtered and washed with EtOH and MeOH to give **10** (51 mg, 0.13 mmol, 13%) as a yellow solid. ^1^H NMR (400 MHz, DMSO-*d*_6_) δ 12.37 (s, 1H), 12.03 (s, 1H), 9.02 (s, 1H), 8.02 (d, *J* = 8.7 Hz, 1H), 7.12–7.01 (m, 2H), 4.70 (s, 2H), 3.89 (s, 3H), 1.45 (s, 9H). ^13^C}^1^H} NMR (101 MHz, DMSO-*d*_6_) δ 167.2, 164.6, 164.5, 160.1, 151.3, 150.7, 150.1, 149.6, 128.1, 116.1, 114.8, 108.2, 103.2, 82.7, 56.1, 50.7, 28.1. HRMS (ESI/Q-TOF) *m*/*z*: [M]^+^ calculated for C_19_H_22_N_4_O_6_, 402.1539; found, 402.1536.

***tert*-Butyl 2-(5-(6-methoxy-4-oxo-1,2,3,4-tetrahydroquinazolin-2-yl)uracil-1-yl)acetate (11)**


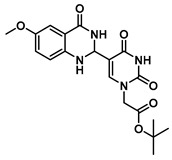


To a solution of 0.25 g (1 mmol) of **2** dissolved in 10 mL of refluxing ethanol, 18.1 mg (0.075 mmol) of Cu(NO_3_)_2_·3H_2_O and 83 mg (0.5 mmol) of 2-amino-5-methoxybenzamide were added, and the reaction mixture was stirred for 4 h and monitored by TLC. The solution was cooled to room temperature, and the resulting precipitate was filtered and washed with EtOH and MeOH to give **11** (155 mg, 0.40 mmol, 40%) as a yellow solid. ^1^H NMR (400 MHz, DMSO-*d*_6_) δ 11.61 (s, 1H), 8.00 (t, *J* = 1.8 Hz, 1H), 7.82 (s, 1H), 7.16 (d, *J* = 3.0 Hz, 1H), 6.93 (dd, *J* = 8.7, 3.0 Hz, 1H), 6.77 (d, *J* = 8.8 Hz, 1H), 6.38 (d, *J* = 1.7 Hz, 1H), 5.62 (t, *J* = 1.7 Hz, 1H), 4.48 (s, 2H), 3.69 (s, 3H), 1.40 (s, 9H). ^13^C}^1^H} NMR (101 MHz, DMSO-*d*_6_) δ 167.5, 163.9, 163.3, 152.2, 150.9, 144.5, 142.5, 121.8, 116.9, 116.0, 112.7, 110.4, 82.2, 60.6, 55.7, 50.0, 28.1. HRMS (ESI/Q-TOF) *m*/*z*: [M]^+^ calculated for C_19_H_22_N_4_O_6_, 402.1539; found, 402.1537.

***tert*-Butyl 2-(5-(4-oxo-3,4-dihydroquinazolin-2-yl)uracil-1-yl)acetate (12)**


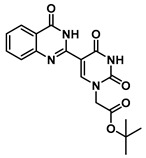


To a solution of 0.15 g, (0.4 mmol) of **7** fully dissolved in 1 mL of DMSO at 60 °C were added CuCl (1.2 mg, 0.01 mmol), 4-HO-TEMPO (1 mg, 0.005 mmol) and DABCO (1 mg, 0.009 mmol). The mixture was stirred at 90 °C for 18 h under an atmosphere of O_2_ (balloon). After the completion of the reaction (monitored by TLC), the reaction was cooled to room temperature and the resulting precipitate was washed with water and ethanol to give the product **12** (0.15 g, 0.4 mmol, 100%) as a white solid. ^1^H NMR (400 MHz, DMSO-*d*_6_) δ 12.46 (s, 1H), 9.27 (s, 1H), 8.38 (d, *J* = 7.9 Hz, 1H), 8.08 (t, *J* = 7.7 Hz, 1H), 7.90 (d, *J* = 8.2 Hz, 1H), 7.74 (t, *J* = 7.5 Hz, 1H), 4.96 (s, 2H), 1.71 (s, 9H). ^13^C}^1^H} NMR (151 MHz, DMSO-*d*_6_) δ 166.8, 164.3, 150.5, 150.0, 149.1, 134.8, 126.4, 126.3, 121.5, 110.0, 103.5, 82.8, 50.8, 40.8, 40.7, 40.6, 40.4, 40.3, 40.2, 40.0, 28.2. HRMS (ESI/Q-TOF) *m*/*z*: [M]^+^ calculated for C_18_H_18_N_4_O_5_, 370.1277; found, 370.1279.

***tert*-Butyl 2-(5-(6-nitro-4-oxo-1,4-dihydroquinazolin-2-yl)uracil-1-yl)acetate (13)**


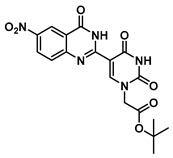


To a solution of 0.13 g (0.31 mmol) of **8** in 1 mL of DMSO dissolved at 60 °C were added CuCl (2.9 mg, 0.02 mmol), 4-HO-TEMPO (2 mg, 0.012 mmol) and DABCO (2 mg, 0.022 mmol). The mixture was stirred at 90 °C overnight under an atmosphere of O_2_ (balloon). After the completion of the reaction (monitored by TLC), the reaction was cooled to room temperature and the resulting precipitate was washed with water and ethanol to give the product **13** as a yellow solid (0.13 g, 0.31 mmol, 100%). ^1^H NMR (400 MHz, DMSO-*d*_6_) δ 12.51 (s, 1H), 9.15 (s, 1H), 8.80 (d, *J* = 2.7 Hz, 1H), 8.55 (dd, *J* = 9.0, 2.8 Hz, 1H), 7.77 (d, *J* = 9.0 Hz, 1H), 4.74 (s, 2H), 1.46 (s, 9H). ^13^C}^1^H} NMR (101 MHz, DMSO-*d*_6_) δ 167.1, 159.9, 152.2, 144.6, 129.2, 128.7, 122.6, 121.2, 82.8, 50.9, 28.1. HRMS (ESI/Q-TOF) *m*/*z*: [M]^+^ calculated for C_18_H_17_N_5_O_7_, 415.1128; found, 415.1130.

***tert*-Butyl 2-(5-(7-nitro-4-oxo-1,4-dihydroquinazolin-2-yl)uracil-1-yl)acetate (14)**


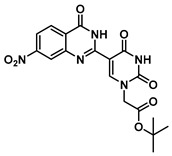


To 1 mL of of DMSO was added 0.12 g (0.29 mmol) of **9**, and the temperature was increased to 60 °C until a clear solution was obtained. To the same solution were added CuCl (2.9 mg, 0.02 mmol), 4-HO-TEMPO (2 mg, 0.012 mmol) and DABCO (2 mg, 0.022 mmol), and the mixture was stirred at 90 °C overnight under O_2_ atmosphere (balloon). After the completion of the reaction (monitored by TLC), the reaction was cooled down to room temperature and the resulting precipitate was washed with water and ethanol to give the product **14** as a yellow solid (0.12 g, 0.29 mmol, 100%).^1^H NMR (400 MHz, DMSO-*d*_6_) δ 12.50–12.39 (m, 2H), 9.12 (s, 1H), 8.36–8.27 (m, 2H), 8.18 (dd, *J* = 8.7, 2.2 Hz, 1H), 4.72 (s, 2H), 1.46 (s, 9H). ^13^C}^1^H} NMR (101 MHz, DMSO-*d*_6_) δ 167.1, 159.7, 151.8, 151.6, 151.4, 150.0, 149.6, 128.8, 125.7, 121.9, 119.8, 102.7, 82.8, 50.9, 28.1. HRMS (ESI/Q-TOF) *m*/*z*: [M]^+^ calculated for C_18_H_17_N_5_O_7_, 415.1128; found, 415.1133.

***tert*-Butyl 2-(5-(7-methoxy-4-oxo-1,4-dihydroquinazolin-2-yl)uracil-1-yl)acetate (15)**


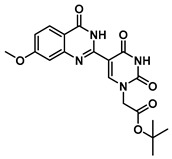


To 1 mL of of DMSO was added 0.14 g (0.35 mmol) of **10**, and the temperature was increased to 60 °C until a clear solution was obtained. To the same solution were added CuCl (2.9 mg, 0.02 mmol), 4-HO-TEMPO (2 mg, 0.012 mmol) and DABCO (2 mg, 0.022 mmol), and the mixture was stirred at 90 °C overnight under an O_2_ atmosphere (balloon). After the completion of the reaction (monitored by TLC), the reaction was cooled down to room temperature and the resulting precipitate was washed with water and ethanol to give the product **15** as a yellow solid (0.14 g, 0.35 mmol, 100%). ^1^H NMR (400 MHz, DMSO-*d*_6_) δ 12.37 (s, 1H), 12.03 (s, 1H), 9.02 (s, 1H), 8.02 (d, *J* = 8.7 Hz, 1H), 7.12–7.01 (m, 2H), 4.70 (s, 2H), 3.89 (s, 3H), 1.45 (s, 9H). ^13^C }^1^H} NMR (101 MHz, DMSO-*d*_6_) δ 167.2, 164.7, 164.5, 160.1, 151.3, 150.7, 150.1, 149.7, 128.1, 116.1, 114.8, 108.2, 103.2, 82.7, 56.1, 50.7, 28.1. HRMS (ESI/Q-TOF) *m*/*z*: [M]^+^ calculated for C_19_H_20_N_4_O_6_, 400.1383; found, 400.1379.

***tert*-Butyl 2-(5-(6-methoxy-4-oxo-3,4-dihydroquinazolin-2-yl)uracil-1-yl)acetate (16)**


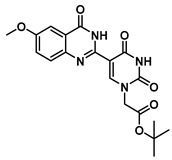


To 1 mL of of DMSO was added 0.14 g (0.35 mmol) of **11**, and the temperature was increased to 60 °C until a clear solution was obtained. To the same solution were added CuCl (2.9 mg, 0.02 mmol), 4-HO-TEMPO (2 mg, 0.012 mmol) and DABCO (2 mg, 0.022 mmol), and the mixture was stirred at 90 °C overnight under an O_2_ atmosphere (balloon). After the completion of the reaction (monitored by TLC), the reaction was cooled down to room temperature and the resulting precipitate was washed with water and ethanol to give the product **16** as a yellow solid (0.14 g, 0.35 mmol, 100%). ^1^H NMR (400 MHz, DMSO-*d*_6_) δ 12.37 (s, 1H), 12.03 (s, 1H), 9.02 (s, 1H), 8.02 (d, *J* = 8.7 Hz, 1H), 7.12–7.01 (m, 2H), 4.70 (s, 2H), 3.89 (s, 3H), 1.45 (s, 9H). ^13^C}^1^H} NMR (101 MHz, DMSO-*d*_6_) δ 167.2, 164.7, 164.5, 160.1, 151.3, 150.7, 150.1, 149.7, 128.1, 116.1, 114.8, 108.2, 103.2, 82.7, 56.1, 50.7, 28.1. HRMS (ESI/Q-TOF) *m*/*z*: [M]^+^ calculated for C_19_H_20_N_4_O_6_, 400.1383; found, 400.1382.

***tert*-Butyl (2-aminoethyl)glycinate (17)**


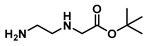


*tert*-Butyl bromoacetate (6.9 mL, 47 mmol) was mixed with 37.5 mL of dichloromethane and added dropwise to a mixture of 25 mL (0.37 mol) of ethylenediamine (dissolved in 175 mL of dichloromethane) in an ice bath. The reaction continued for 19 h. Then, it was extracted with water (90 mL × 2) and dichloromethane (60 mL × 3), dried with sodium sulphate and concentrated in vacuo to give 15.8 g (90 mmol, 51%) of ester **17** as a colorless liquid. ^1^H NMR (400 MHz, Chloroform-*d*) δ 3.22 (s, 2H), 2.74–2.66 (m, 2H), 2.64–2.52 (m, 2H), 1.39 (s, 9H).

***tert*-Butyl (2-((((9H-fluoren-9-yl)methoxy)carbonyl)amino)ethyl)glycinate (18)**


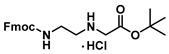


Ester **17** (15.0 g, 88 mmol) and 15.2 mL of diisopropylethylamine (90 mmol) were dissolved in 680 mL of dichloromethane. To this solution was added, dropwise over the course of 70 min, a solution of 26.3 g (77 mmol) of Fmoc-OSu dissolved in 192 mL of dichloromethane. The reaction mixture was stirred overnight and washed with 1 M HCl (3 × 100 mL) and brine (100 mL). The organic layer was dried with sodium sulphate and filtered. The solution was partially concentrated to 50 mL in vacuo and cooled in a freezer (−20 °C) overnight. The white precipitate was filtered the next day and washed with dichloromethane and mother liquor and then returned to the freezer to generate more of product **18** (8.0 g, 19 mmol, 20%) as a white precipitate. ^1^H NMR (400 MHz, DMSO-*d*_6_) δ 7.91–7.80 (m, 3H), 7.68 (dd, *J* = 16.3, 7.9 Hz, 2H), 7.46–7.23 (m, 5H), 4.36–4.27 (m, 2H), 4.22 (q, *J* = 7.3 Hz, 1H), 3.20 (s, 1H), 3.07 (q, *J* = 6.2 Hz, 2H), 2.58 (t, *J* = 6.4 Hz, 1H), 1.41 (s, 9H). ^13^C}^1^H} NMR (101 MHz, DMSO-*d*_6_) δ 171.9, 156.7, 144.4, 141.2, 129.4, 128.0, 127.7, 127.5, 125.6, 121.8, 120.6, 120.5, 110.1, 80.5, 65.8, 51.3, 48.7, 47.2, 40.8, 28.2. This corresponded to the NMR spectra previously reported in the literature [22].

**2-(2,4-dioxo-5-(4-oxo-3,4-dihydroquinazolin-2-yl)uracil-1-yl)acetic acid (19)**


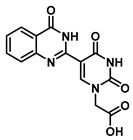


To 0.1 g (0.27 mmol) of ester **12** were added TFA (1 mL) and triethylsilane (three drops). The reaction was stirred for 2 h at room temperature, then the excess acid was evaporated under a nitrogen stream, and the product was washed with hexanes and ethyl ether to give acid **19** as a yellow solid (67 mg, 0.21 mmol, 80%). ^1^H NMR (400 MHz, DMSO-*d*_6_) 12.66 (s, 1H), 9.27 (s, 1H), 8.38 (d, *J* = 7.9 Hz, 1H), 8.08 (t, *J* = 7.7 Hz, 1H), 7.92 (d, *J* = 8.2 Hz, 1H), 7.64 (t, *J* = 7.5 Hz, 1H), 4.86 (s, 2H). ^13^C}^1^H} NMR (101 MHz, DMSO-*d*_6_) δ 169.4, 166.8, 164.3, 150.5, 150.0, 149.1, 134.8, 126.4, 126.3, 121.5, 110.0, 103.5, 82.8, 50.8. HRMS (ESI/Q-TOF) *m*/*z*: [M]^+^ calculated for C_14_H_10_N_4_O_5_, 314.0651; found, 314.0658.

**2-(5-(6-nitro-4-oxo-3,4-dihydroquinazolin-2-yl)uracil-1-yl)acetic acid (20)**


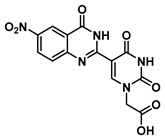


To 0.1 g (0.24 mmol) of ester **13** were added TFA (1 mL) and triethylsilane (three drops). The reaction was stirred for 2 h at room temperature, then the excess acid was evaporated under a nitrogen stream, and the product was washed with hexane and ethyl ether to give acid **20** as a yellow solid (70 mg, 0.19 mmol, 80%). ^1^H NMR (400 MHz, DMSO-*d*_6_) δ 13.39 (s, 1H), 12.49 (s, 1H), 9.16 (d, *J* = 1.4 Hz, 1H), 8.78 (q, *J* = 2.6 Hz, 1H), 8.54 (dq, *J* = 9.1, 2.6 Hz, 1H), 7.80–7.72 (m, 2H), 4.76 (s, 2H). ^13^C}^1^H} NMR (101 MHz, DMSO-*d*_6_) δ 169.4, 164.5, 160.0, 152.6, 152.3, 149.9, 144.5, 129.2, 128.7, 122.6, 121.2, 102.4, 56.5, 50.3, 19.0. HRMS (ESI/Q-TOF) *m*/*z*: [M]^+^ calculated for C_14_H_9_N_5_O_7_, 359.0502; found, 359.0509.

**2-(5-(6-methoxy-4-oxo-3,4-dihydroquinazolin-2-yl)uracil-1-yl)acetic acid (21)**


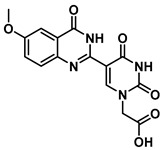


To 0.1 g (0.25 mmol) of ester **16** were added TFA (1 mL) and triethylsilane (three drops). The reaction was stirred for 2 h at room temperature, then the excess acid was evaporated under a nitrogen stream, and the product was washed with hexane and ethyl ether to give acid **21** as a light green solid (65 mg, 0.19 mmol, 75%). ^1^H NMR (400 MHz, DMSO-*d*_6_) δ 12.32 (s, 1H), 12.13 (s, 1H), 8.95 (s, 1H), 7.59 (d, *J* = 8.9 Hz, 1H), 7.51 (d, *J* = 3.0 Hz, 1H), 7.43 (dd, *J* = 8.9, 3.0 Hz, 1H), 4.72 (s, 2H), 3.88 (s, 3H). ^13^C}^1^H} NMR (101 MHz, DMSO-*d*_6_) δ 169.6, 164.5, 160.4, 157.8, 150.2, 150.1, 146.9, 143.6, 128.9, 124.8, 122.1, 106.4, 103.5, 56.1, 50.0. HRMS (ESI/Q-TOF) *m*/*z*: [M]^+^ calculated for C_15_H_12_N_4_O_6_, 344.0757; found, 344.0759.

***tert*-Butyl N-(2-((((9H-fluoren-9-yl)methoxy)carbonyl)amino)ethyl)-N-(2-(2,4-dioxo-5-(4-oxo-3,4-dihydroquinazolin-2-yl)uracil-1-yl)acetyl)glycinate (22)**


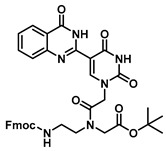


In 2 mL of DMF at 0 °C were dissolved 60 mg (0.19 mmol) of **19**, HOBt (24 mg, 0.18 mmol) and DCC (67 mg, 0.32 mmol). This mixture was stirred until the formation of precipitation was observed, then 70 mg (0.15 mmol) of **6** as a free base with DMAP (1.8 mg, 0.015 mmol) were added, and the reaction continued for 3 h and was checked by TLC. The mixture was diluted with saturated aqueous NaHCO_3_, extracted with dichloromethane (DCM), washed with brine, concentrated in vacuo and purified by column chromatography (1:1 to 4:1 ethyl acetate/hexanes) to yield **22** as a white solid (81 mg, 0.1 mmol, 62%). ^1^H NMR (400 MHz, DMF-*d*_7_) δ 8.38 (dd, *J* = 7.7, 3.6 Hz, 3H), 8.09 (dd, *J* = 10.0, 7.5 Hz, 3H), 7.84 (dd, *J* = 8.6, 6.2 Hz, 3H), 7.79–7.71 (m, 3H), 4.85–4.57 (m, 4H), 4.43 (d, *J* = 4.0 Hz, 2H), 4.02 (d, *J* = 2.4 Hz, 1H), 3.38–3.27 (m, 3H), 2.76 (q, *J* = 7.3 Hz, 1H), 1.84 (s, 9H). ^13^C{^1^H} NMR (101 MHz, DMF-*d*_7_) δ 169.99, 153.96, 144.68, 141.65, 129.81, 128.57, 128.17, 128.01, 128.00, 125.97, 122.27, 121.03, 110.63, 81.40, 68.20, 67.64, 67.23, 55.58, 55.30, 48.40, 47.59, 33.9, 31.2, 28.1. HRMS (ESI/Q-TOF) *m*/*z*: [M + Na]^+^ calculated for C_37_H_36_N_6_O_8_Na, 715.2492; found, 715.2487.

***tert*-Butyl *N*-(2-((((9H-fluoren-9-yl)methoxy)carbonyl)amino)ethyl)-*N*-(2-(5-(6-methoxy-4-oxo-3,4-dihydroquinazolin-2-yl)uracil-1-yl)acetyl)glycinate (23)**


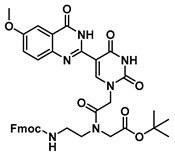


To 0.1 g (0.29 mmol) of **21** dissolved in 2 mL of DMF at 0 °C were added HOBt (26 mg, 0.20 mmol) and DCC (70 mg, 0.34 mmol). This reaction was stirred until the formation of precipitation was observed, then 70 mg (0.15 mmol) of **6** as a free base with DMAP (1.8 mg, 0.015 mmol) were added, and the reaction continued for 3 h and was checked by TLC. The mixture was diluted with saturated aqueous NaHCO_3_, extracted with DCM, washed with brine, concentrated in vacuo and purified by column chromatography (1:1 to 4:1 ethyl acetate/hexanes) to yield **23** as a white solid (77 mg, 0.1 mmol, 66%). ^1^H NMR (400 MHz, DMF-*d*_7_) δ 8.31 (dd, *J* = 7.7, 3.6 Hz, 3H), 8.07 (dd, *J* = 10.0, 7.5 Hz, 3H), 7.84 (dd, *J* = 8.6, 6.2 Hz, 3H), 7.79–7.71 (m, 3H), 4.83–4.59 (m, 4H), 4.43 (d, *J* = 4.0 Hz, 2H), 4.01 (d, *J* = 2.4 Hz, 1H), 3.75 (s, 3H), 3.36–3.26 (m, 3H), 2.76 (q, *J* = 7.3 Hz, 1H), 1.84 (s, 9H). ^13^C{^1^H} NMR (101 MHz, DMF-*d*_7_) δ 169.99, 153.96, 144.68, 141.65, 129.81, 128.57, 128.17, 128.01, 128.00, 125.97, 122.27, 121.03, 110.63, 81.40, 68.20, 67.64, 67.23, 55.58, 55.30, 52.05, 48.40, 47.59, 32.9, 30.2, 28.1. HRMS (ESI/Q-TOF) *m*/*z*: [M + Na]^+^ calculated for C_38_H_38_N_6_O_9_Na, 745.2598; found, 745.2590.

***tert*-Butyl *N*-(2-((((9H-fluoren-9-yl)methoxy)carbonyl)amino)ethyl)-*N*-(2-(5-(6-nitro-4-oxo-3,4-dihydroquinazolin-2-yl)uracil-1-yl)acetyl)glycinate (24)**


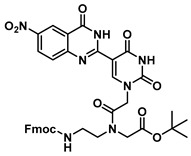


To 0.1 g (0.24 mmol) of **20** dissolved in 2 mL of DMF at 0 °C were added HOBt (23 mg, 0.19 mmol) and DCC (70 mg, 0.34 mmol), and the reaction was stirred until the formation of precipitation was observed. Then, 70 mg (0.15 mmol) of **6** as a free base with DMAP (1.8 mg, 0.015 mmol) were added, and the reaction continued for 3 h and was checked by TLC. The mixture was diluted with saturated aqueous NaHCO_3_, extracted with DCM, washed with brine, concentrated in vacuo and purified by column chromatography (1:1 to 4:1 ethyl acetate/hexanes) to yield **24** as a yellow solid (66 mg, 0.09 mmol, 60%). ^1^H NMR (400 MHz, DMSO-*d*_6_) δ 8.85–8.70 (m, 1H), 7.91–7.82 (m, 3H), 7.69 (dd, *J* = 10.0, 7.0 Hz, 2H), 7.47–7.18 (m, 5H), 4.01 (ddd, *J* = 15.7, 14.1, 5.9 Hz, 1H), 3.19 (d, *J* = 3.2 Hz, 4H), 2.90 (s, 1H), 2.75–2.56 (m, 7H), 1.42 (s, 9H). ^13^C{^1^H} NMR (101 MHz, Chloroform-*d*) δ 171.9, 146.0, 141.0, 128.7, 127.4, 127.2, 127.1, 126.9, 126.9, 125.2, 124.7, 121.0, 119.9, 119.8, 119.7, 107.7, 81.3, 57.3, 53.0, 51.4, 48.8, 47.9, 44.7, 33.9, 31.2, 28.1. HRMS (ESI/Q-TOF) *m*/*z*: [M + Na]^+^ calculated for C_37_H_35_N_7_O_10_Na, 760.2343; found, 760.2350.

***N*-(2-((((9H-fluoren-9-yl)methoxy)carbonyl)amino)ethyl)-*N*-(2-(5-(4-oxo-3,4-dihydroquinazolin-2-yl)uracil-1-yl)acetyl)glycine (25)**


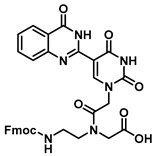


To 75 mg (0.10 mmol) of ester **22** were added TFA (1 mL) and triethylsilane (three drops). The reaction was stirred for 2 h at room temperature, then the excess acid was evaporated under a nitrogen stream, and the product was washed with hexane and ethyl ether to give acid **25** as a white solid (55 mg, 0.08 mmol, 80%). ^1^H NMR (400 MHz, DMSO-*d*_6_) δ 12.34 (s, 3H), 8.76 (s, 2H), 8.71 (s, 1H), 7.81 (dd, *J* = 7.5, 5.1 Hz, 6H), 7.68 (t, *J* = 6.8 Hz, 6H), 7.49 (ddd, *J* = 8.4, 5.2, 1.6 Hz, 8H), 7.38 (dt, *J* = 7.9, 2.1 Hz, 6H), 7.35–7.29 (m, 6H), 5.57 (s, 1H), 5.03 (s, 3H), 4.84 (s, 2H), 4.36 (d, *J* = 6.9 Hz, 4H), 4.23 (d, *J* = 7.0 Hz, 2H), 4.25 (t, *J* = 3.4 Hz, 4H), 4.02 (s, 3H), 1.25–1.03 (m, 9H).^13^C{^1^H} NMR (101 MHz, DMSO) δ 171.9, 170.5, 165.2, 164.6, 160.6, 157.7, 156.8, 156.6, 150.4, 150.1, 144.3, 141.2, 141.1, 134.3, 128.1, 128.0, 127.8, 127.5, 125.6, 125.6, 120.6, 106.4, 103.1, 49.4, 47.9, 47.2, 40.6, 40.4, 40.2, 39.9, 39.78, 39.6, 39.4, 36.7, 33.8, 25.8, 24.9. HRMS (ESI/Q-TOF) *m*/*z*: [M + Na]^+^ calculated for C_33_H_28_N_6_O_8_Na, 659.1866; found, 659.1859.

***N*-(2-((((9H-fluoren-9-yl)methoxy)carbonyl)amino)ethyl)-*N*-(2-(5-(6-methoxy-4-oxo-3,4-dihydroquinazolin-2-yl)uracil-1-yl)acetyl)glycine (26)**


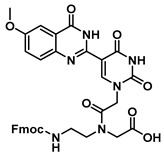


To 66 mg (0.09 mmol) of ester **23** were added TFA (1 mL) and triethylsilane (three drops). The reaction was stirred for 2 h at room temperature, then the excess acid was evaporated under a nitrogen stream, and the product was washed with hexane and ethyl ether to give acid **26** as a white solid (47 mg, 0.07 mmol, 75%). ^1^H NMR (400 MHz, DMSO-*d*_6_) δ 12.30 (s, 3H), 8.78 (s, 2H), 8.72 (s, 1H), 7.86 (dd, *J* = 7.5, 5.1 Hz, 6H), 7.68 (t, *J* = 6.8 Hz, 6H), 7.49 (ddd, *J* = 8.4, 5.2, 1.6 Hz, 8H), 7.38 (dt, *J* = 7.9, 2.1 Hz, 6H), 7.35–7.29 (m, 6H), 5.57 (s, 1H), 5.03 (s, 3H), 4.84 (s, 2H), 4.36 (d, *J* = 6.9 Hz, 4H), 4.29 (d, *J* = 7.0 Hz, 2H), 4.25 (t, *J* = 3.4 Hz, 4H), 4.02 (s, 3H), 3.88 (s, 3H), 1.27–1.03 (m, 9H).^13^C{^1^H} NMR (101 MHz, DMSO) δ 172.8, 170.8, 167.2, 164.6, 160.6, 157.7, 156.8, 156.6, 150.4, 150.1, 144.3, 141.2, 141.1, 134.3, 128.1, 128.0, 127.8, 127.5, 125.6, 125.6, 120.6, 106.4, 103.1, 56.1, 49.4, 47.9, 47.2, 40.6, 40.4, 40.2, 39.9, 39.78, 39.6, 39.4, 36.7, 33.8, 25.8, 24.9. HRMS (ESI/Q-TOF) *m*/*z*: [M + Na]^+^ calculated for C_34_H_36_N_6_O_9_Na, 695.2441; found, 695.2458.

***N*-(2-((((9H-fluoren-9-yl)methoxy)carbonyl)amino)ethyl)-*N*-(2-(5-(6-nitro-4-oxo-3,4-dihydroquinazolin-2-yl)uracil-1-yl)acetyl)glycine (27)**


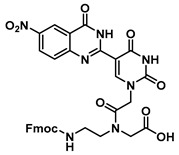


To ester **24** (58 mg, 0.07 mmol) were added TFA (1 mL) and triethylsilane (three drops). The reaction was stirred for 2 h at room temperature, then the excess acid was evaporated under a nitrogen stream, and the product was washed with hexane and ethyl ether to give acid **27** as a light yellow solid (47 mg, 0.05 mmol, 83%). ^1^H NMR (400 MHz, DMSO-*d*_6_) δ 12.57 (s, 1H), 9.05 (d, *J* = 3.4 Hz, 2H), 8.81 (s, 3H), 8.55 (d, *J* = 8.9 Hz, 4H), 8.49 (s, 2H), 7.91 (d, *J* = 7.2 Hz, 3H), 7.86 (t, *J* = 9.8 Hz, 5H), 7.76 (dd, *J* = 14.8, 8.1 Hz, 6H), 7.69 (s, 3H), 7.46–7.29 (m, 11H), 5.07 (s, 2H), 4.87 (s, 2H), 4.68 (s, 3H), 4.13 (s, 2H), 4.03 (s, 2H), 3.56 (s, 4H), 3.28 (s, 8H), 2.92 (d, *J* = 21.0 Hz, 11H). ^13^C{^1^H} NMR (101 MHz, DMSO-*d*_6_) δ 171.7, 169.7, 167.1, 163.5, 160.6, 157.8, 156.8, 156.6, 150.4, 150.1, 144.3, 141.2, 141.1, 134.3, 128.1, 128.0, 127.8, 127.5, 125.6, 125.6, 120.5, 106.4, 103.1, 49.4, 47.9, 47.2, 40.6, 40.4, 40.2, 39.9, 39.8, 39.6, 39.4, 36.7, 33.8, 25.8, 24.9. HRMS (ESI/Q-TOF) *m*/*z*: [M + Na]^+^ calculated for C_33_H_27_N_7_O_10_Na, 704.1717; found, 704.1739.

### 3.2. Oligomer Synthesis

PNA oligomers were synthesized using the ABI 433A peptide synthesizer, using manufacturer-supplied “Fastmoc” cycles. Oligomerization was carried out using newly synthesized quinazolinone heterocycle monomers and commercially available Fmoc-A(Bhoc)-AEG-OH, Fmoc-G(Bhoc)-AEG-OH, Fmoc-C(Bhoc)-AEG-OH and Fmoc-T(Bhoc)-AEG-OH (purchased from PolyOrg, Inc.), and *N*α-Fmoc-*N*ε-Boc-L-lysine (Chem-Impex International Inc.). FmocRAM-PS was used as a solid support resin, preloaded with L-lysine at 0.057 mmol/g. The synthesis was carried out on a 5.0 µmol scale. The synthesis cycle was only modified by using 20% 4-methylpiperidine in dimethylformamide for Fmoc deprotection [23]. Following automated synthesis, the resin was treated with a solution of 95% trifluoroacetic acid and 5% triethylsilane to cleave the oligomer from the resin and remove the protecting group from the nucleobases (Bhoc) and amino group (Boc). The solvent was then evaporated under a nitrogen stream and the resulting residue was washed twice with cold ether, dissolved in a solution of 0.05% trifluoroacetic acid in water and then purified by reverse-phase HPLC. Reverse-phase HPLC was performed on an Agilent MicrosorbMV 100-5 C18 250 × 4.6 mm column heated to 50 °C. The purified PNA oligomer was eluted using a gradient (water/0.1% trifluoroacetic acid to acetonitrile/0.1% trifluoroacetic acid) over 60 min.

### 3.3. Thermal Stability Analysis

The thermal stabilities (melting temperature, T_m_) of complexes were measured in solutions of 100 mM NaCl, 10 mM sodium hydrogen phosphate, 0.1 mM EDTA, pH = 7.0 with individual PNA strand concentrations of 2 µM. Absorbance at λ = 260 nm was measured at 0.5 °C intervals while the temperature was changed at a rate of 0.7 °C/min between 15 and 90 °C. T_m_ values were measured in triplicate and determined by the first derivative method applied through the manufacturer-supplied Varian WinUV Bio software.

### 3.4. Computational Studies

Structures were constructed in Spartan ’14 and ground state energy was minimized using a desktop computer at the Hartree-Foch 6-31+G* level. A tautomer search was conducted at the Hartree-Foch 6-31+G* level in order to confirm the lowest energy structures that represented the most favorable tautomeric forms.

## 4. Conclusions

A facile synthesis of C5-(quinazolinone)-uracil PNA monomers has been established that is compatible with standard Fmoc-based oligomerization chemistry. The modified nucleobases bearing quinazolinone moieties were characterized by their fluorescence quantum yield in nonpolar (THF) and polar solvents (EtOH, DMSO), and their responses to changes in viscosity and temperature are consistent with molecular rotor behavior. Single modifications incorporated into a PNA decamer resulted in the stabilization of hybrids formed with complementary DNA, as judged by the UV-vis measured T_m_ values, in the following order: **^Q^U**^(NO2)^ > **^Q^U**^(OMe)^ > **^Q^U**. Interestingly, this order is qualitatively reflected by the degree of fluorescence increase going from the single-stranded PNA to the PNA–DNA heteroduplex (**^Q^U**^(NO2)^ > **^Q^U**^(OMe)^ > **^Q^U**). The **^Q^U**^(OMe)^ and **^Q^U** labelled PNA oligomers dually report hybridization by an increase in fluorescence intensity and a large (~50 nm) hypsochromic shift. Thus, quinazolinone-based uracil analogs are good candidates for reporting binding events by showing emission “turn-on” properties upon hybridization to complementary DNA.

## Figures and Tables

**Table 1:** Optimization of the Catalytic Aerobic Oxidative Synthesis of Quinazolinone **^a^**.

Entry	Copper	Ligand	TEMPO	Solvent	Yield (%)
**1**	-	-	4-HO	toluene	32
**2**	CuBr	-	4-HO	DMF	22
**3**	CuCl	-	4-HO	DMF	43
**4**	CuCl	NEt_3_	4-HO	CH_3_CN	62
**5**	CuCl	NEt_3_	4-HO	CH_3_CN	80
**6**	CuCl	DMAP	4-HO	DMSO	86
**7**	CuCl	DABCO	4-HO	DMSO	12 ^b^
**8**	CuCl	DABCO	4-HO	DMSO	96

^a^ Anthranilamide (1.0 mmol) and 5-formyluracil derivative **2** (1.0 mmol) were dissolved in solvent (8 mL) in a 25 mL flask, and the mixture was stirred at 80 °C until the condensation completed (about 2 h). Then, copper salts (0.025 mmol), ligand (0.022 mmol) and 4-HO-TEMPO (0.012 mmol) were added to the reaction mixture, and stirring was continued at 90 °C under oxygen until the anthranilamide was consumed completely, as monitored by thin layer chromatography (TLC). ^b^Under nitrogen atmosphere.

**Table 2:** Photophysical properties ^a^ of dihydroquinazolinone compounds **7**–**11**.

Compound #	λ_max_ (nm), DMSO	$\varepsilon \;(\frac{L}{mol\cdot cm})$, DMSO	Φ ^c^_(EtOH)_	Φ _(DMSO)_	Φ _(THF)_
**7**	341	68,000	0.015 (412)	0.002 (428)	0.001 (441)
**8** ^b^	417	65,500	0.008 (417)	0.003 (436)	0.001 (452)
**9** ^b^	417	65,500	0.007 (417)	0.001 (436)	0.001 (452)
**10**	321	39,100	0.014 (386)	0.003 (412)	0.003 (455)
**11**	321	39,100	0.019 (386)	0.004 (412)	0.003 (455)

**^a^** The quantum yields were determined using emission wavelengths between 340 and 640 nm, with an excitation wavelength of 325 nm. **^b^** Excitation wavelength at 310 nm. ^c^ **λ_max_** emission, in nanometers, in parentheses.

**Table 3:** Photophysical properties ^a^ of quinazolinone compounds **12**–**16**.

Compound #	λ_max’_^b^ (nm)	$\varepsilon \;(\frac{L}{mol\cdot cm})$ ^c^	T (°C)	Φ ^c^_(EtOH)_	Φ _(DMSO)_	Φ _(THF)_	Φ _(Glycerol)_
**12**			60	0.006	0.002		0.031
	331	36,400	23	0.012 (401)	0.006 (415)	0.008 (440)	0.083 (422)
			15	0.038			
**13** ^d^			60	NA *	0.001		
	417	65,500	23		0.003 (434)	0.004 (453)	0.024 (394)
			15				
**14** ^d^			60	NA *	<0.001		
	417	65,500	23		0.001 (434)	0.003 (453)	0.018 (394)
			15				
**15**			60	0.006	0.007		0.059
	340	39,100	23	0.023 (432)	0.009 (437)	0.016 (458)	0.076 (452)
			15	0.041			
**16**			60	0.017	0.008		0.062
	340	39,100	23	0.035 (432)	0.011 (437)	0.019 (458)	0.106 (452)
			15	0.076			

**^a^** the quantum yields were determined using emission wavelengths between 340 and 640 nm, with an excitation wavelength of 330 nm; ^b^ λ_max_ = lowest energy absorbance band in UV/vis spectrum, in DMSO; **^c^** calculated based on absorption in DMSO; **^d^** excitation wavelength = 310 nm; * compound was not sufficiently soluble in ethanol.

**Table 4:** Thermal stabilities (T_m_) of double-stranded PNA (dsPNA) and heteroduplex complexes.

	vs. DNA *		vs. PNA ^†^	
Sequence (N→C)	T_m_ (°C)	ΔT_m_	T_m_ (°C)	ΔT_m_
H-Lys-GTAGATCACT-Lys-NH_2_	54.5		67.5	
H-GTAGA^Q^UCACT-Lys-NH_2_	55.5	+1.0	66.5	−1.0
H-GTAGA^Q^U^(NO2)^CACT-Lys-NH_2_	58.5	+4.0	72.5	+5.0
H-GTAGA^Q^U^(OMe)^CACT-Lys-NH_2_	56.5	+2.0	70.5	+3.0

PNA oligomers are listed from the pseudo 5′-terminus to the pseudo 3′-end. Lys = l-lysine, **^Q^U**^(NO2)^ = 6-nitroquinazolinone uracil, **^Q^U**^(OMe)^ = 6-methoxyquinazolinone uracil, **^Q^U** = quinazolinone uracil PNA residues. * The complementary DNA oligomer 5′-AGTGATCTAC-3′ was used. ^†^ The complementary PNA oligomer H-Lys-AGTGATCTAC-Lys-NH_2_ was used. Temperature-dependent UV spectra at 2 µM strand concentration, each in 100 mM sodium phosphate buffer, pH = 7.0.

## Supplementary Materials

Supplementary figures and tables, additional characterization information and NMR spectra.
