# Supplementary material for: Fluorescent Biaryl Uracils with C5-Dihydro- and Quinazolinone Heterocyclic Appendages in PNA
Source: Molecules. 2020 Apr 24;25(8):1995. doi: 10.3390/molecules25081995 (PMC7221758; doi:10.3390/molecules25081995)

Supporting Information

for

Fluorescent Biaryl Uracils with C5-Dihydro- and Quinazolinone Heterocyclic Appendages in PNA

Ali Heidari,[1,$] Arash Ghorbani-Choghamarani,[2,*] Maryam Hajjami,[2] and Robert H. E. Hudson[1,*]

[1] Department of Chemistry, The University of Western Ontario, London, ON, CANADA N6A 5B7; aheidar2@uwo.ca
[2] Department of Chemistry, Faculty of Science, Ilam University, 69315516, Ilam, Iran
[$] Current address: Institut für Physiologische Chemie und Pathobiochemie, Universitätsklinikum Münster, Waldeyerstr. 15, 48149 Münster, Germany
* Correspondence: rhhudson@uwo.ca; (Tel: +1-519-661-2111 Fax: +1-519-661-3022)
* Correspondence: a.ghorbani@ilam.ac.ir

Contents

General synthetic procedures S2

Quantum yield determination S3

Table S1. Observed high-resolution mass of synthesized PNA oligomers S4

Oligomer synthesis S4

RP-HPLC conditions and chromatograms S5

Table S2. Calculated quantum yield values for reference standards in different solvents and temperatures S11

NMR Spectra S12

General synthetic procedures

All chemicals were obtained from commercial sources and were of ACS reagent grade or higher and were used without further purification. Anhydrous and HPLC-grade solvents for PNA synthesis and chromatography were purchased from Caledon Laboratories. All other solvents were dried by passing through activated alumina columns. In all cases, sodium sulfate was used as the drying agent and solvent was removed by reduced pressure with Buchi Rotavapor. Thin-layer chromatography was performed on Silicycle Silica Gel TLC F-254 plates. Unless otherwise specified the R_f values are reported in the solvent system the reaction was monitored in. Flash chromatography was performed with Silicycle SiliaFlash® F60 230-400 mesh silica. All chemical shifts are reported in parts per million (δ), from tetramethylsilane (0 ppm), and are referenced to the residual proton in the respective solvent: $CDCl_3$ (7.26 ppm), DMSO-d_6 (2.49 ppm), methanol-d_6 (3.31 ppm) for ^{1}H NMR and $CDCl_3$ (77.0 ppm) and DMSO-d_6 (39.5 ppm) and methanol-d_6 (49.0 ppm) for ^{13}C NMR. Multiplicities are described as s (singlet), d (doublet), t (triplet), q (quartet), m (multiplet) and br s (broad singlet). Coupling constants (*J*) are reported in Hertz (Hz). Spectra were obtained on Bruker-400 and INOVA-400 and INOVA-600 instruments. The ^{1}H NMR and ^{13}C NMR for PNA monomers performed in $CDCl_3$ show the presence of rotamers. High-resolution mass spectra (HRMS) were obtained using electrospray ionization (ESI).

Quantum yield determination

Fluorescence quantum yields (Φ_F) of the quinazoline based monomer was determined using a Photon Technologies International Quanta Master 7/2005 spectrophotometer by the relative method using 9-10 diphenylanthracene ($\Phi_{EtOH} = 0.95$) [1] and tryptophan ($\Phi_{water, pH\ 7.2} = 0.14$) [2] as the reference standard in room temperature (**Table S2**). The quantum yields were determined using the integrated fluorescence intensity and an average of five emission scans for each compound and as a triplicate for each calculation. The quantum yield of the unknown $\Phi_{(x)}$ can be calculated by the following equation:

$$\Phi s = \left(\frac{Is}{Iref}\right) . \left(\frac{Abs\ ref}{Abs\ s}\right) . \left(\frac{\eta^2\ s}{\eta^2\ ref}\right) . \Phi\ ref$$

Where **Φ(ref)** is the quantum yield of the standard, **Abs** is the absorbance at the excitation wavelength, **I** is the integrated area in the emission curve, the subscripts **s** and **ref** refer to unknown and standard respectively and **η** is the refractive index of the solvent. By measuring a series of diluted solutions with various absorbance readings the following equation may be used:

$$\Phi s = \left(\frac{Grad\ s}{Grad\ ref}\right) . \left(\frac{\eta^2\ s}{\eta^2\ ref}\right) . \Phi\ ref$$

Where Grad is the gradient from the plot of the integrated area in the emission curve versus absorbance at the excitation wavelength.

Table S1. Observed high-resolution mass of synthesized PNA oligomers

Sequence[a] (N → C)	Molecular formula	Calculated $[M+Na]^+$	Observed $[M+Na]^+$
**H-Lys-AGTGATCTAC-Lys-NH$_2$**	$C_{120}H_{159}N_{61}O_{33}Na$	3005.2536	3005.2929
**H-Lys-GTAGATCACT-Lys-NH$_2$**	$C_{120}H_{159}N_{61}O_{33}Na$	3005.2536	3005.2551
**H-GTAGA^{Q}UCACT-Lys-NH$_2$**	$C_{121}H_{154}N_{64}O_{32}Na_2$	1530.6093 [b]	1530.6122
**H-GTAGA^{Q}U$^{(NO2)}$CACT-Lys-NH$_2$**	$C_{121}H_{153}N_{65}O_{34}Na_2$	1553.1018 [b]	1553.1120
**H-GTAGA^{Q}U$^{(OMe)}$CACT-Lys-NH$_2$**	$C_{122}H_{156}N_{64}O_{33}Na_2$	1545.6145 [b]	1545.6220

[a] PNA sequences possess a free N-terminal amino group and C-terminal amide

[b] Oligomer calculated and observed as the dicationic $[M+2Na]^{2+}$

Oligomer synthesis

PNA oligomers were synthesized using the ABI 433A peptide synthesizer manufactured by Perkin Elmer Applied Biosystems. Oligomerization was carried out using newly synthesized ^{s}U monomer, commercially available PNA monomers: Fmoc-A(Bhoc)-AEG-OH, Fmoc-G(Bhoc)-AEG-OH, and Fmoc-C(Bhoc)-AEG-OH, Fmoc-T(Bhoc)-AEG-OH (purchased from PolyOrg, Inc.), and *N*α-Fmoc-*N*ε-Boc-L-lysine (purchased from Chem-Impex Int'l Inc.), using standard Fmoc-based solid-phase synthesis protocol. Fmoc-RAM-PS was used as a solid support resin preloaded with lysine at 0.057 mmol/g. The synthesis was carried out on a 5.0 µmol scale. Monomers were prepared with 25 µmol dissolved in 110 µL. Solutions of 0.4 M diisopropylethylamine in *N*-methyl-2-pyrrolidone (NMP) and 0.19 M HBTU in NMP were prepared for monomer coupling. Fmoc deprotection was performed using a solution of 20% 4-methylpiperidine in dimethylformamide. Unreacted terminal amino groups were capped with acetic anhydride, using a solution of 1:25:25 acetic anhydride: pyridine: NMP. Following automated synthesis, the resin was treated with a solution of 95 % trifluoroacetic acid and 5% triethylsilane to cleave the oligomer from the resin and remove the protecting group from the nucleobases (Bhoc) and amino group (Boc). The solvent was then evaporated under a nitrogen stream, the resulting residue was washed twice with cold ether, dissolved in a solution of 0.05%

trifluoroacetic acid in water then purified by reverse-phase HPLC. Reverse-phase HPLC was performed on an Agilent Microsorb-MV 100-5 C18 250 × 4.6 mm column heated to 50 °C. The purified PNA oligomer was eluted using a gradient (water/0.1 % trifluoroacetic acid to acetonitrile/0.1 % trifluoroacetic acid).

RP-HPLC conditions and chromatograms

For PNA oligomers: 0-50% B in 50 min and 50-100% B in 10 min (Mobile phase A: H_2O containing 0.1% TFA. Mobile phase B: acetonitrile containing 0.1% TFA) (unless otherwise stated). For DNA oligomers: 0-20% A in 20 min and 20-100% A in 10 min (Mobile phase A: Acetonitrile. Mobile phase B: 0.1 M TEAA buffer). The flow rate was 1 mL/min.

RP-HPLC chromatograms of the crude (a) and pure (b) PNA oligomer **H-Lys-AGTGATCTAC-Lys-NH$_2$**

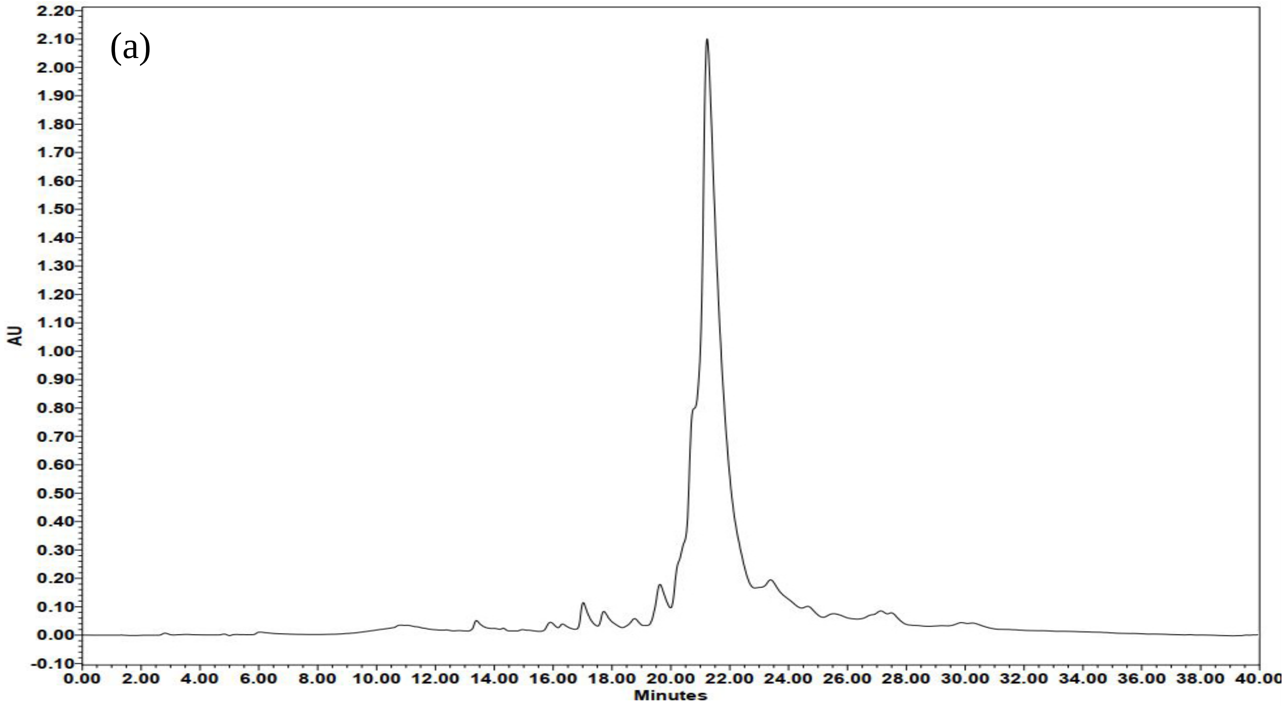

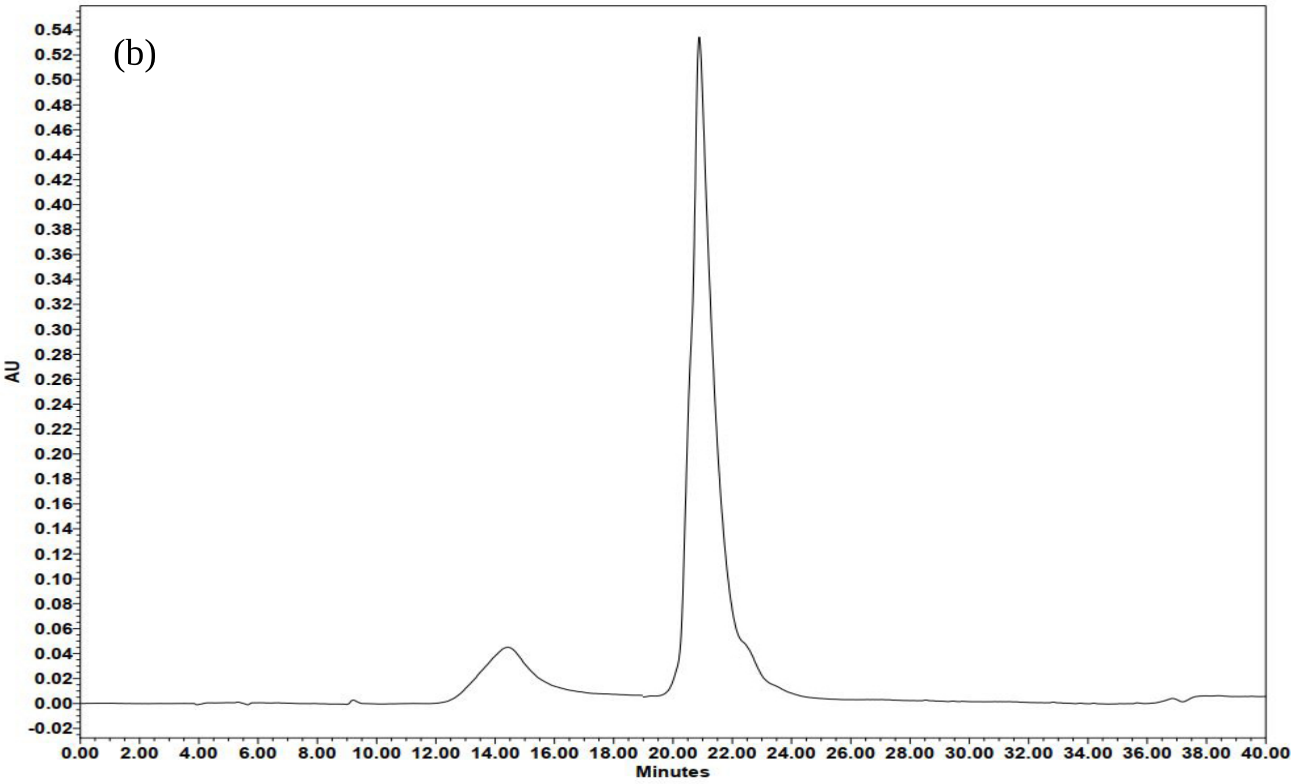

RP-HPLC chromatograms of the crude (a) and pure (b) PNA oligomer **H-GTAGA[Q]UCACT-Lys-NH$_2$**

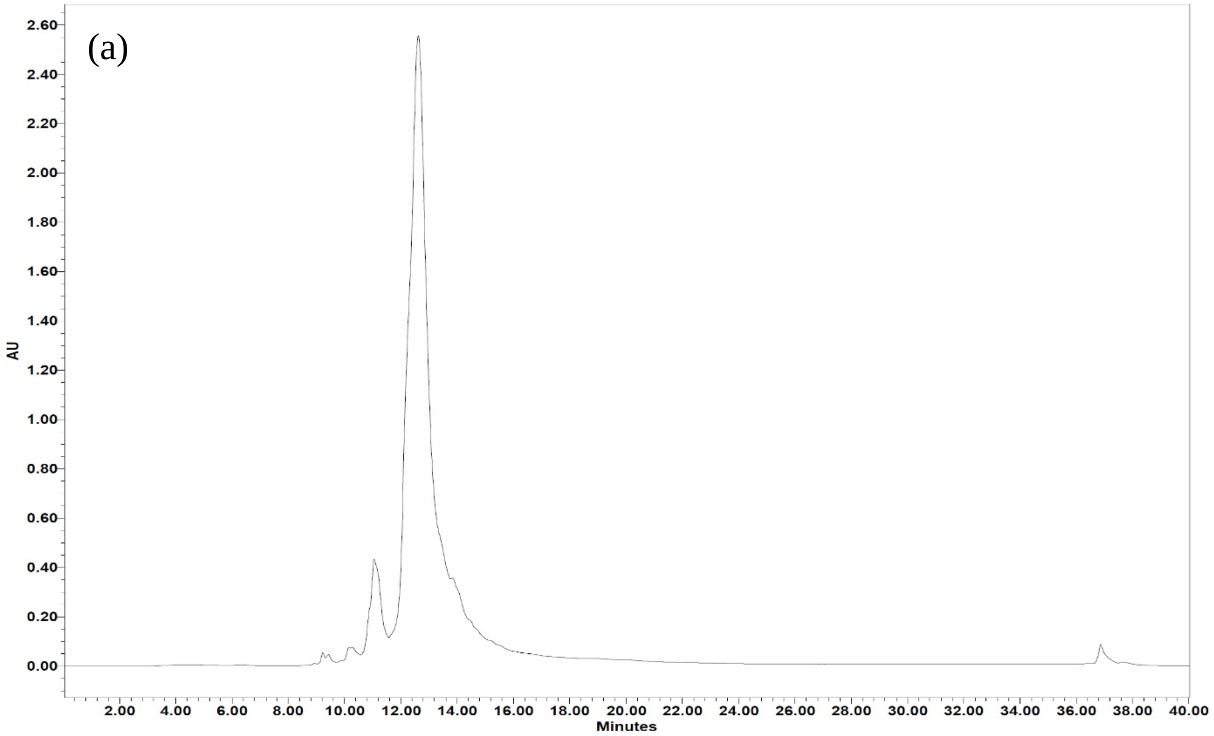

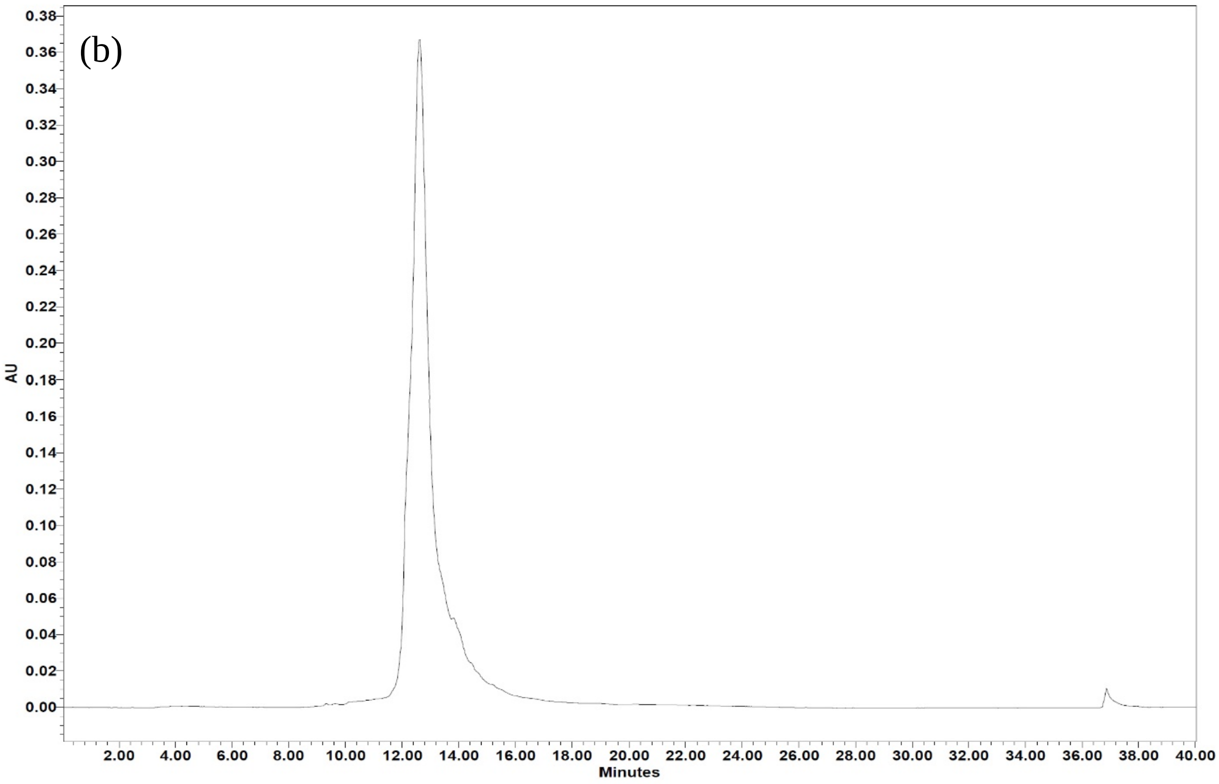

RP-HPLC chromatograms of the crude (a) and pure (b) PNA oligomer **H-GTAGA^{Q}U$^{(OMe)}$CACT-Lys-NH$_2$**

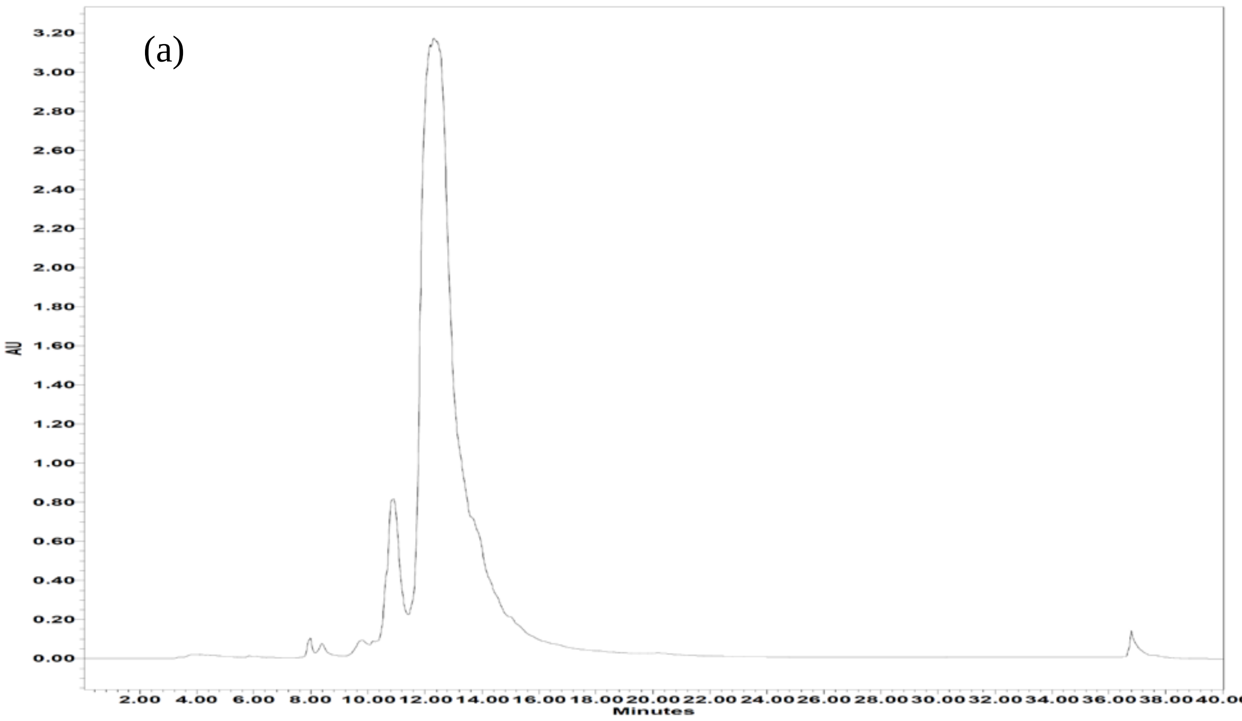

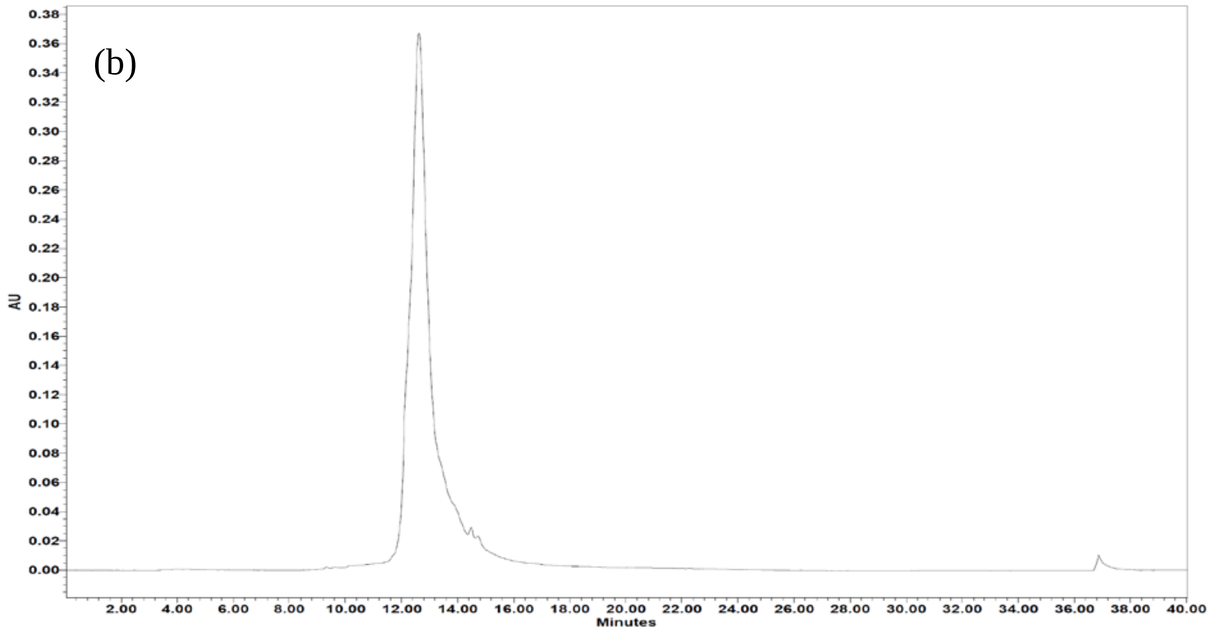

RP-HPLC chromatograms of the crude (a) and pure (b) PNA oligomer **H-GTAGA^{Q}U$^{(NO2)}$CACT-Lys-NH$_2$**

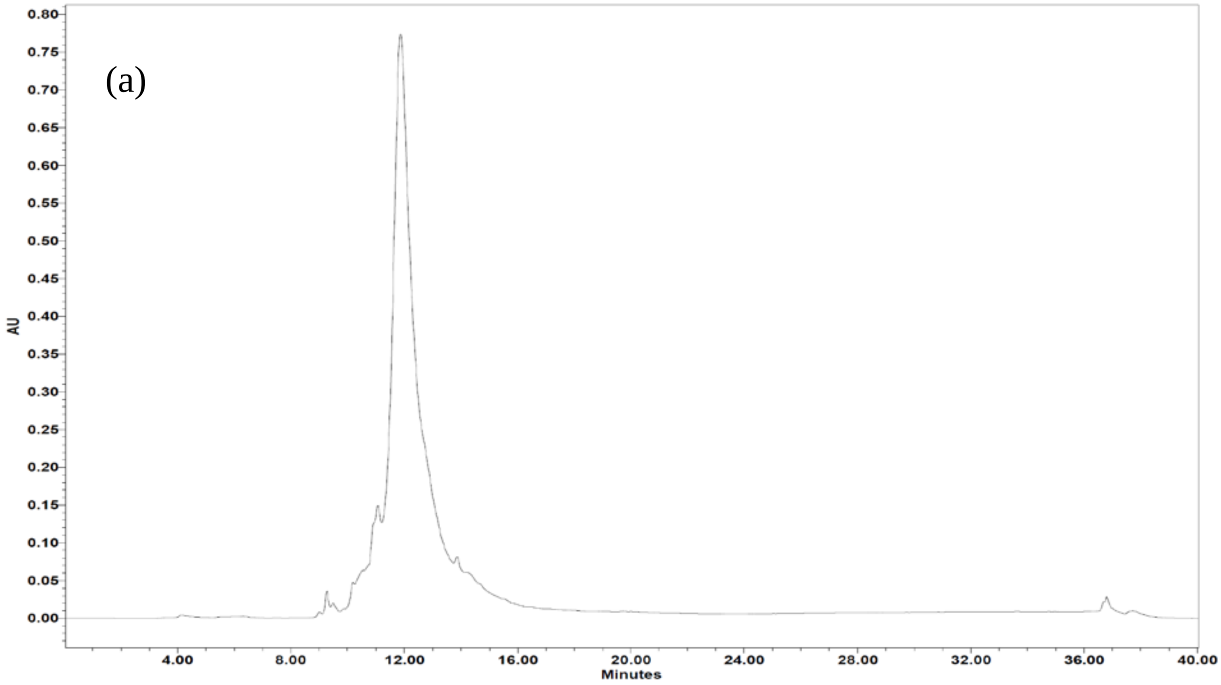

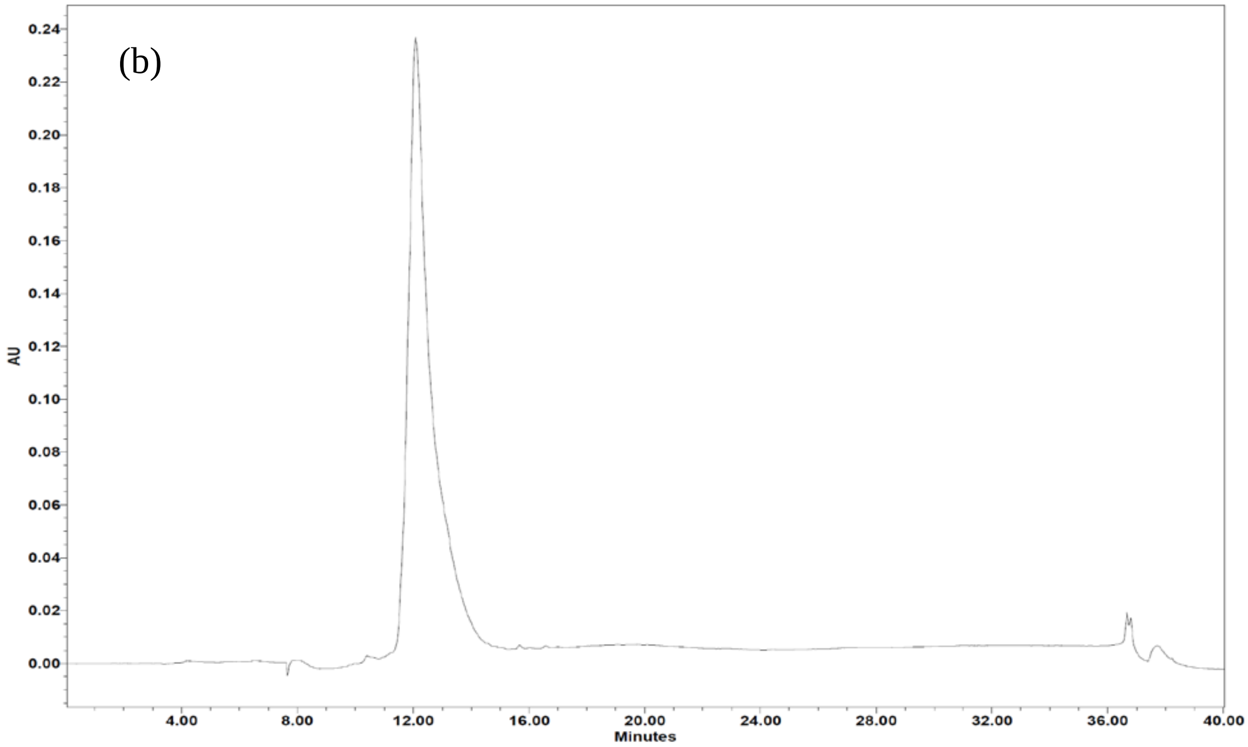

RP-HPLC chromatograms of the (a) crude (DMT-on) and (b) pure (DMT-off) DNA oligomer **5' AGTGATCTACCT 3'**

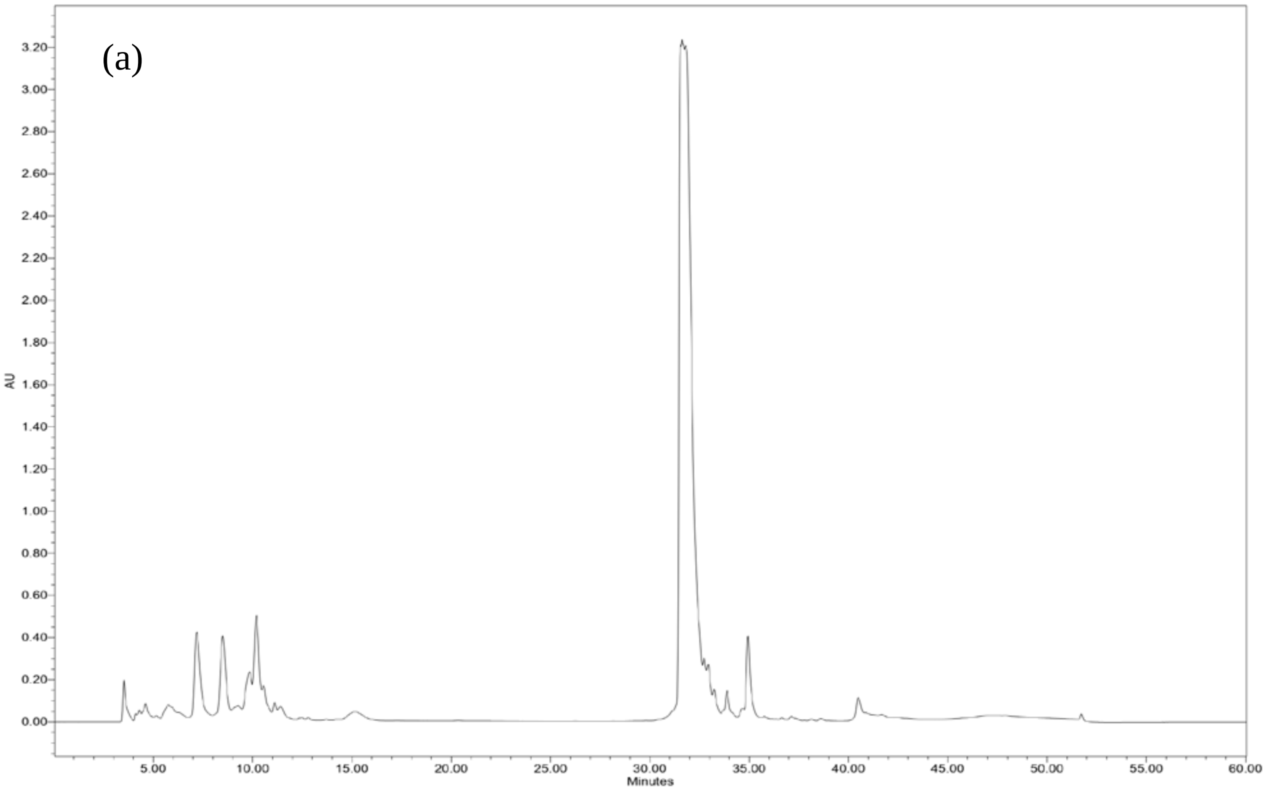

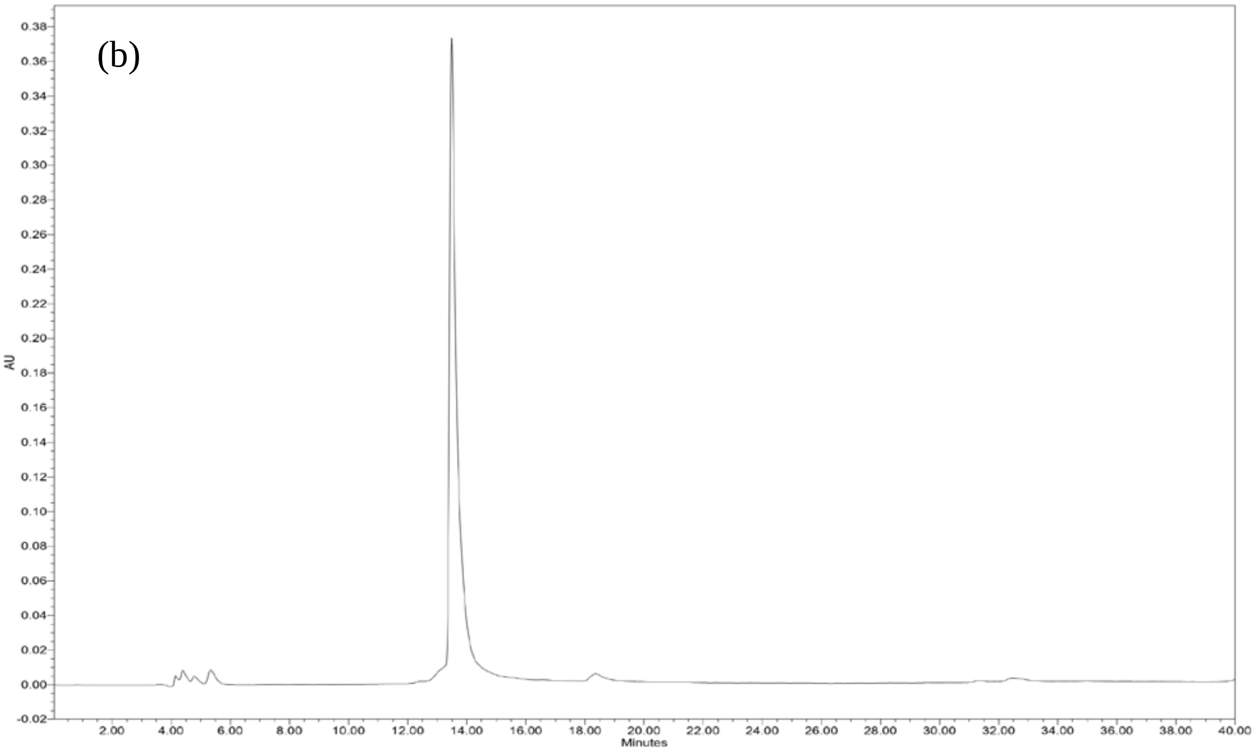

Table S2. Calculated quantum yield values for reference standards in different solvents and temperatures

Reference Fluorophore	Φ (EtOH)	Φ (DMSO)	Φ (THF)	Φ (Glycerol)	Φ (water, pH 7.2)	T (°C)
9,10-diphenylanthracene	0.95[a]	0.27	0.42	0.89		23
	0.79	0.24		0.81		60
	0.95					15
Tryptophan		0.27	0.35	0.31	0.14[a]	23
		0.28				60
						15

[a] Values extracted from literature.[1][2]

NMR Spectra

^{1}H NMR spectrum of 5-Formyluracil (1)

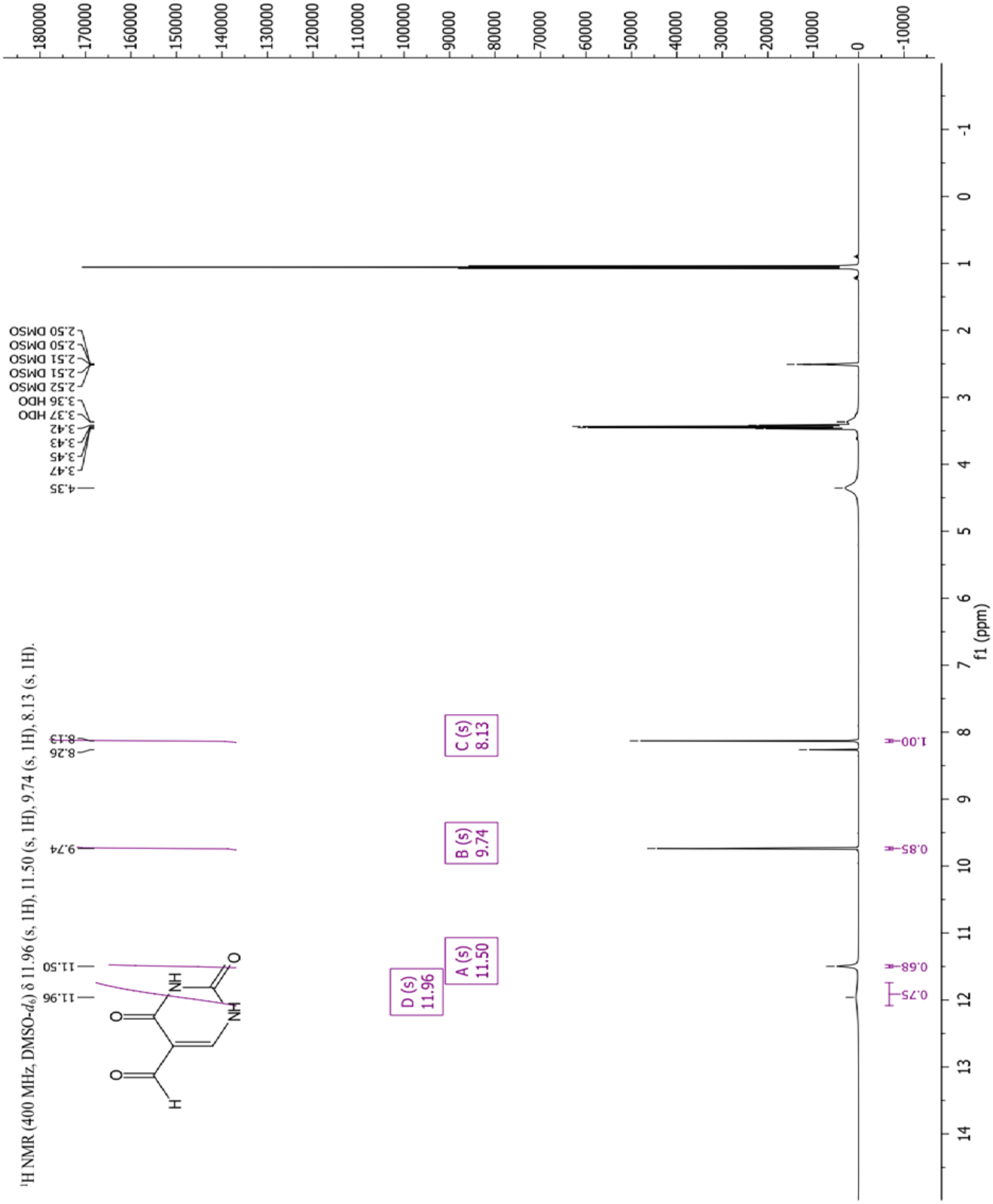

^{13}C NMR spectrum of 5-Formyluracil (1)

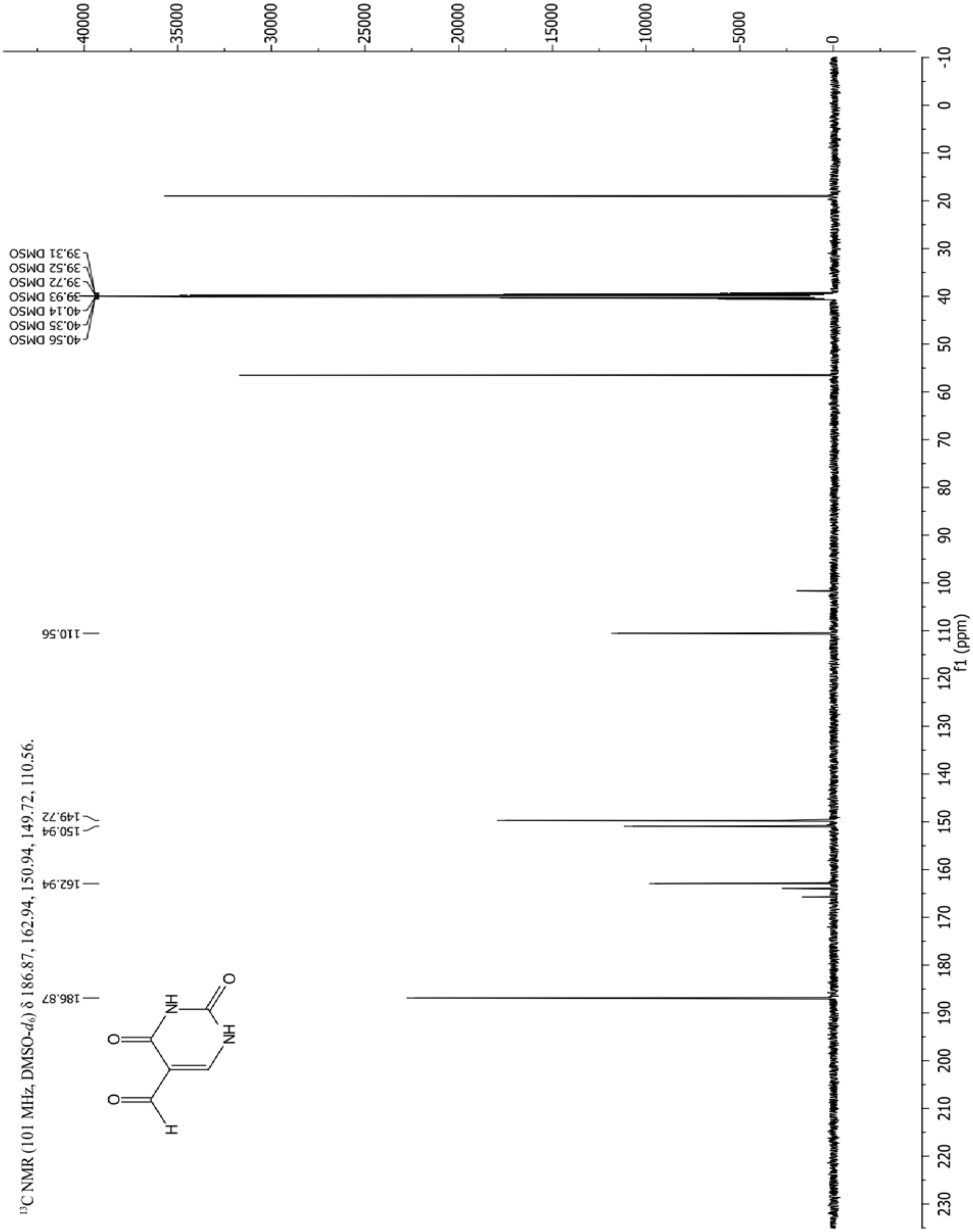

^{1}H NMR spectrum of *tert*-Butyl (uracil-5-formaldehyde-1-yl) acetate (2)

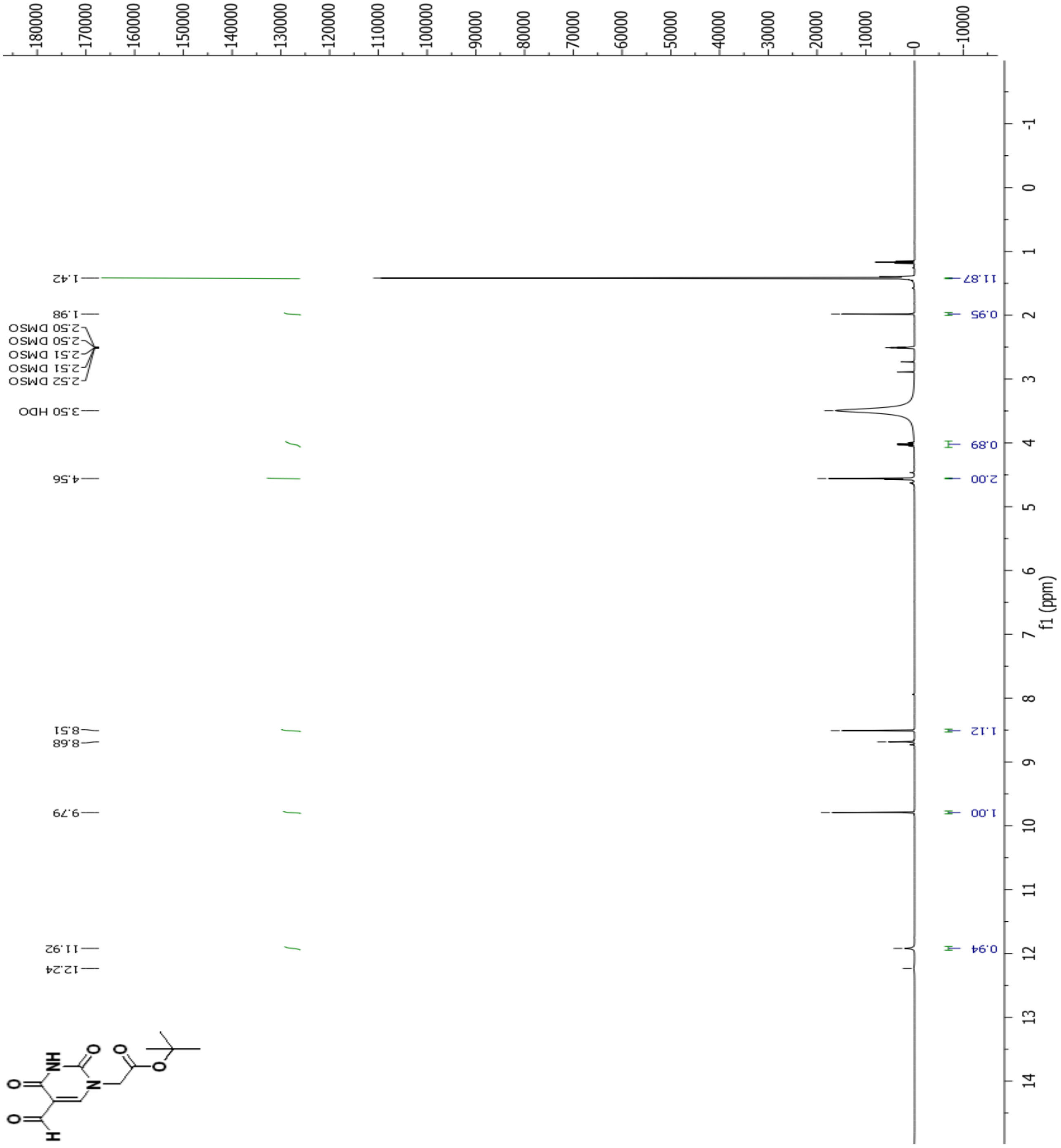

^{13}C NMR spectrum of *tert*-Butyl (uracil-5-formaldehyde-1-yl) acetate (2)

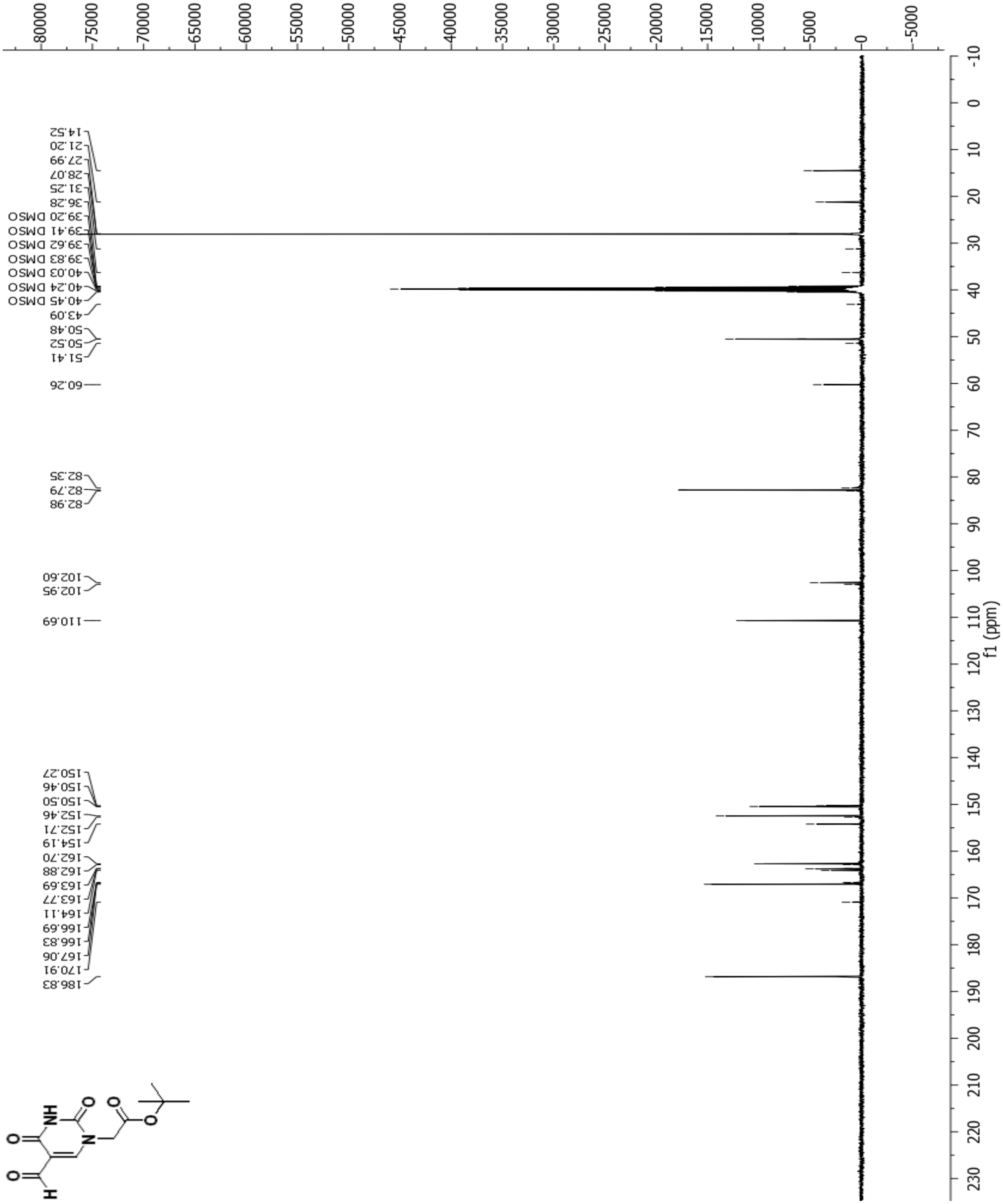

^{1}H NMR spectrum of 2-Amino-5-methoxybenzamide (3)

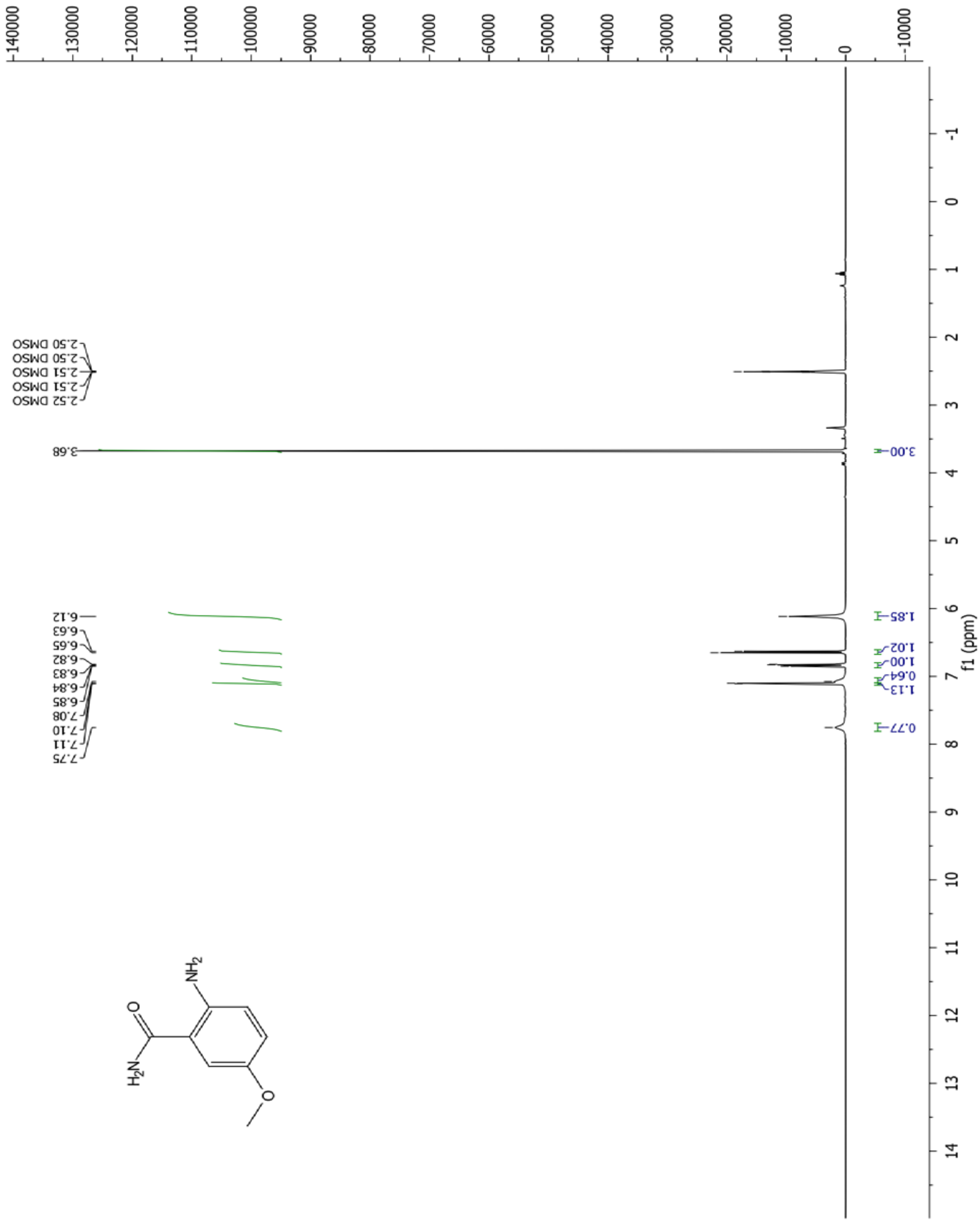

^{13}C NMR spectrum of 2-Amino-5-methoxybenzamide (3)

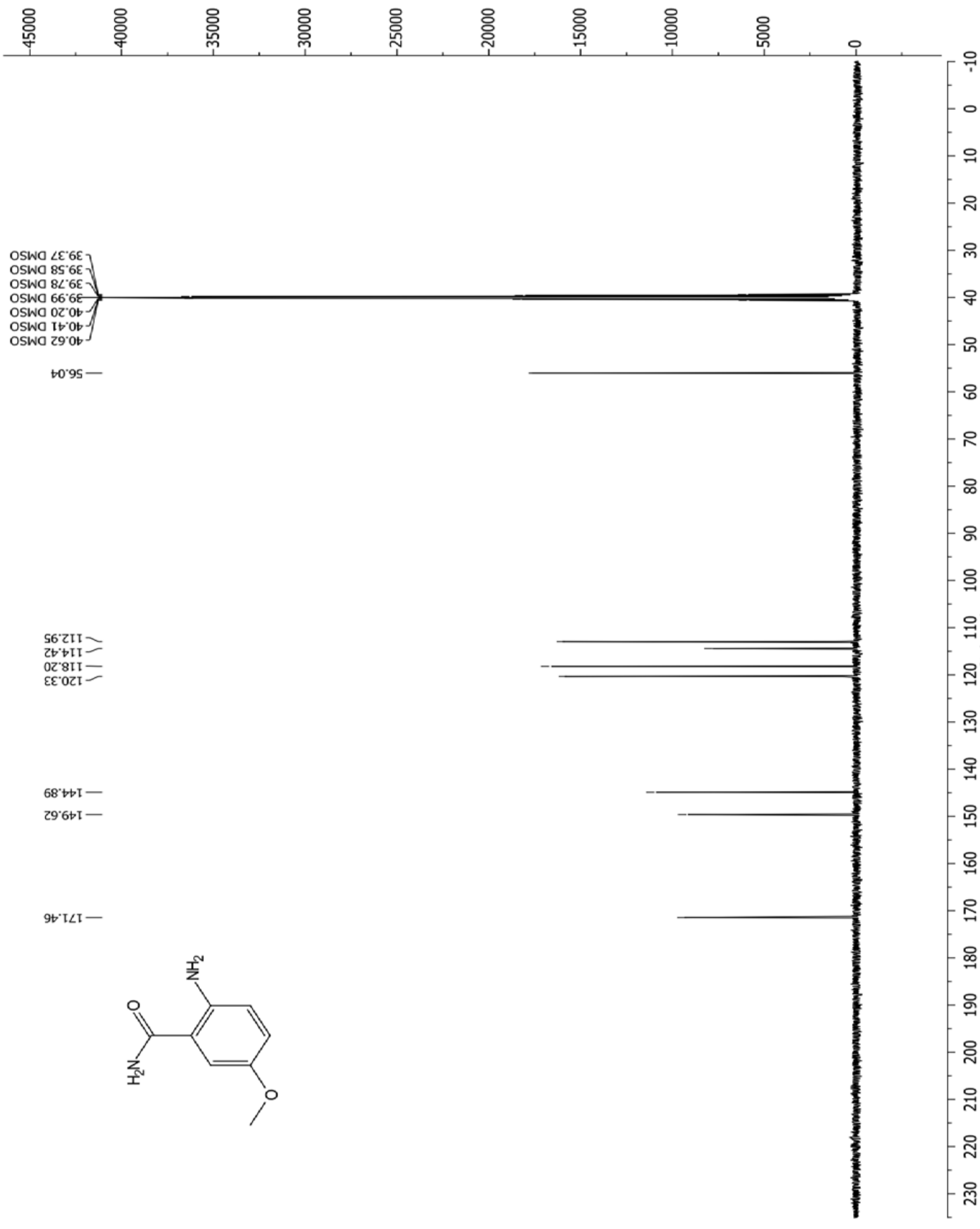

^{1}H NMR spectrum of 2-Amino-5-nitrobenzamide (4)

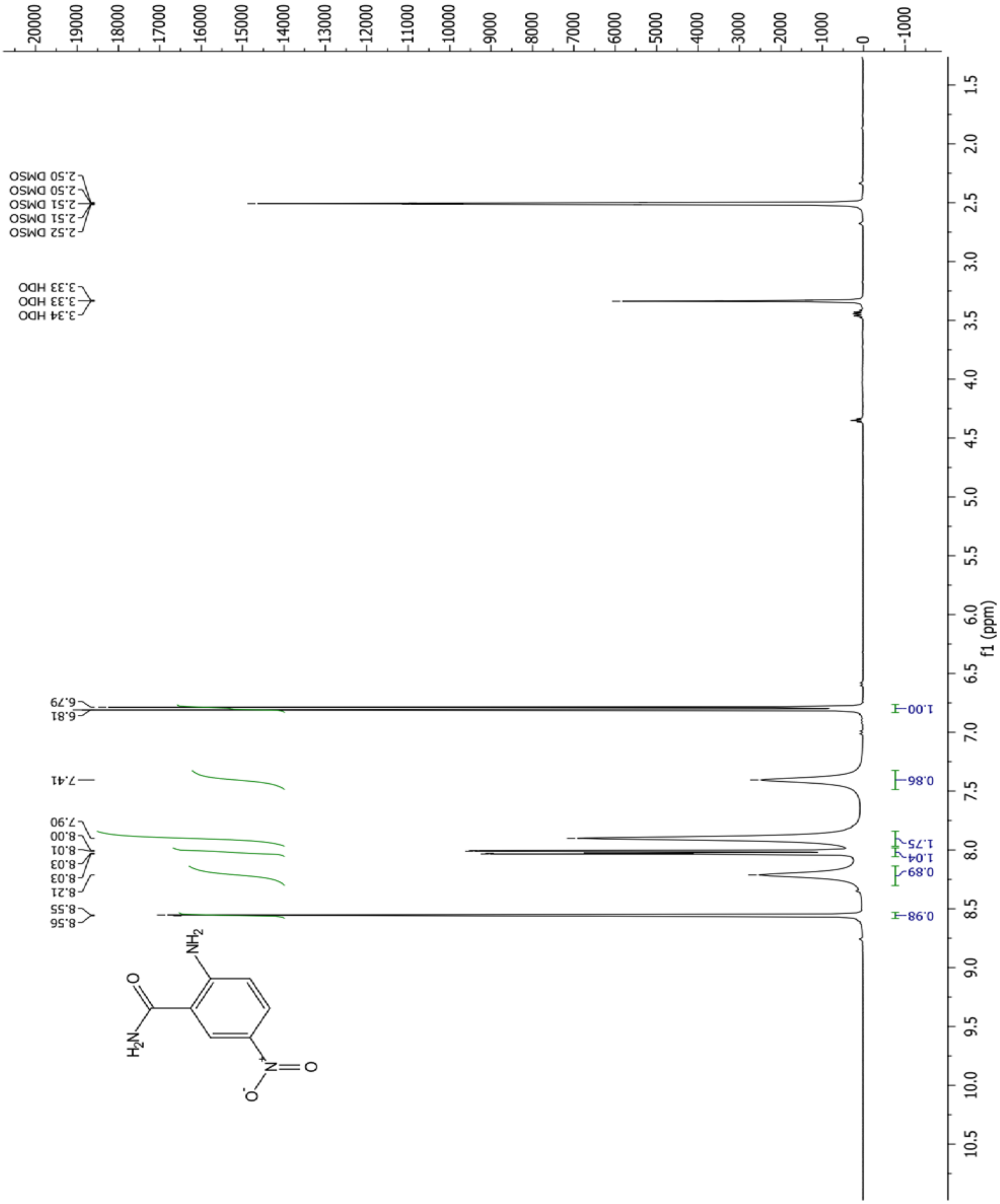

^{13}C NMR spectrum of 2-Amino-5-nitrobenzamide (4)

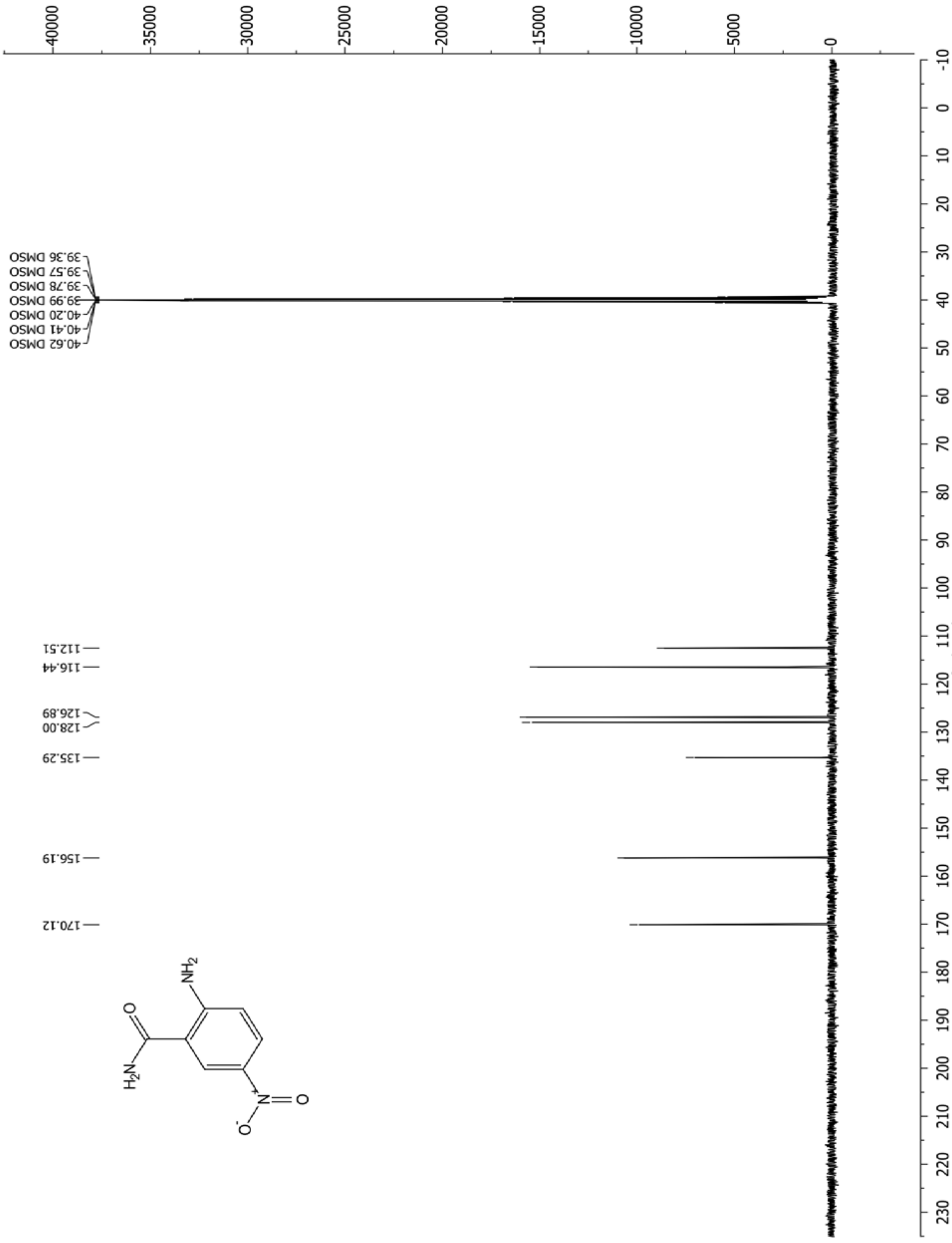

^{1}H NMR spectrum of 2-Amino-4-methoxybenzamide (5)

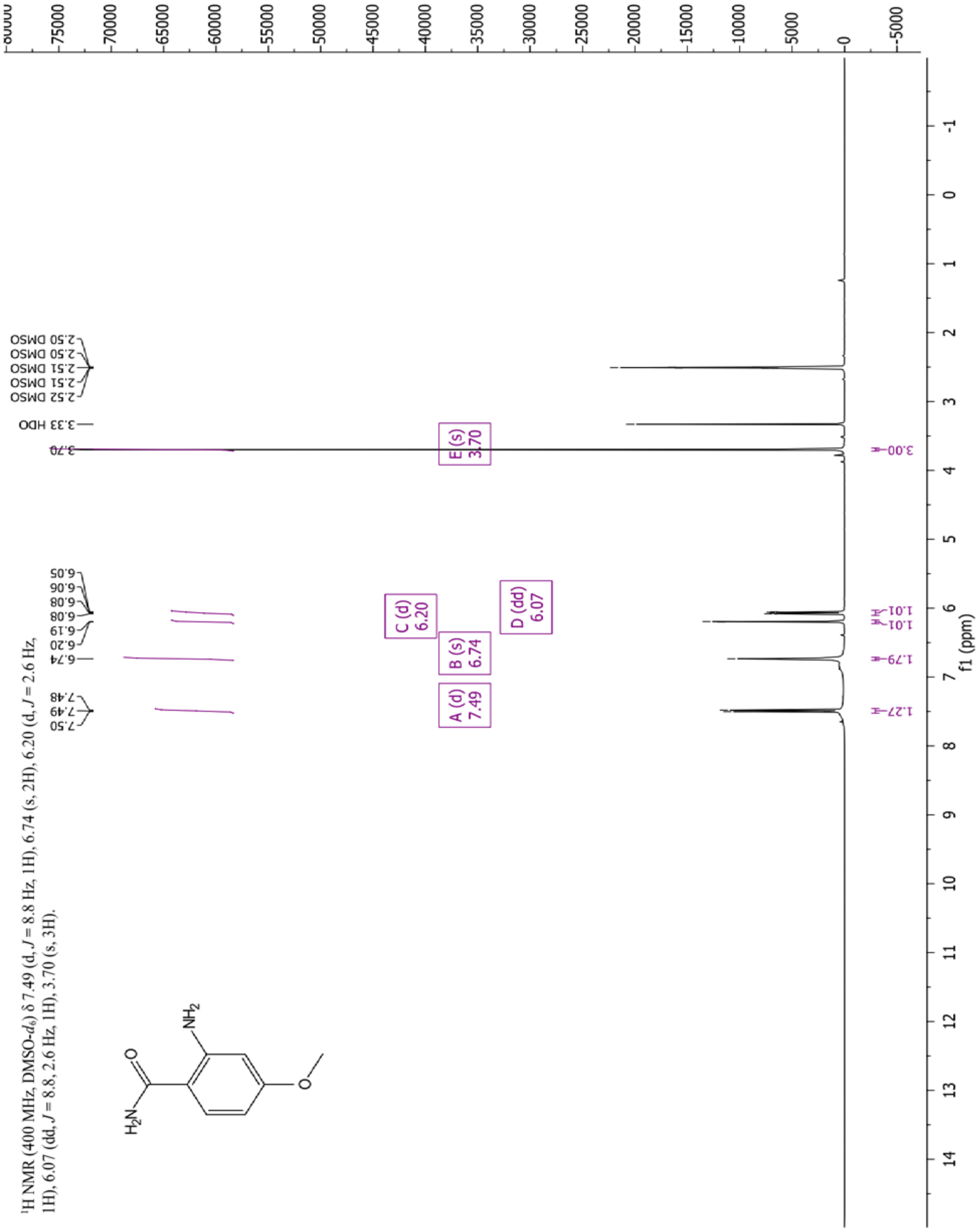

^{1}H NMR spectrum of 2-Amino-4-nitrobenzamide (6)

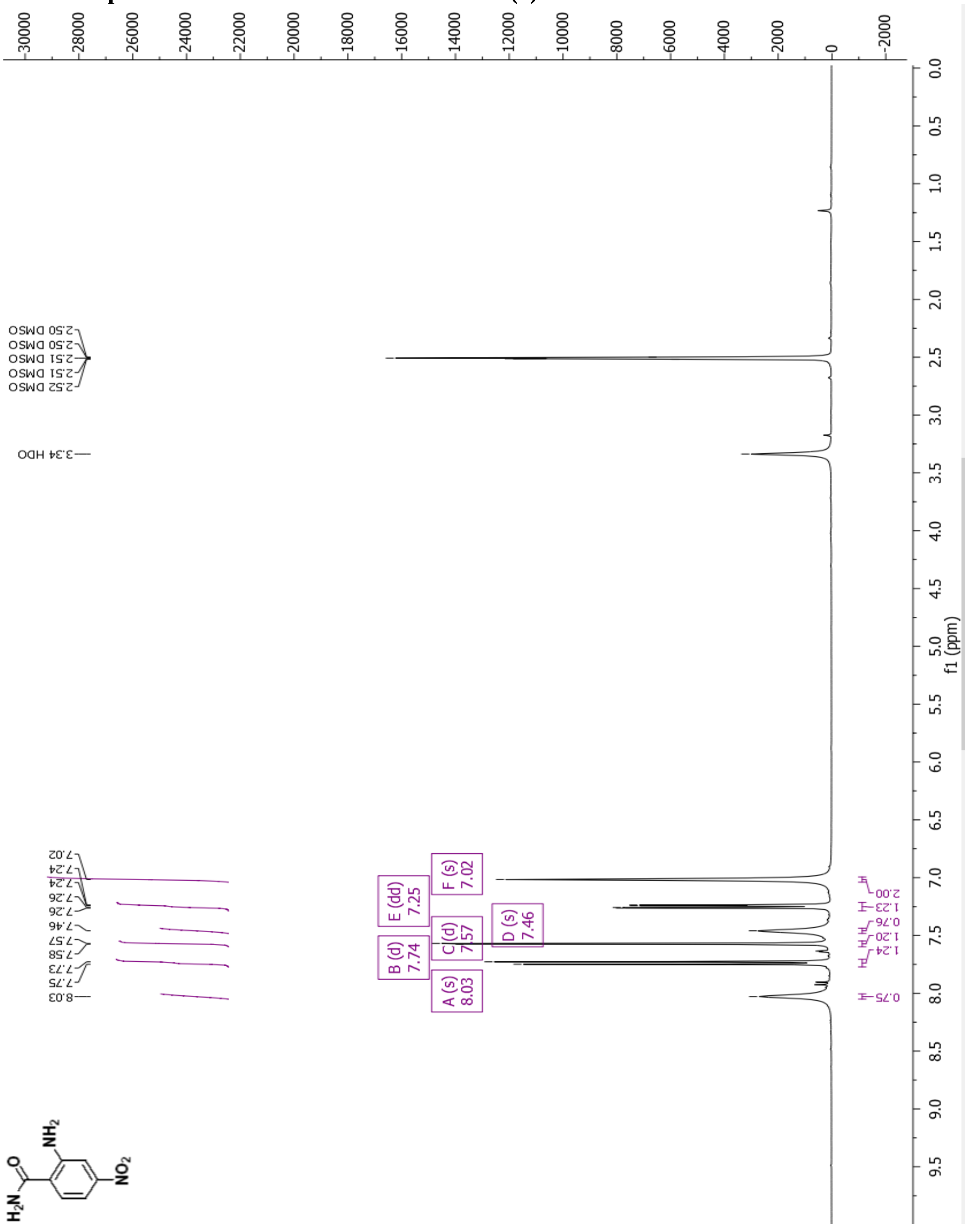

^{1}**H NMR spectrum of *tert*-Butyl 2-(5-(4-oxo-1,2,3,4-tetrahydroquinazolin-2-yl)uracil-1-yl)acetate (7)**

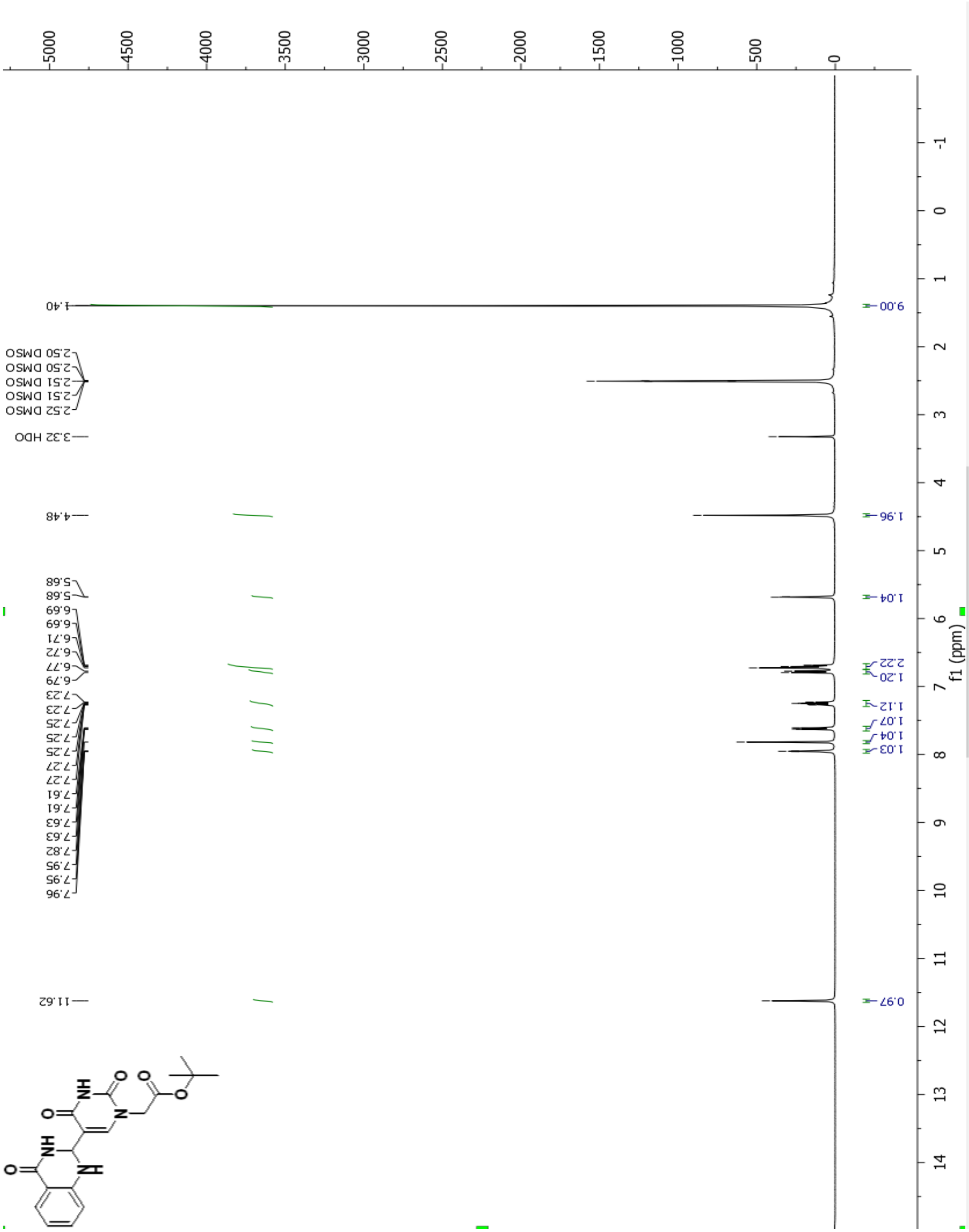

^{13}C NMR spectrum of *tert*-Butyl 2-(5-(4-oxo-1,2,3,4-tetrahydroquinazolin-2-yl)uracil-1-yl)acetate (7)

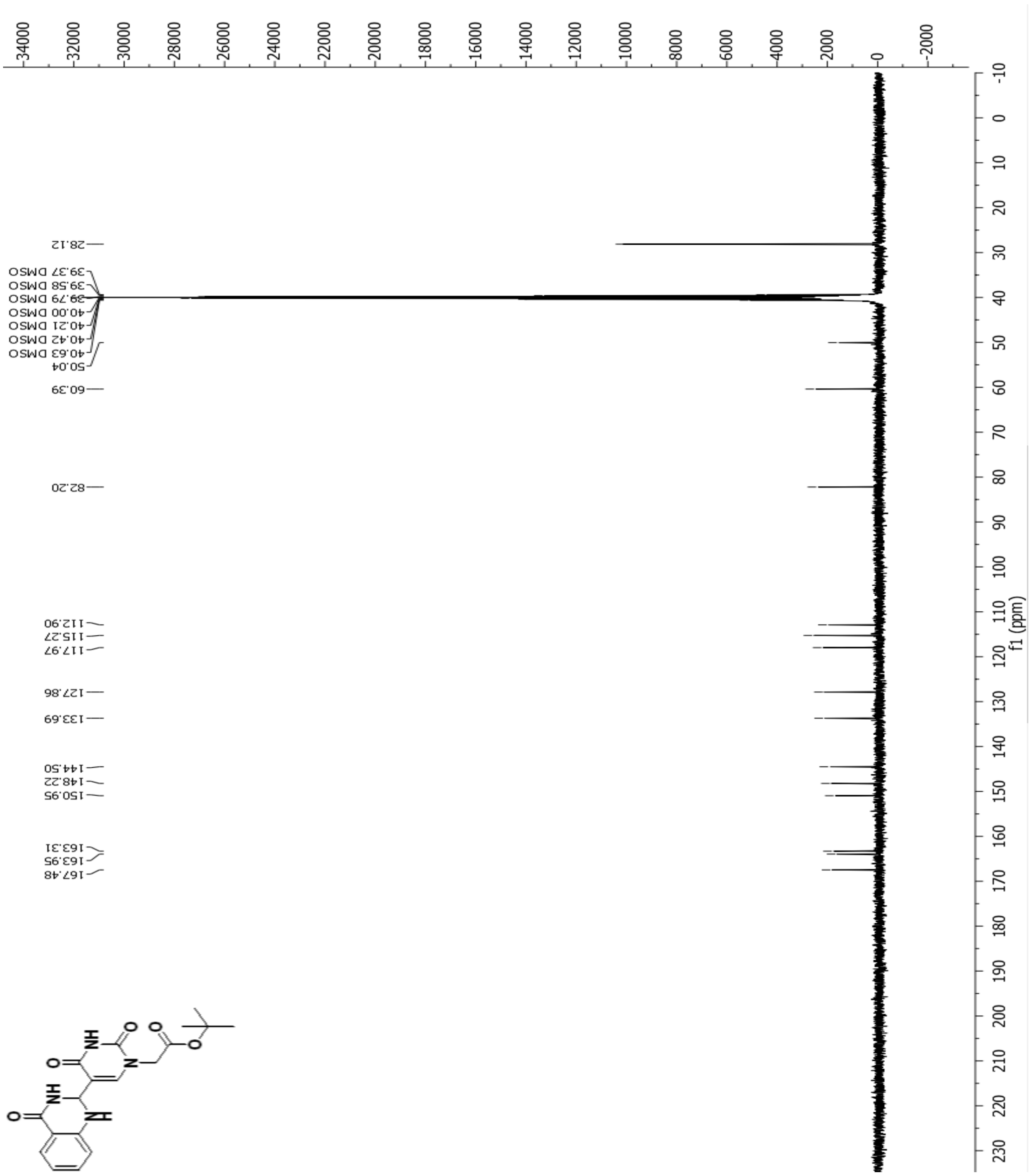

^{1}H NMR spectrum of *tert*-Butyl 2-(5-(6-nitro-4-oxo-1,2,3,4-tetrahydroquinazolin-2-yl)uracil-1-yl) acetate (8)

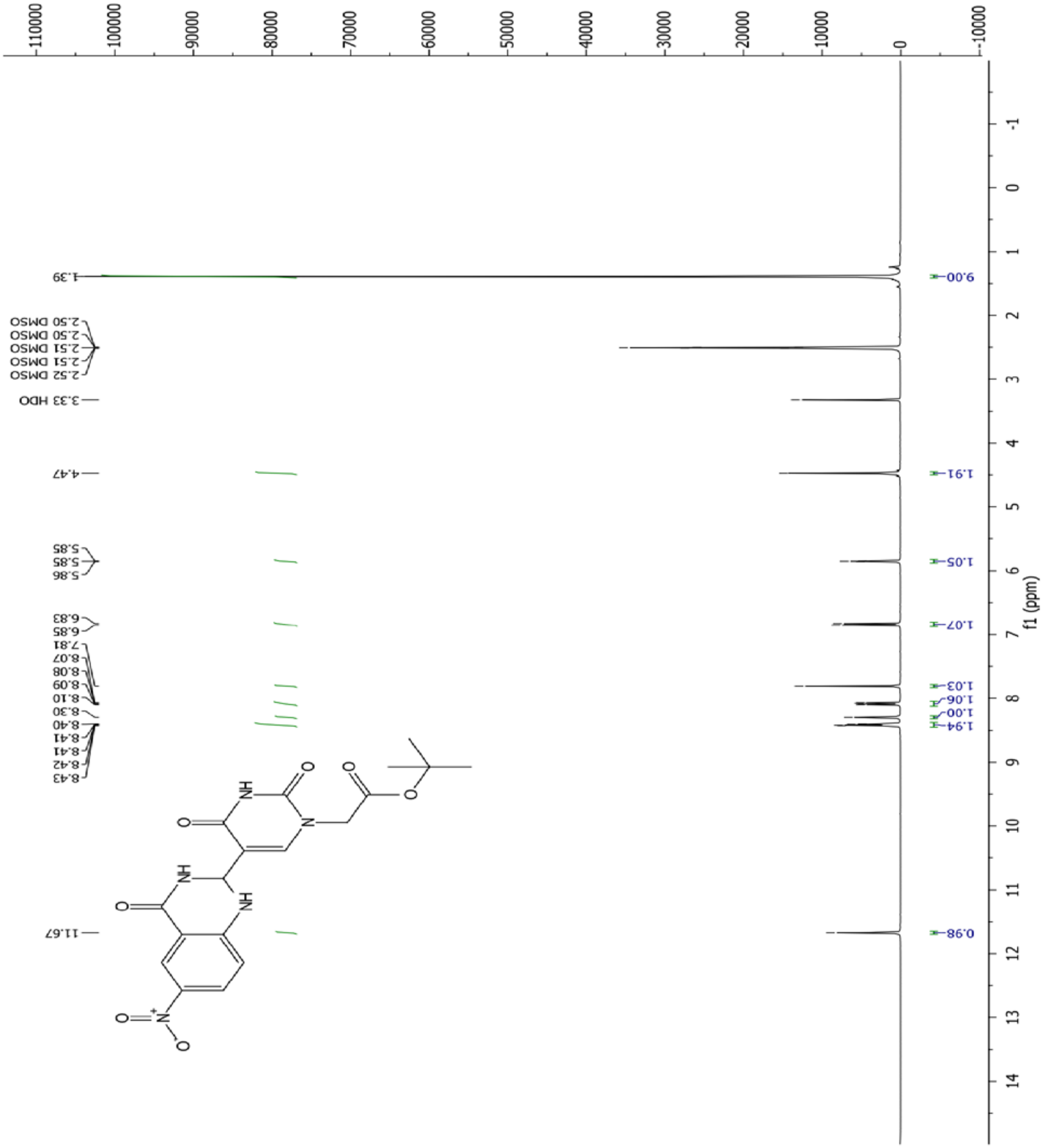

^{13}C NMR spectrum of *tert*-Butyl 2-(5-(6-nitro-4-oxo-1,2,3,4-tetrahydroquinazolin-2-yl)uracil-1-yl) acetate (8)

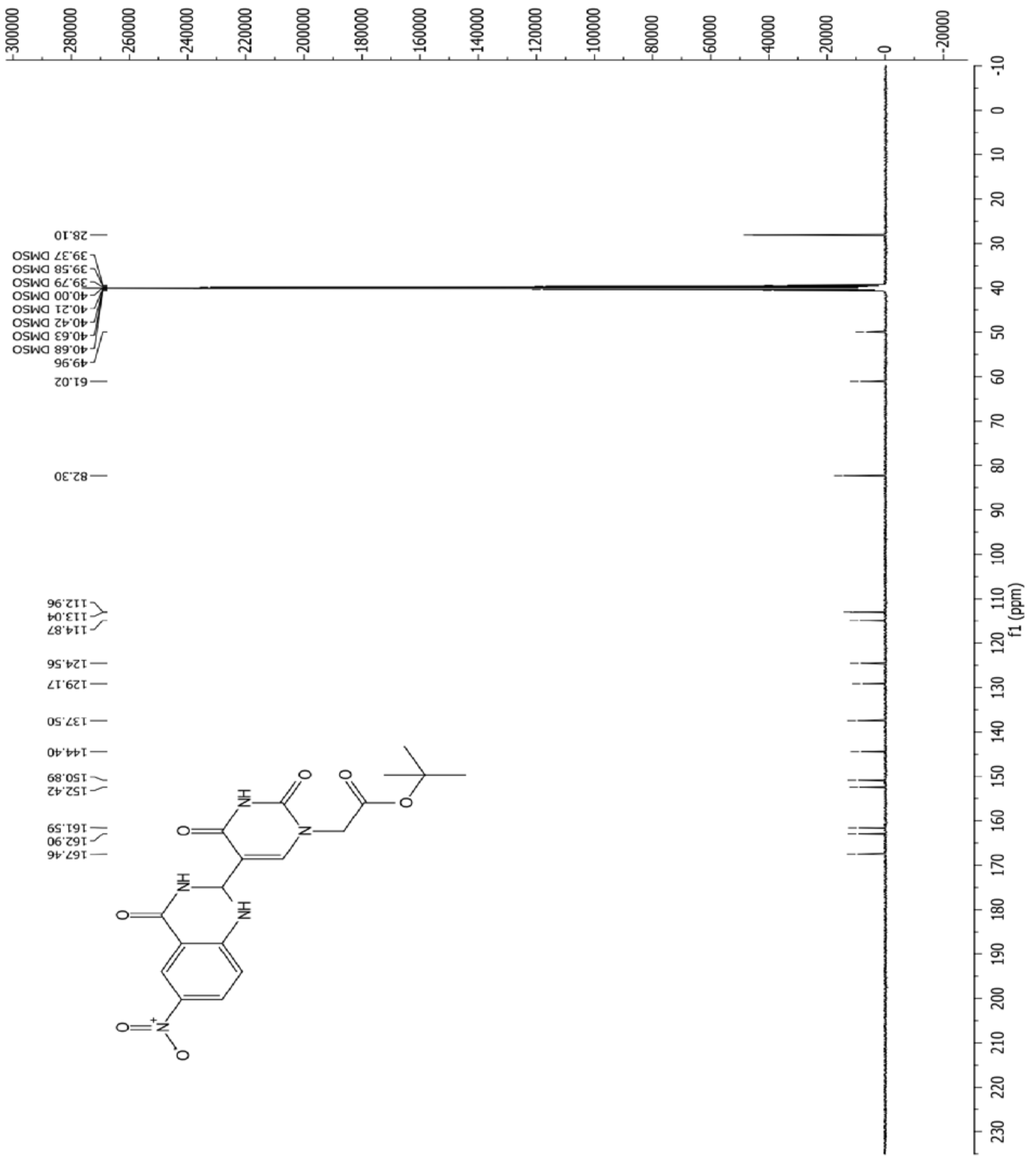

^{1}H NMR spectrum *tert*-Butyl 2-(5-(7-nitro-4-oxo-1,2,3,4-tetrahydroquinazolin-2-yl)uracil-1-yl)acetate (9)

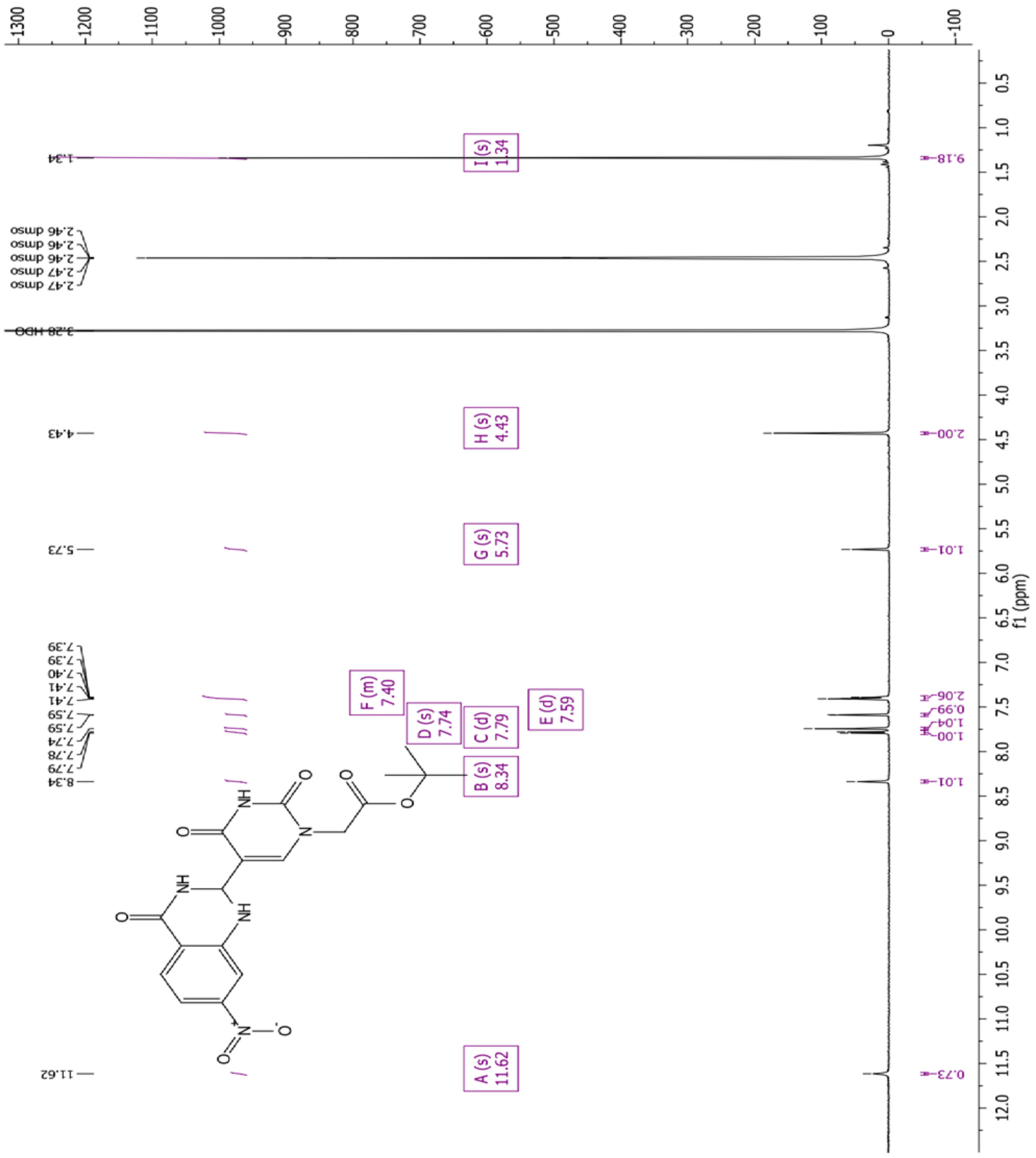

^{13}C NMR spectrum of *tert*-Butyl 2-(5-(7-nitro-4-oxo-1,2,3,4-tetrahydroquinazolin-2-yl)uracil-1-yl)acetate (9)

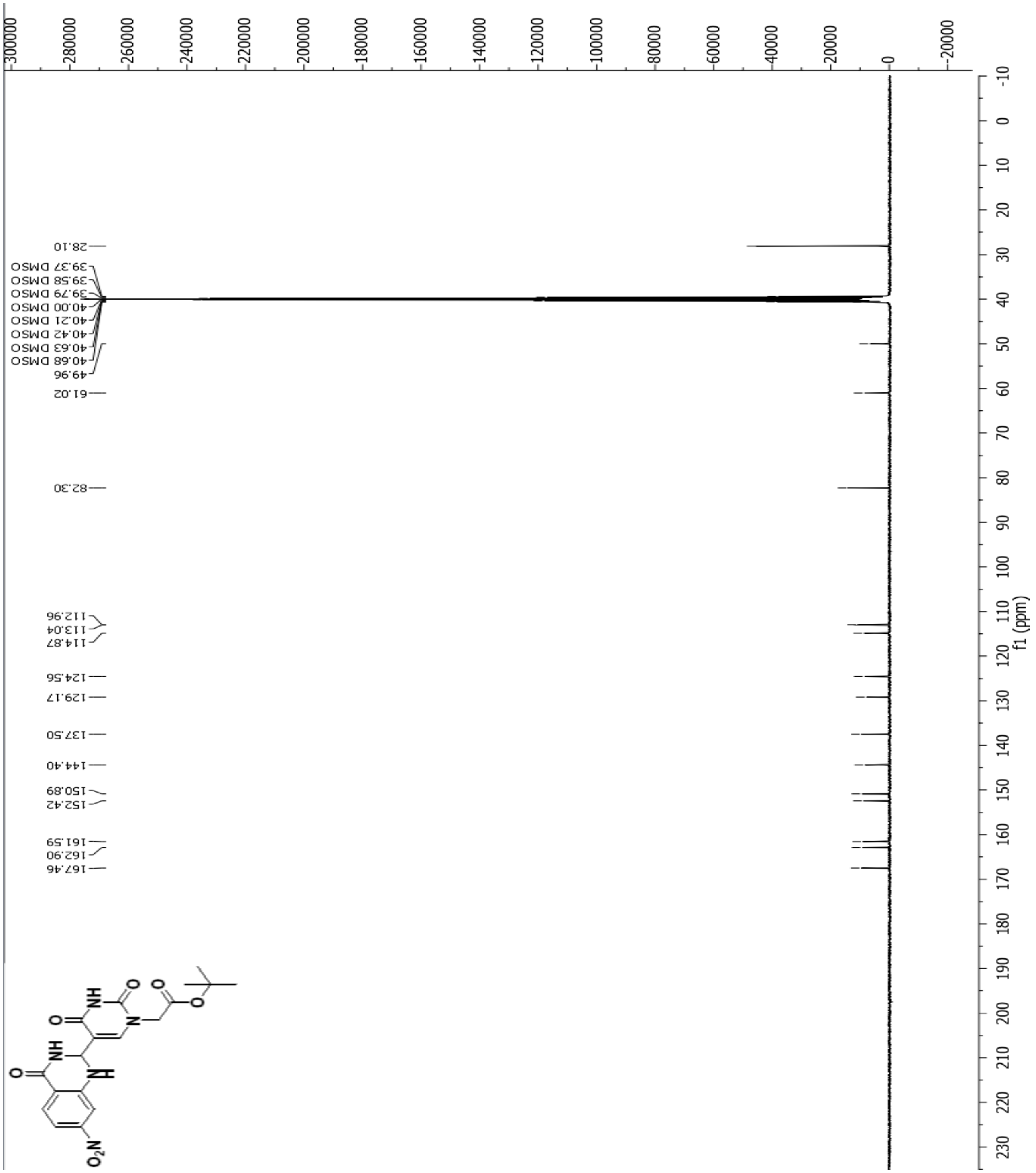

^{1}H NMR spectrum of *tert*-Butyl 2-(5-(7-methoxy-4-oxo-1,2,3,4-tetrahydroquinazolin-2-yl)uracil-1-yl)acetate (10)

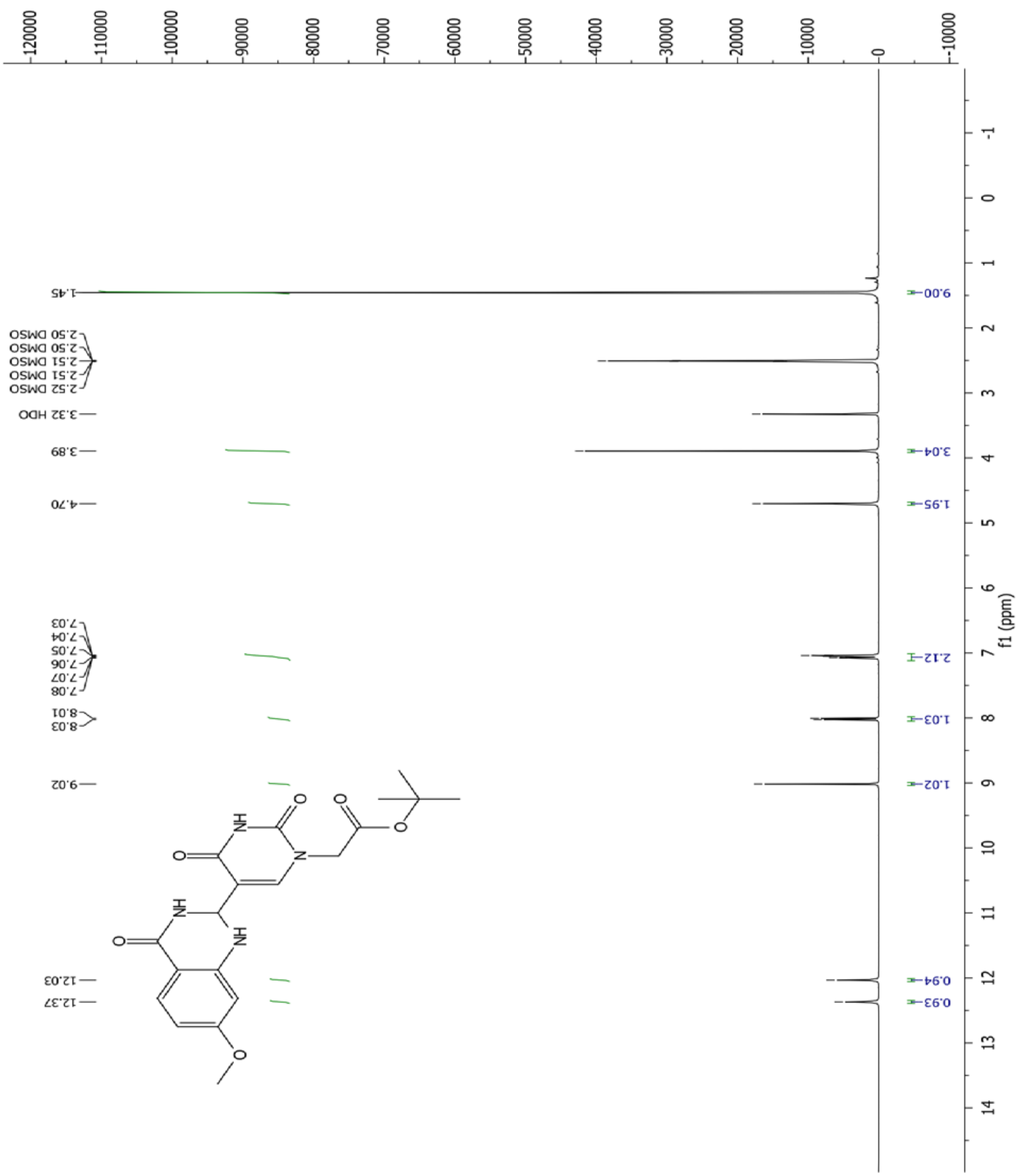

^{13}C NMR spectrum of *tert*-Butyl 2-(5-(7-methoxy-4-oxo-1,2,3,4-tetrahydroquinazolin-2-yl)uracil-1-yl)acetate (10)

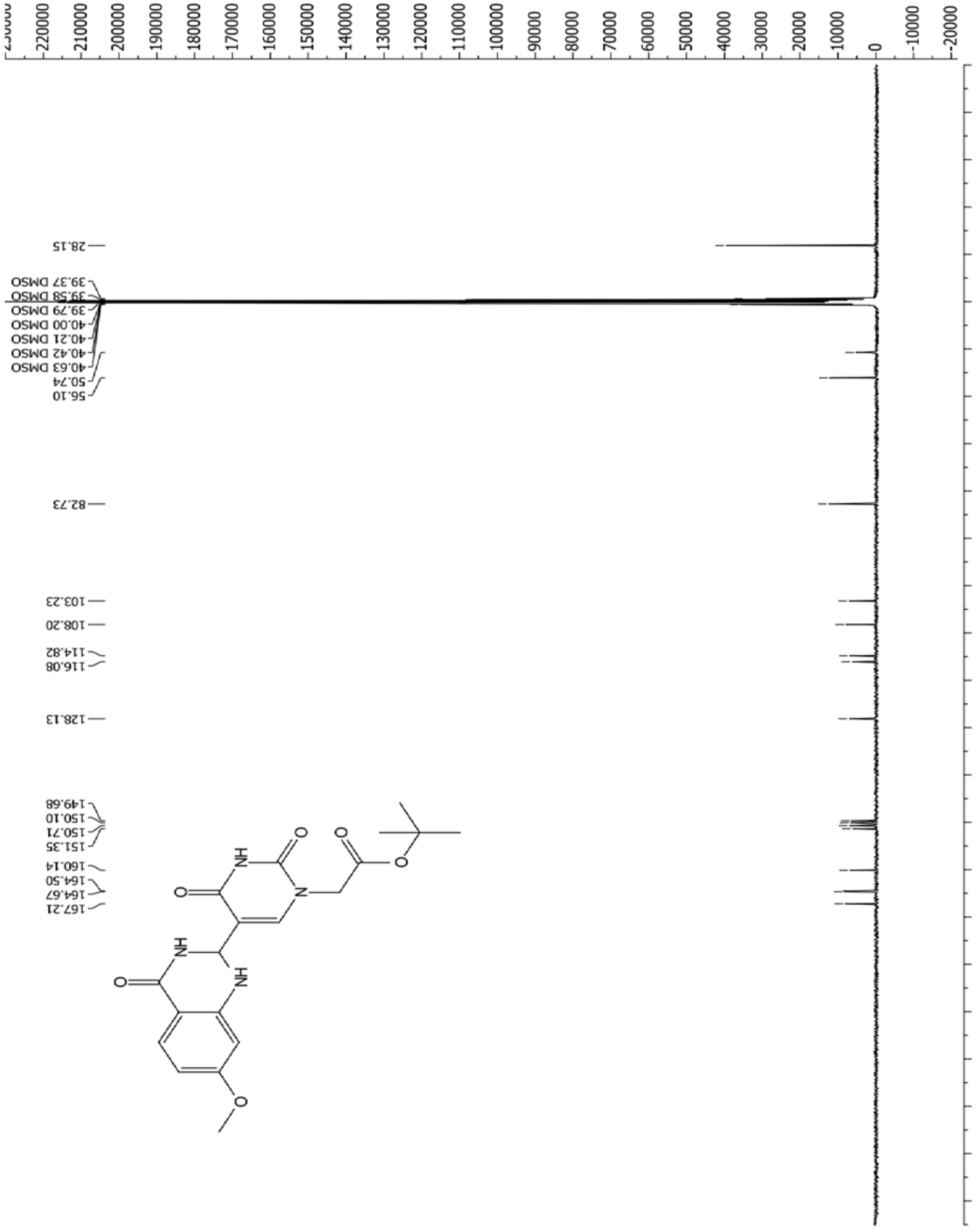

^{1}H NMR spectrum of *tert*-Butyl 2-(5-(6-methoxy-4-oxo-1,2,3,4-tetrahydroquinazolin-2-yl)uracil-1-yl)acetate (**11**)

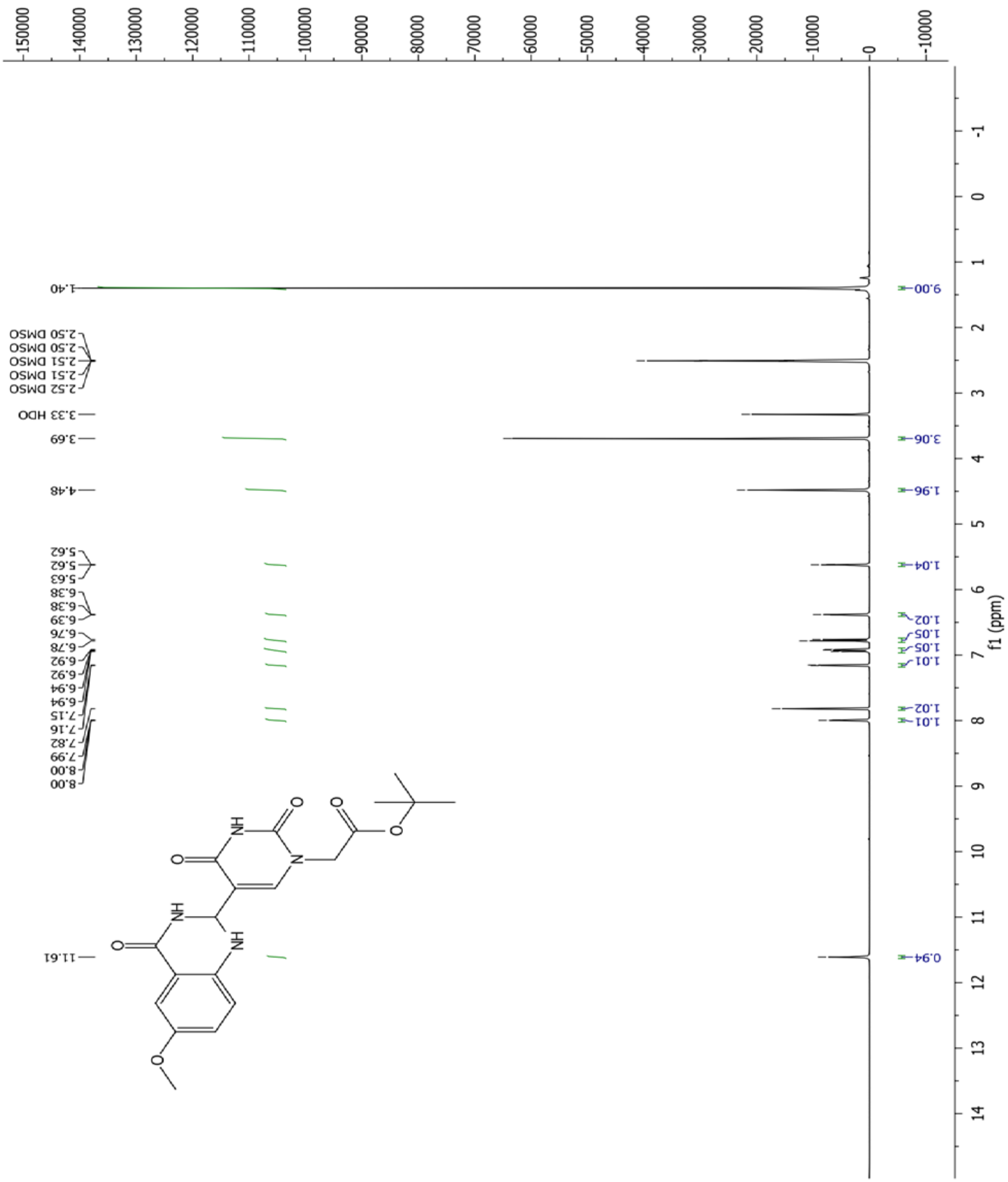

^{13}C NMR spectrum of *tert*-Butyl 2-(5-(6-methoxy-4-oxo-1,2,3,4-tetrahydroquinazolin-2-yl)uracil-1-yl)acetate (**11**)

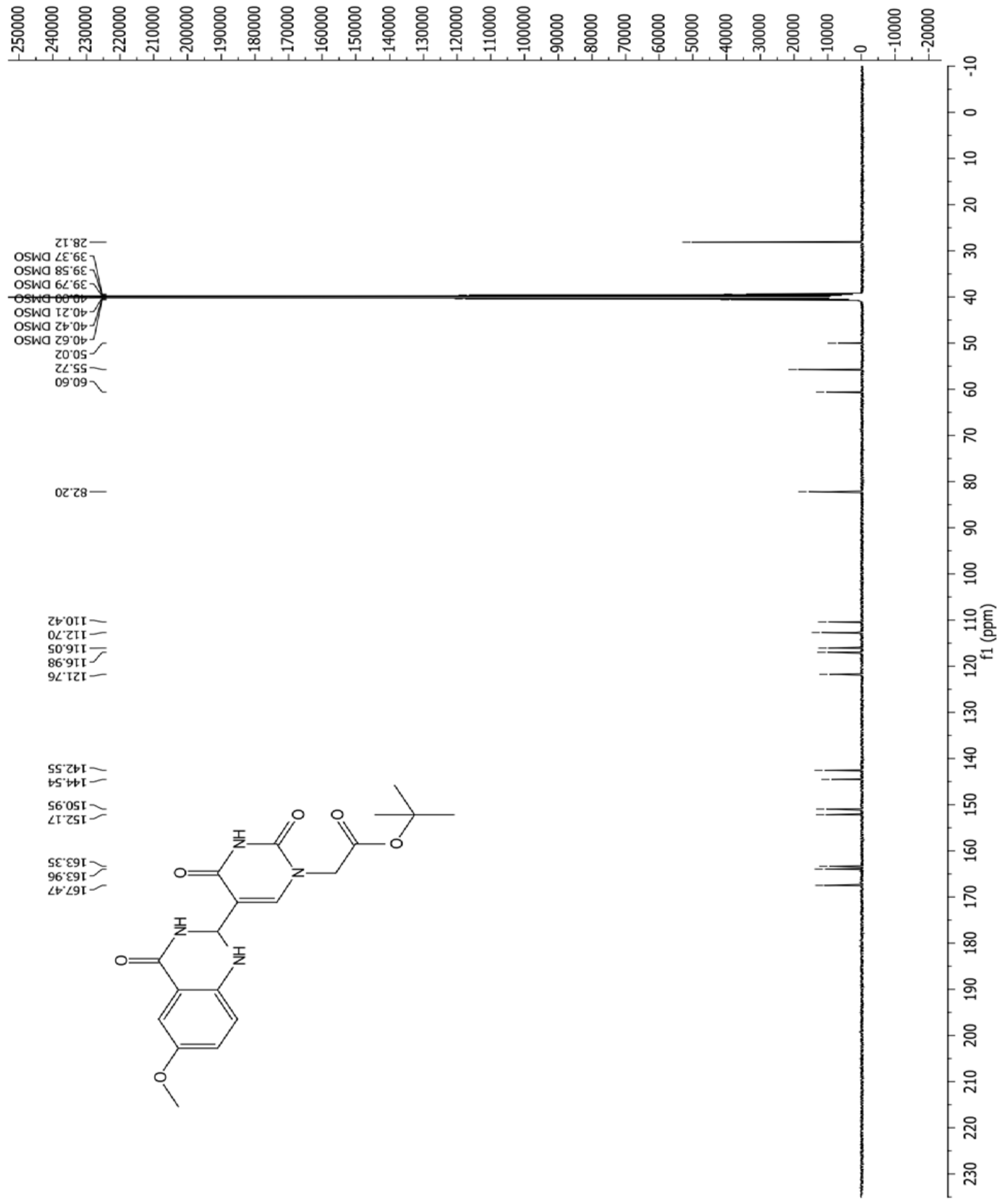

^{1}H NMR spectrum of *tert*-Butyl 2-(5-(4-oxo-3,4-dihydroquinazolin-2-yl)uracil-1-yl)acetate (**12**)

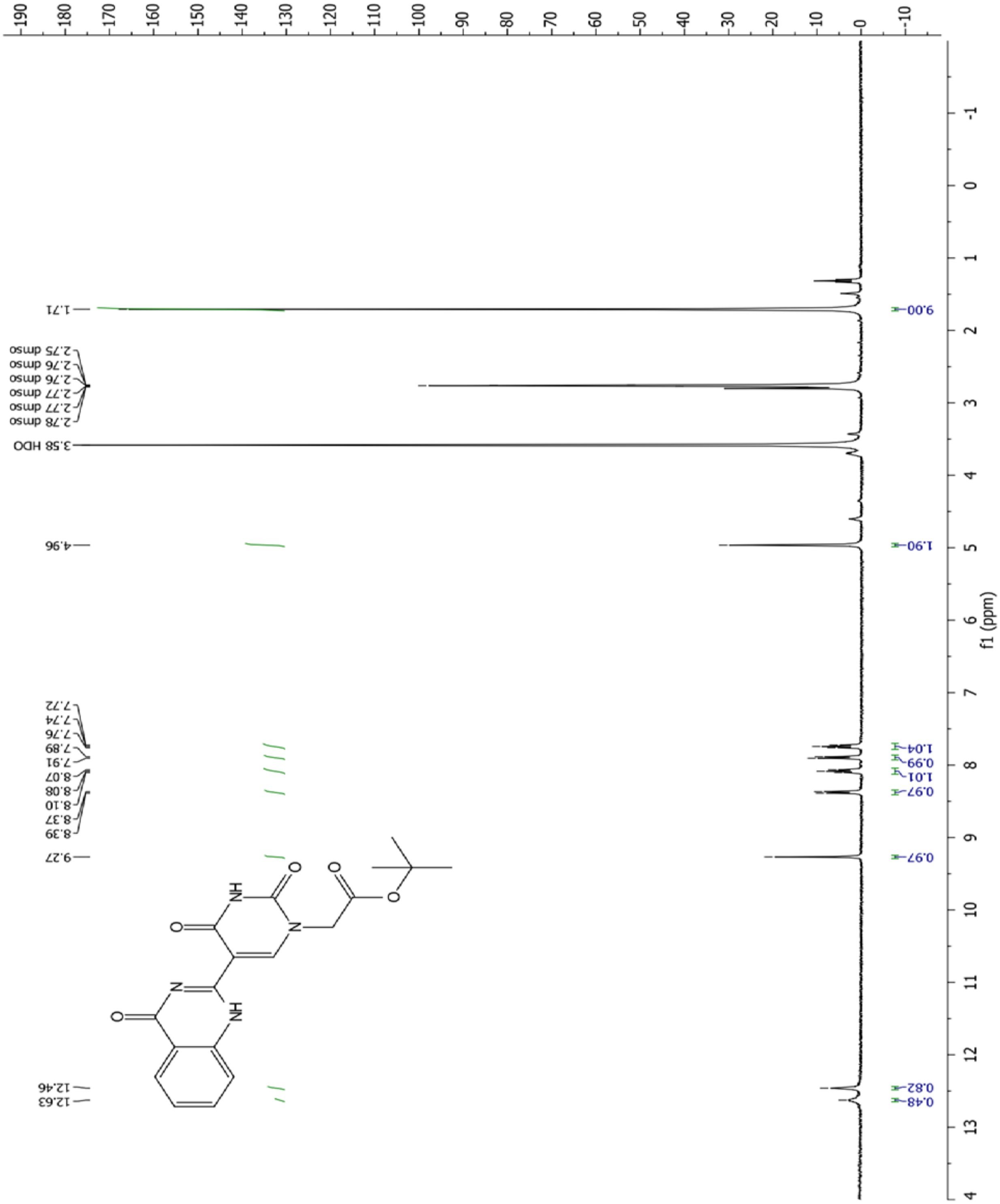

^{13}C NMR spectrum of *tert*-Butyl 2-(5-(4-oxo-3,4-dihydroquinazolin-2-yl)uracil-1-yl)acetate (**12**)

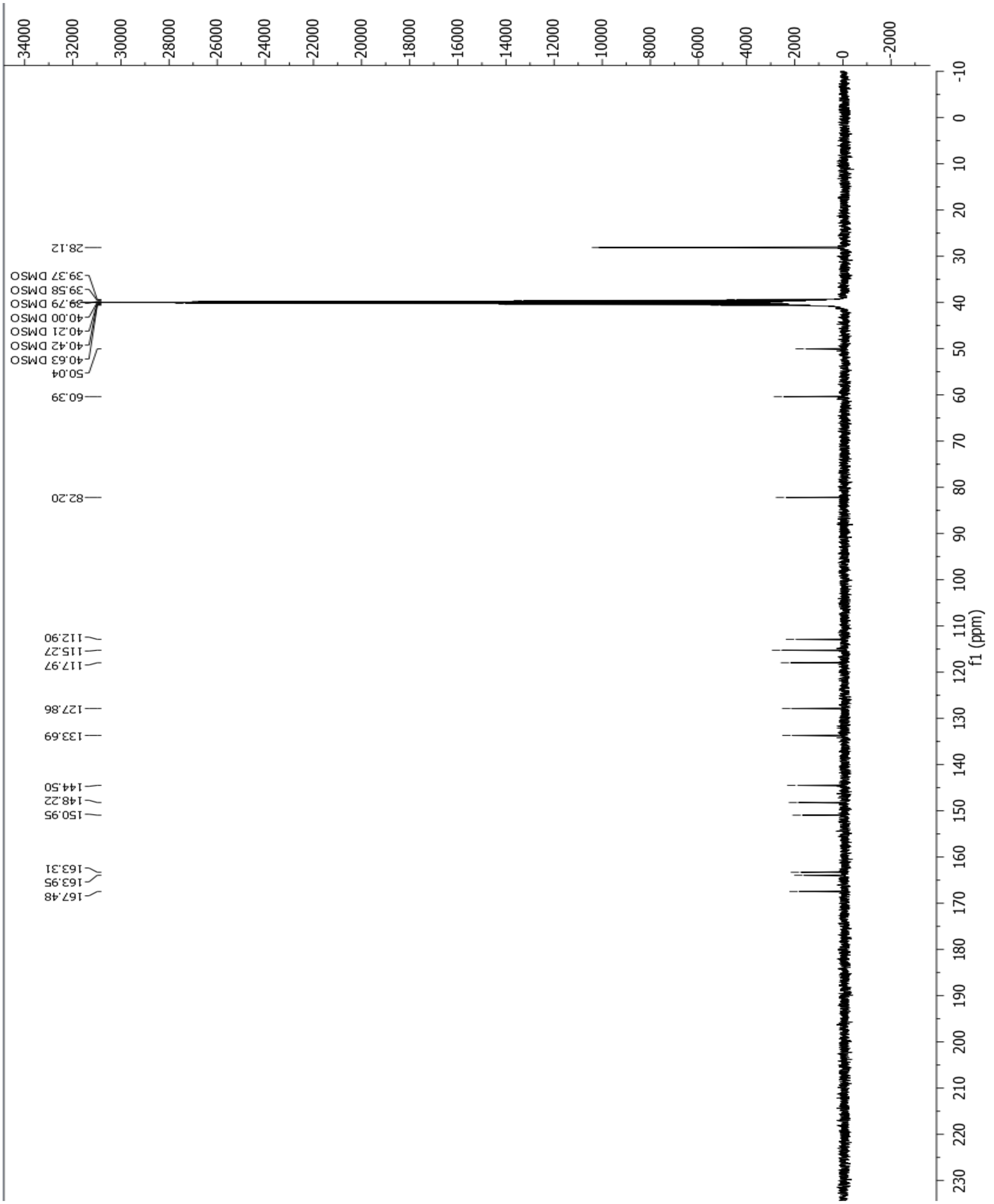

^{1}H NMR spectrum of *tert*-Butyl 2-(5-(6-nitro-4-oxo-1,4-dihydroquinazolin-2-yl)uracil-1-yl)acetate (**13**)

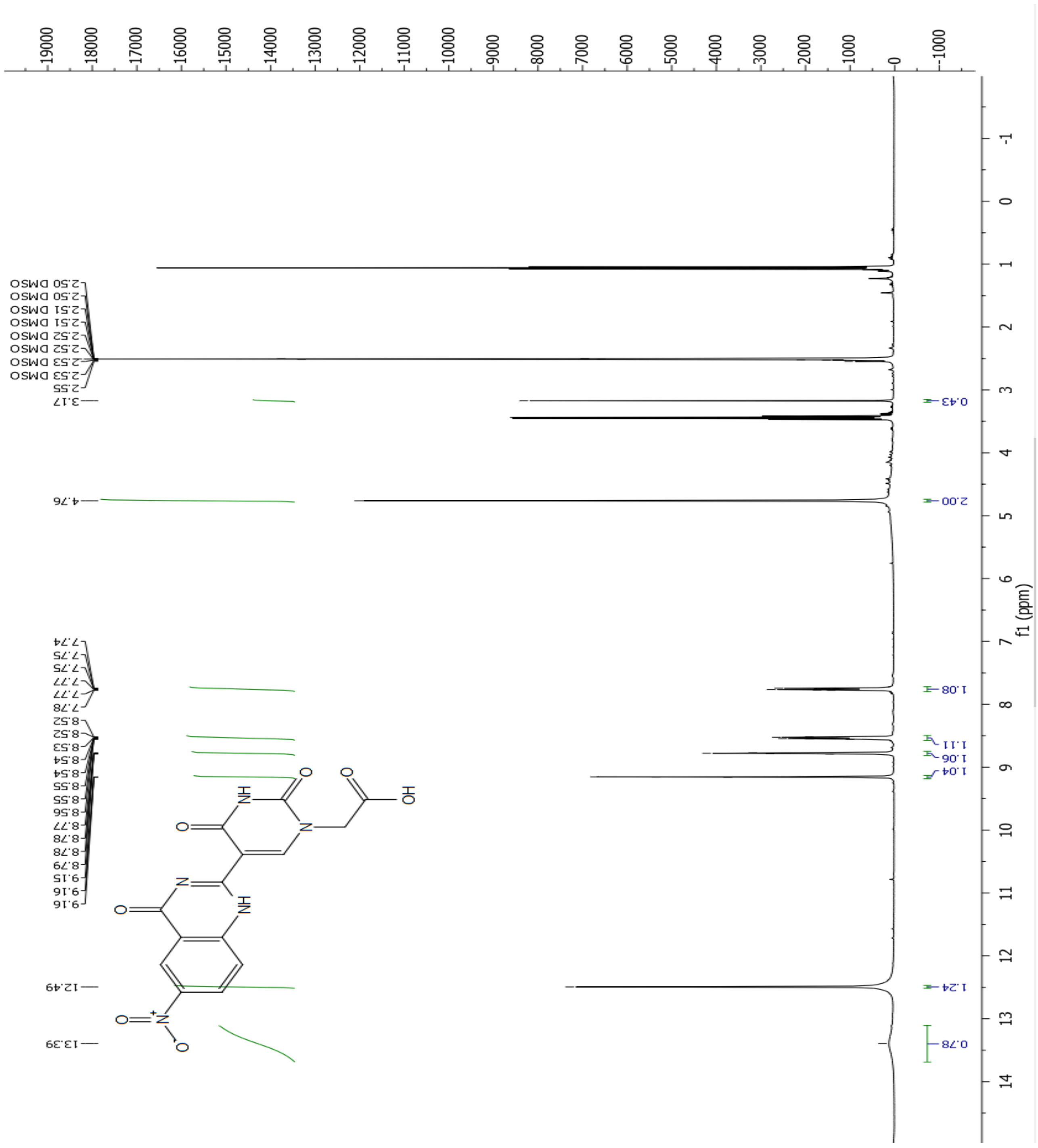

^{13}C NMR spectrum of tert-butyl 2-(5-(6-nitro-4-oxo-1,4-dihydroquinazolin-2-yl)uracil-1-yl) acetate (**13**)

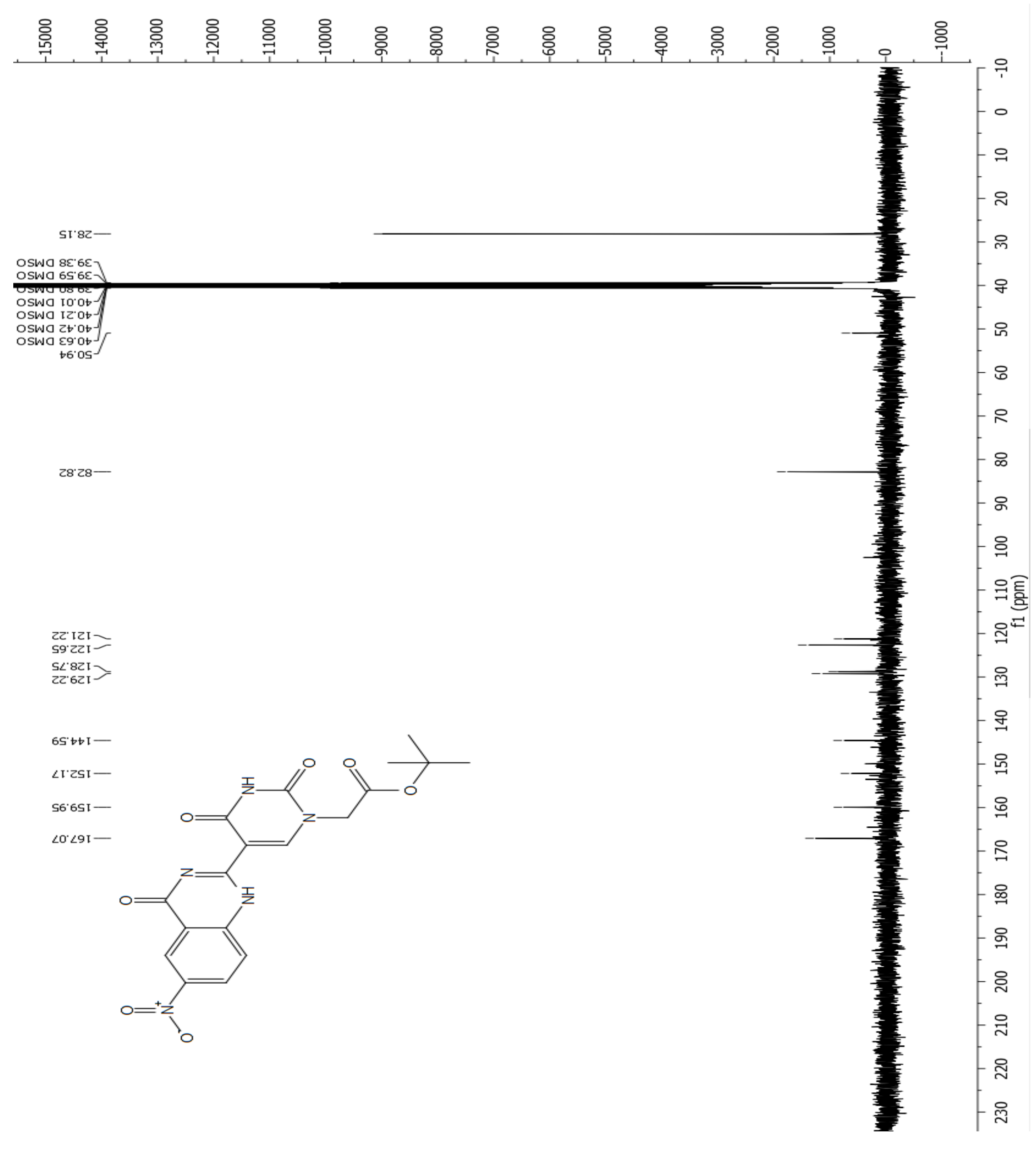

^{1}H NMR spectrum of *tert*-Butyl 2-(5-(7-nitro-4-oxo-1,4-dihydroquinazolin-2-yl)uracil-1-yl)acetate (**14**)

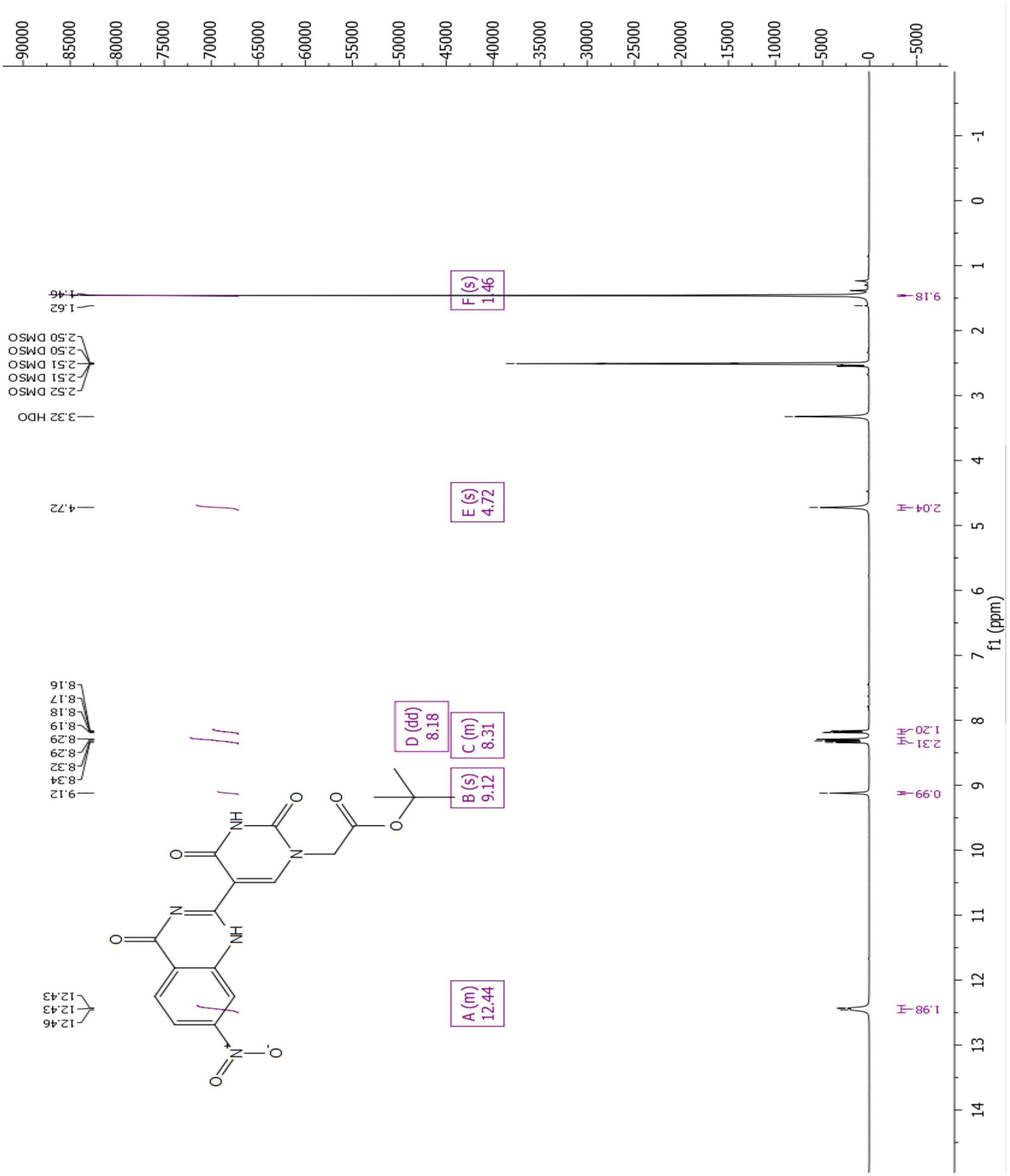

^{13}C NMR spectrum of *tert*-Butyl 2-(5-(7-nitro-4-oxo-1,4-dihydroquinazolin-2-yl)uracil-1-yl)acetate (**14**)

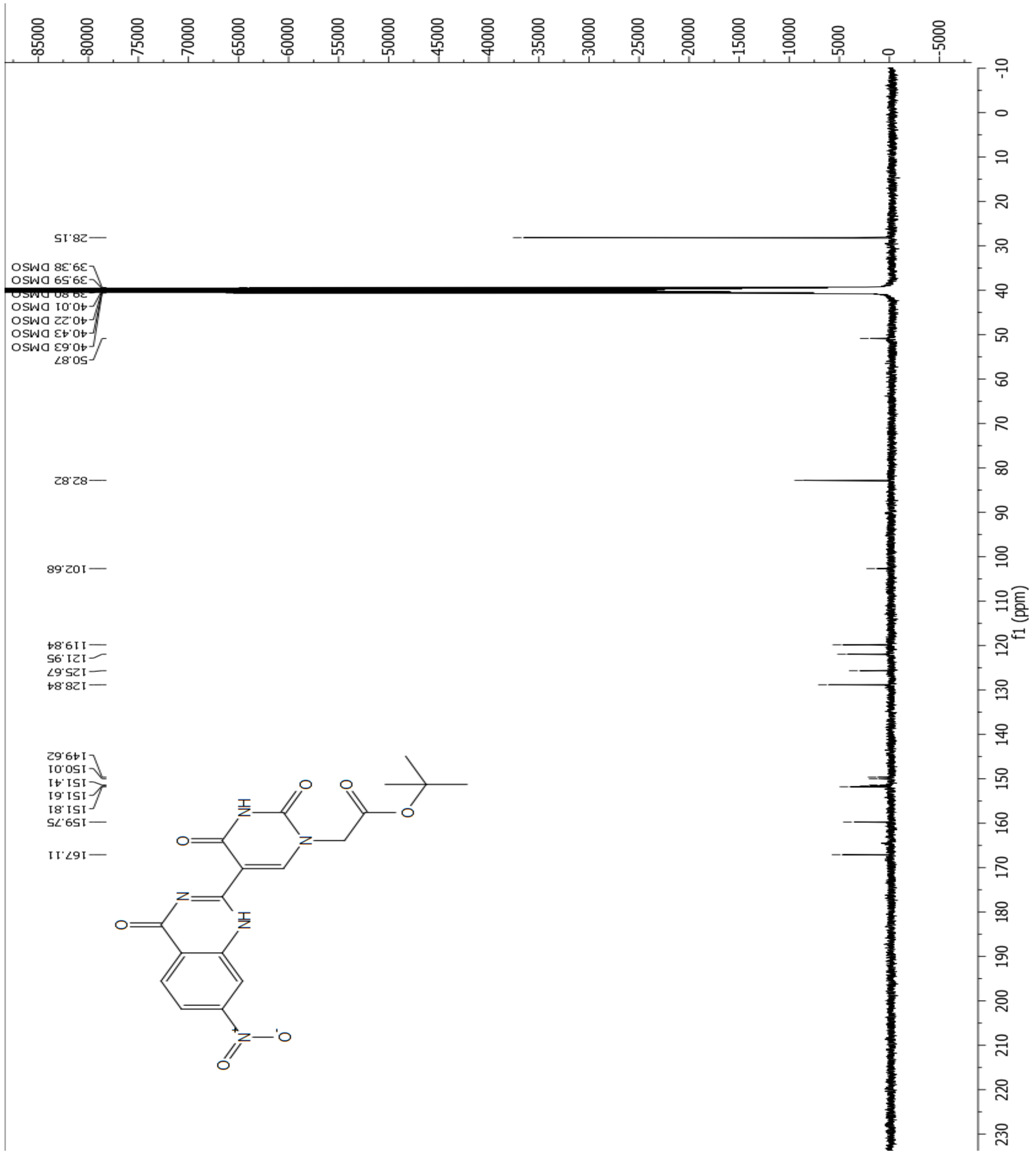

^{1}H NMR spectrum of *tert*-Butyl 2-(5-(7-methoxy-4-oxo-1,4-dihydroquinazolin-2-yl)uracil-1-yl)acetate (**15**)

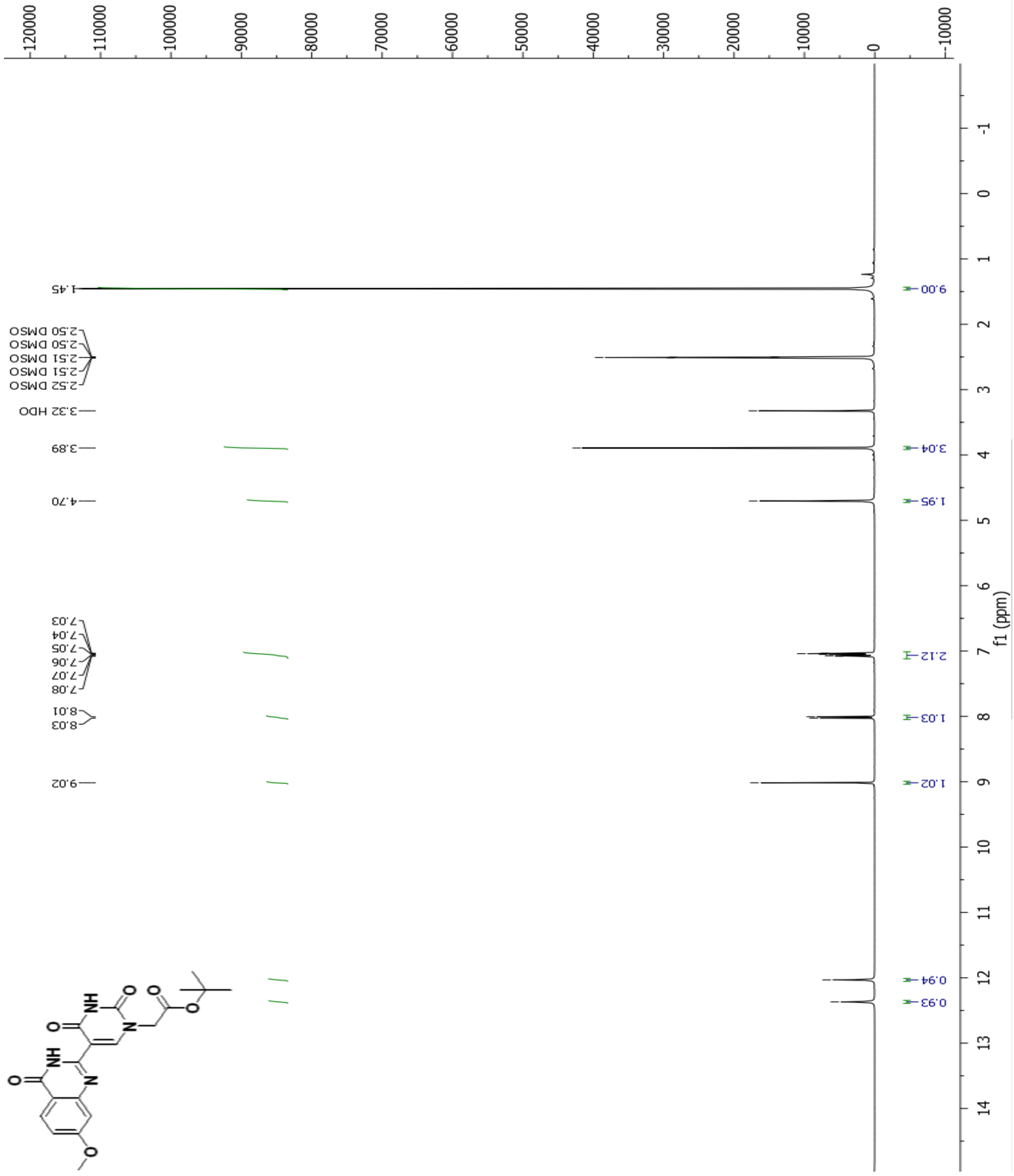

^{13}C NMR spectrum of ^{1}H NMR spectrum of *tert*-Butyl 2-(5-(7-methoxy-4-oxo-1,4-dihydroquinazolin-2-yl)uracil-1-yl)acetate (**15**)

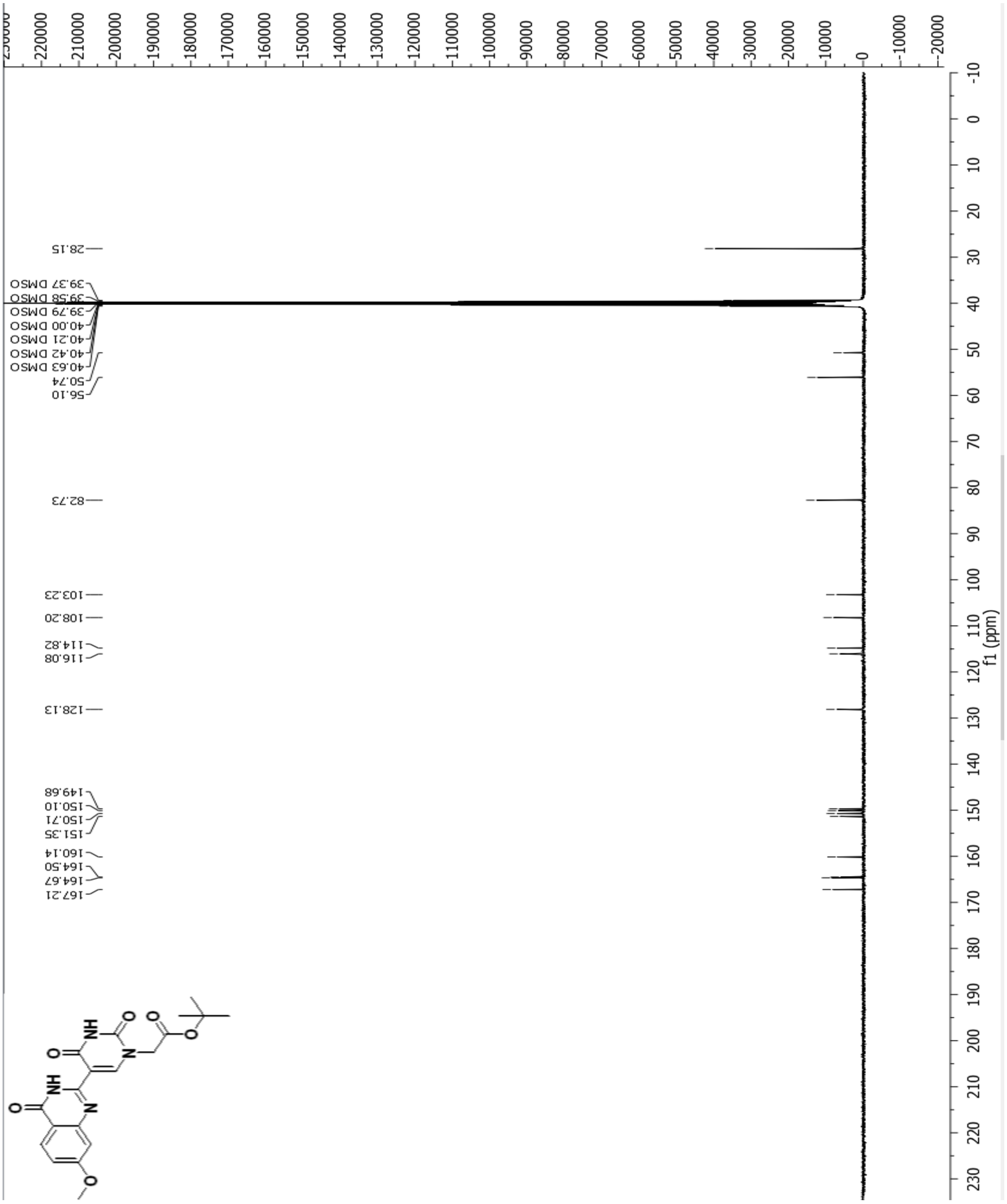

^{1}H NMR spectrum of *tert*-Butyl 2-(5-(6-methoxy-4-oxo-3,4-dihydroquinazolin-2-yl)uracil-1-yl)acetate (**16**)

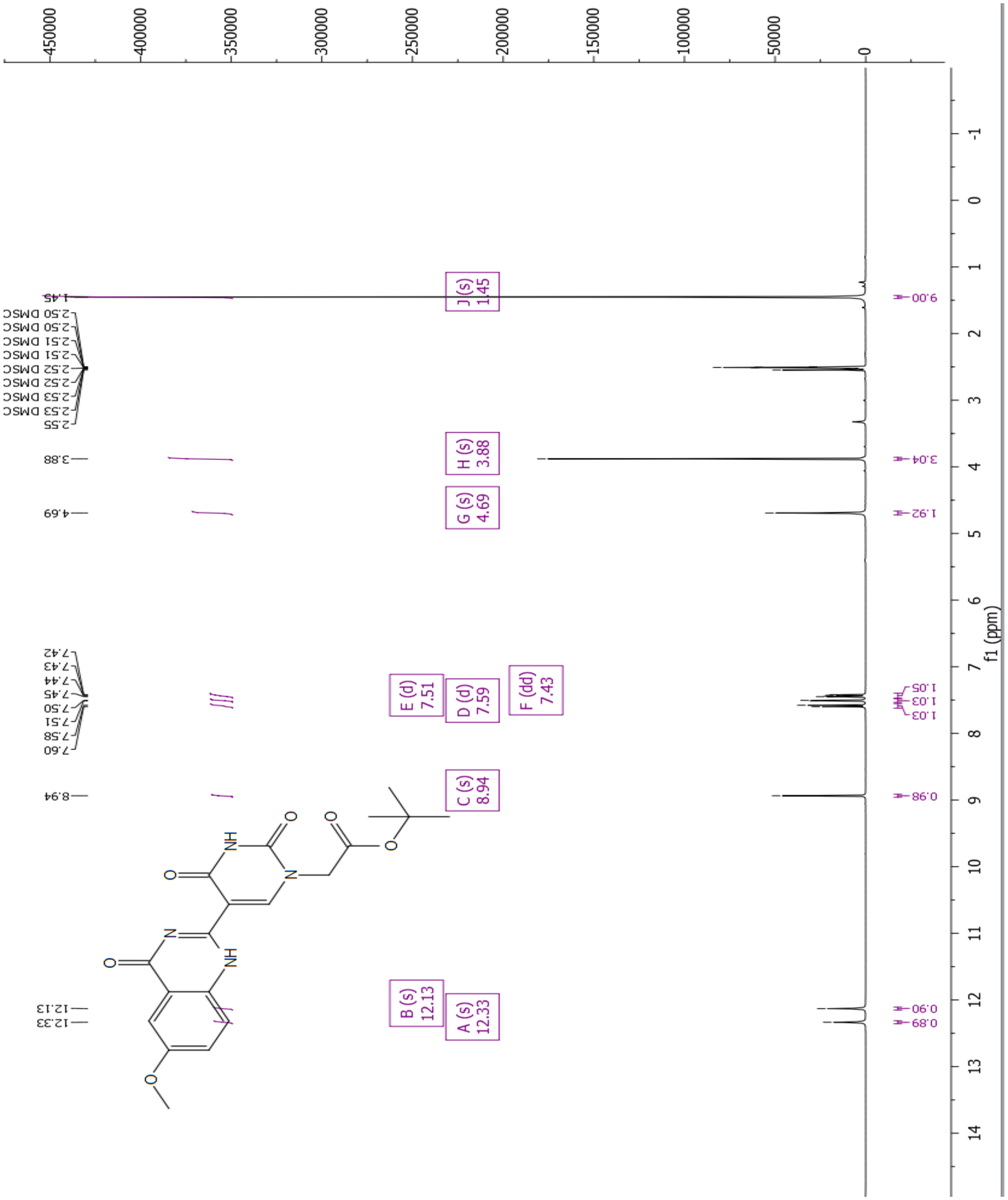

^{13}C NMR spectrum of *tert*-Butyl 2-(5-(6-methoxy-4-oxo-3,4-dihydroquinazolin-2-yl)uracil-1-yl)acetate (**16**)

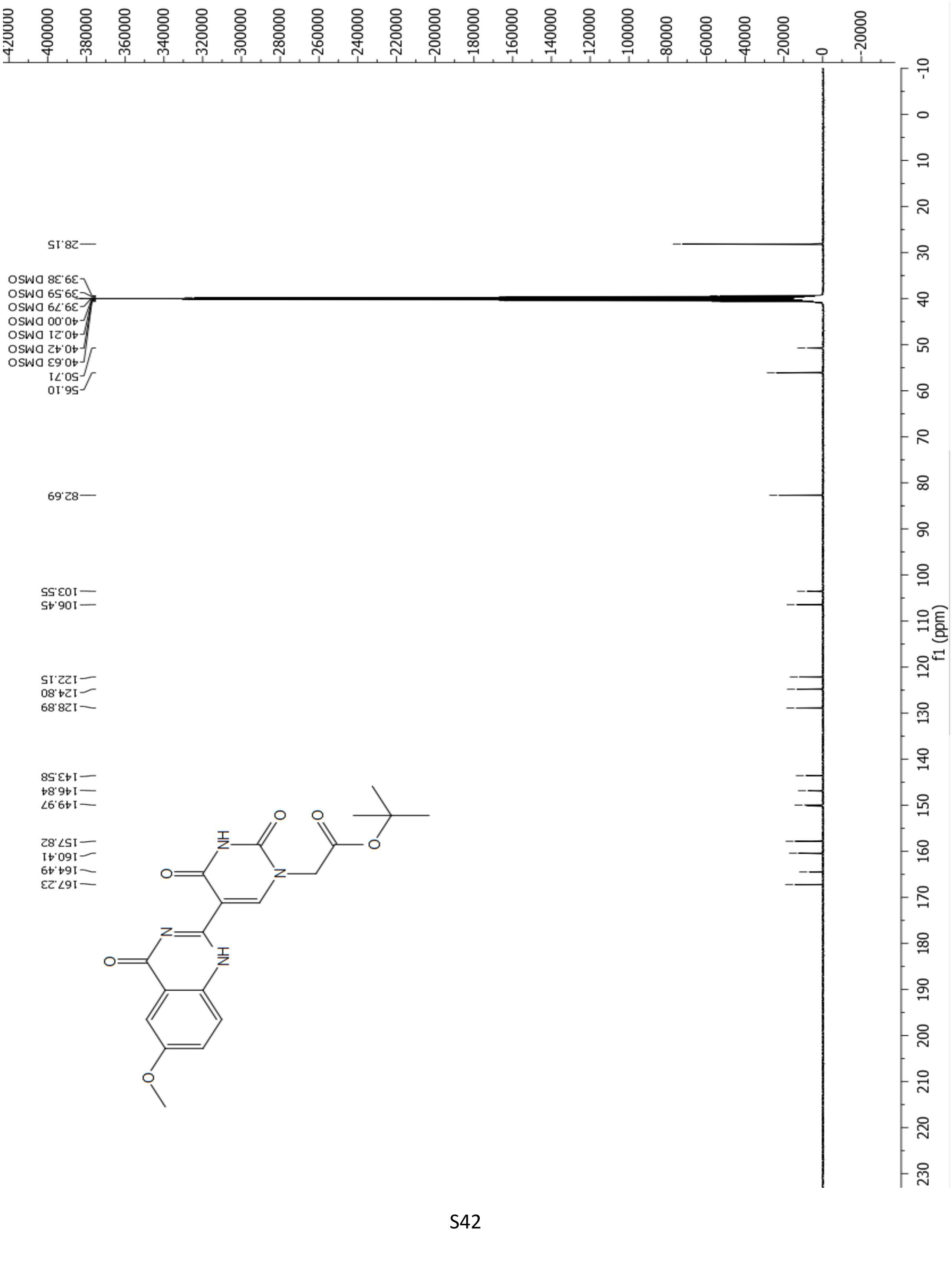

^{1}H NMR spectrum of *tert*-Butyl (2-aminoethyl)glycinate (**17**)

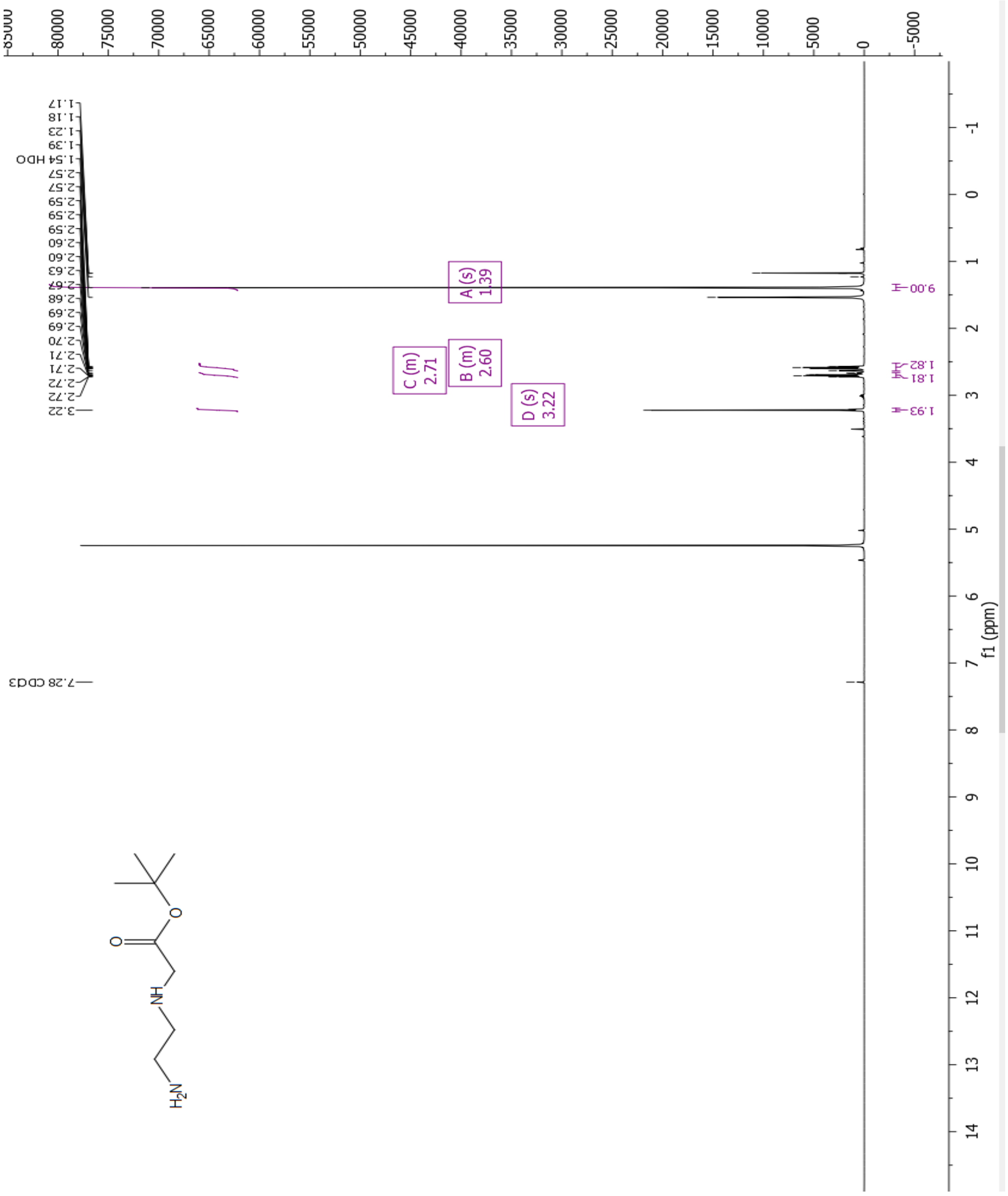

^{1}H NMR spectrum of *tert*-Butyl (2-((((9H-fluoren-9-yl)methoxy)carbonyl)amino)ethyl)-glycinate (**18**)

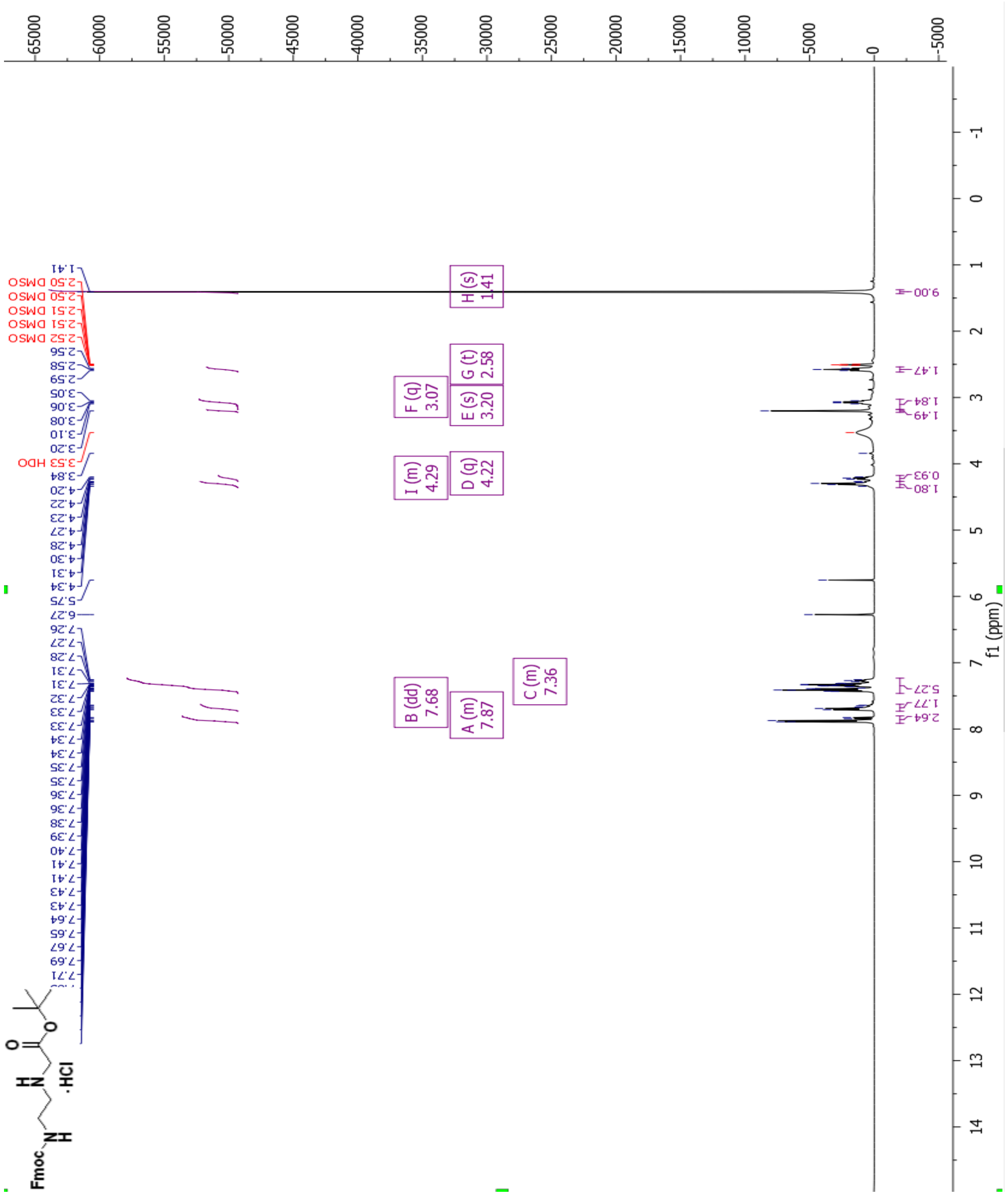

^{13}C NMR spectrum of *tert*-Butyl (2-((((9H-fluoren-9-yl)methoxy)carbonyl)amino)ethyl)-glycinate (**18**)

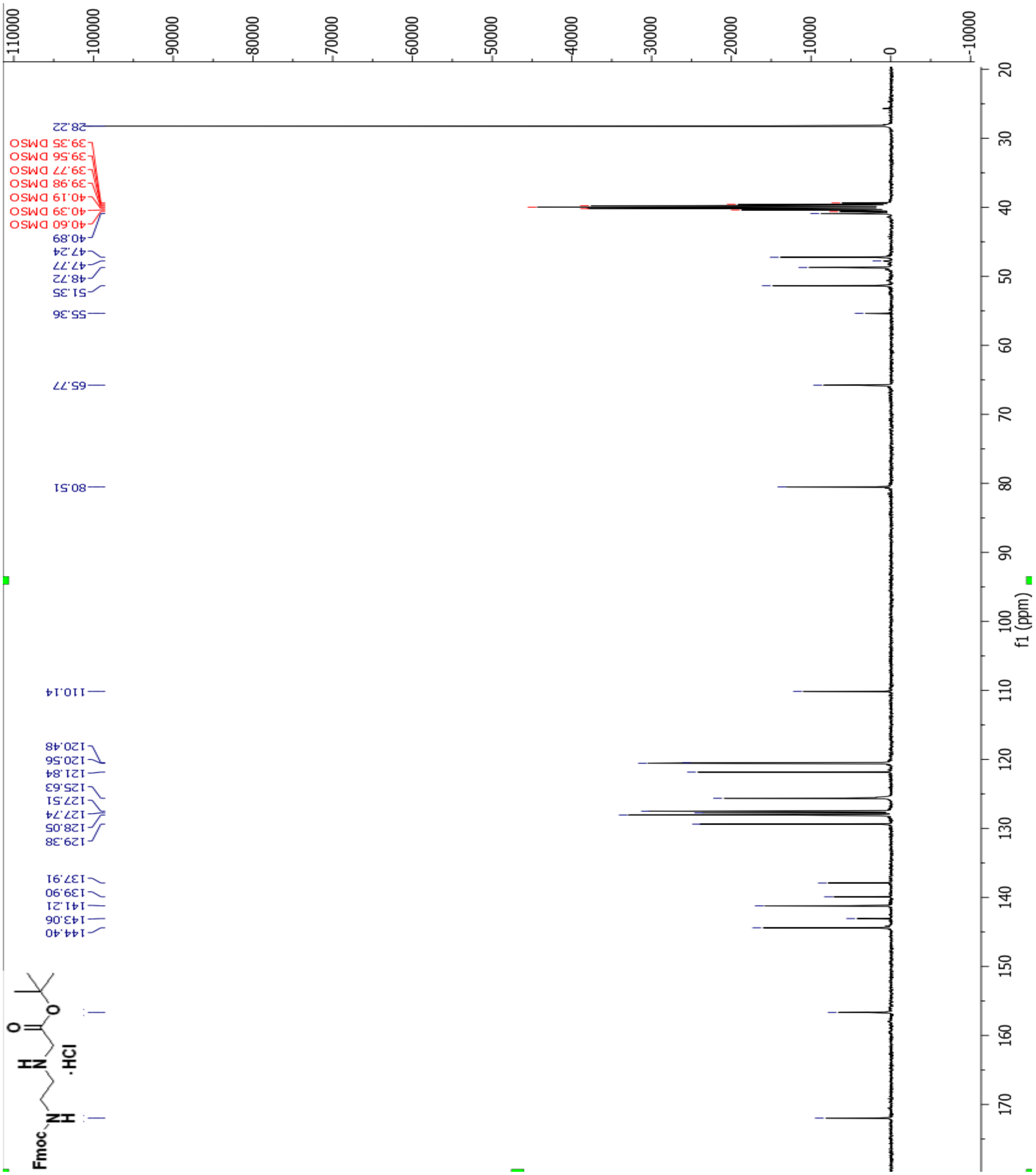

^{1}H NMR spectrum of 2-(2,4-dioxo-5-(4-oxo-3,4-dihydroquinazolin-2-yl)uracil-1-yl)acetic acid (**19**)

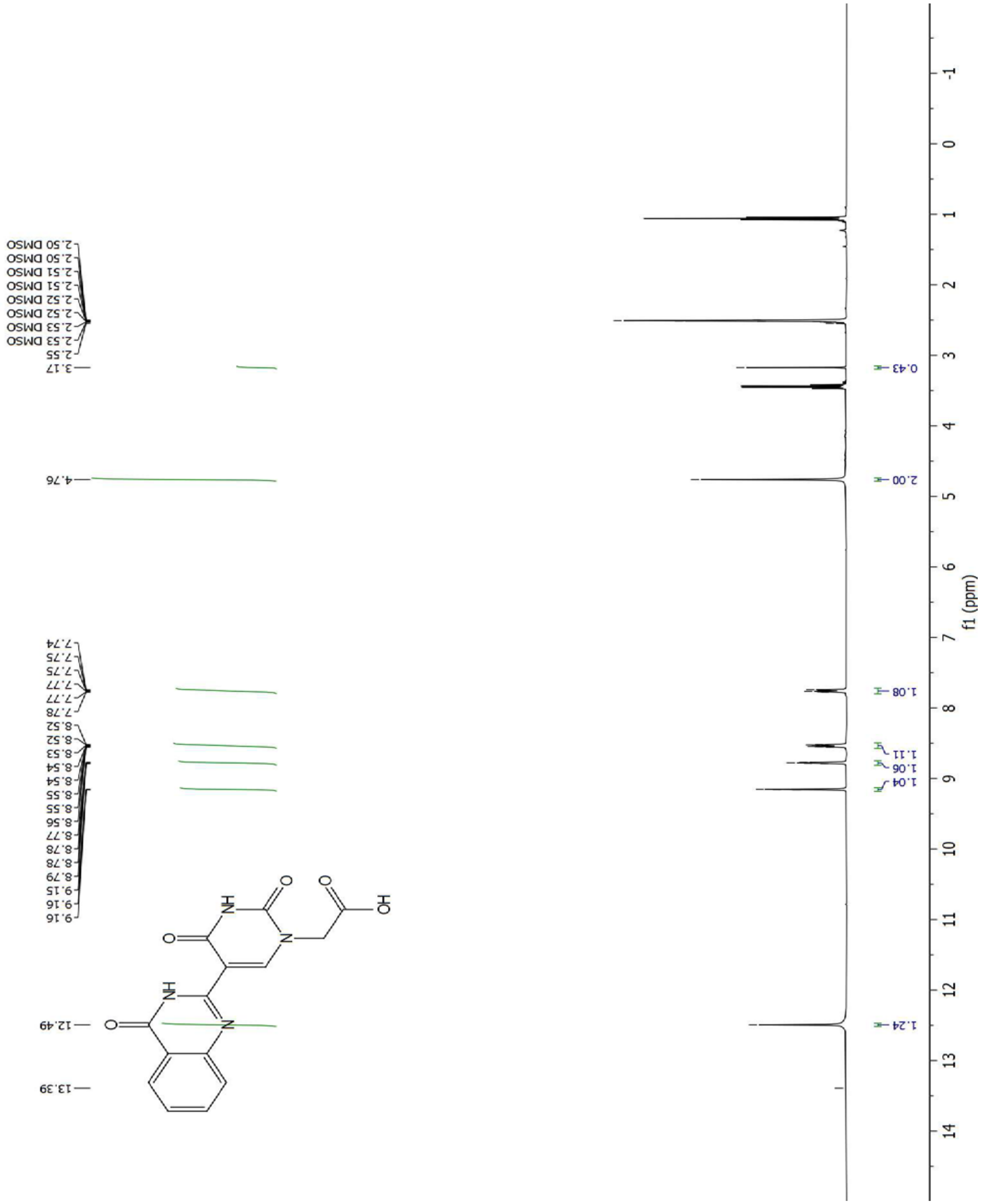

^{13}C NMR spectrum of 2-(2,4-dioxo-5-(4-oxo-3,4-dihydroquinazolin-2-yl)uracil-1-yl)acetic acid (**19**)

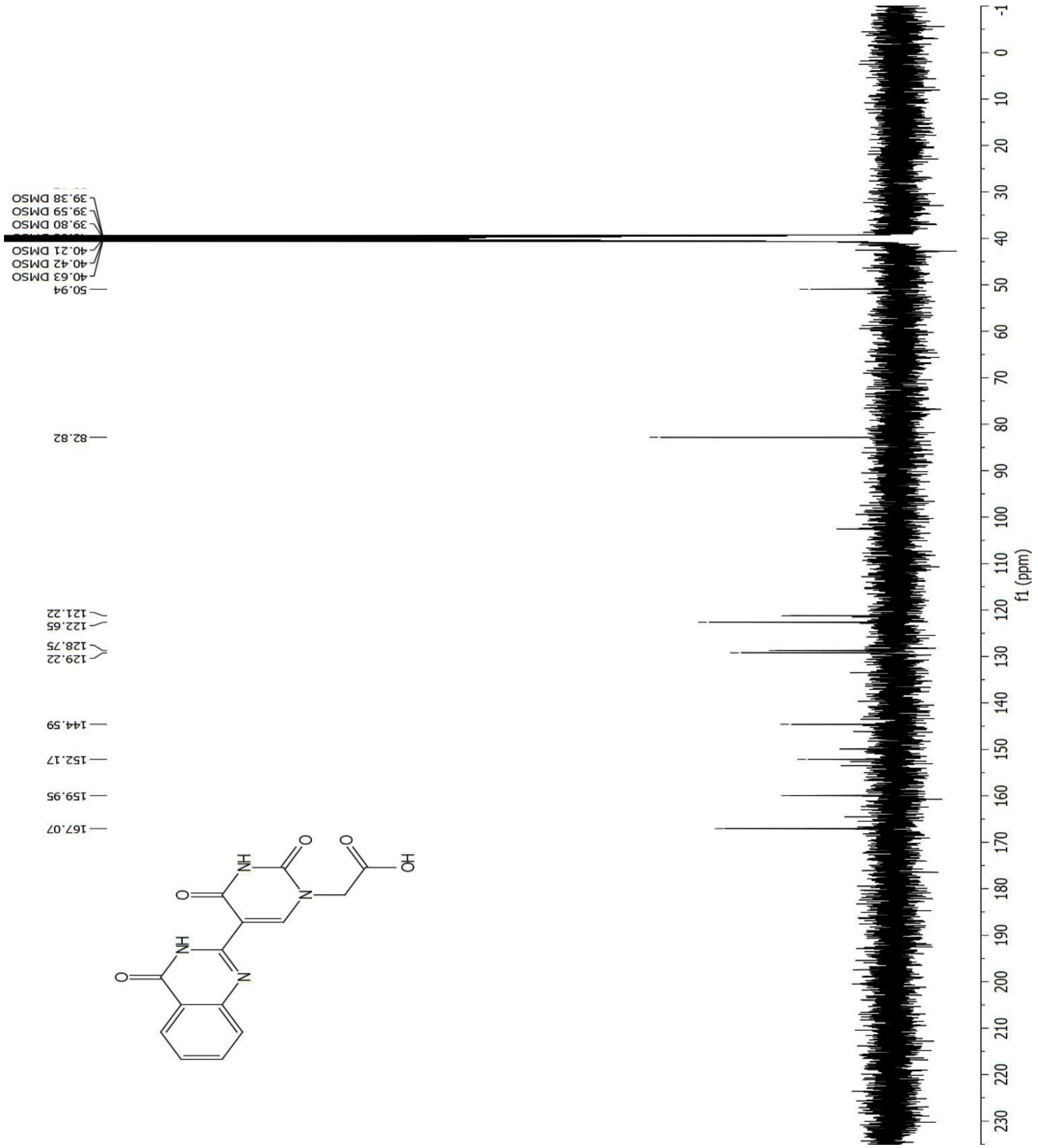

¹H NMR spectrum of 2-(5-(6-nitro-4-oxo-3,4-dihydroquinazolin-2-yl)uracil-1-yl)acetic acid (**20**)

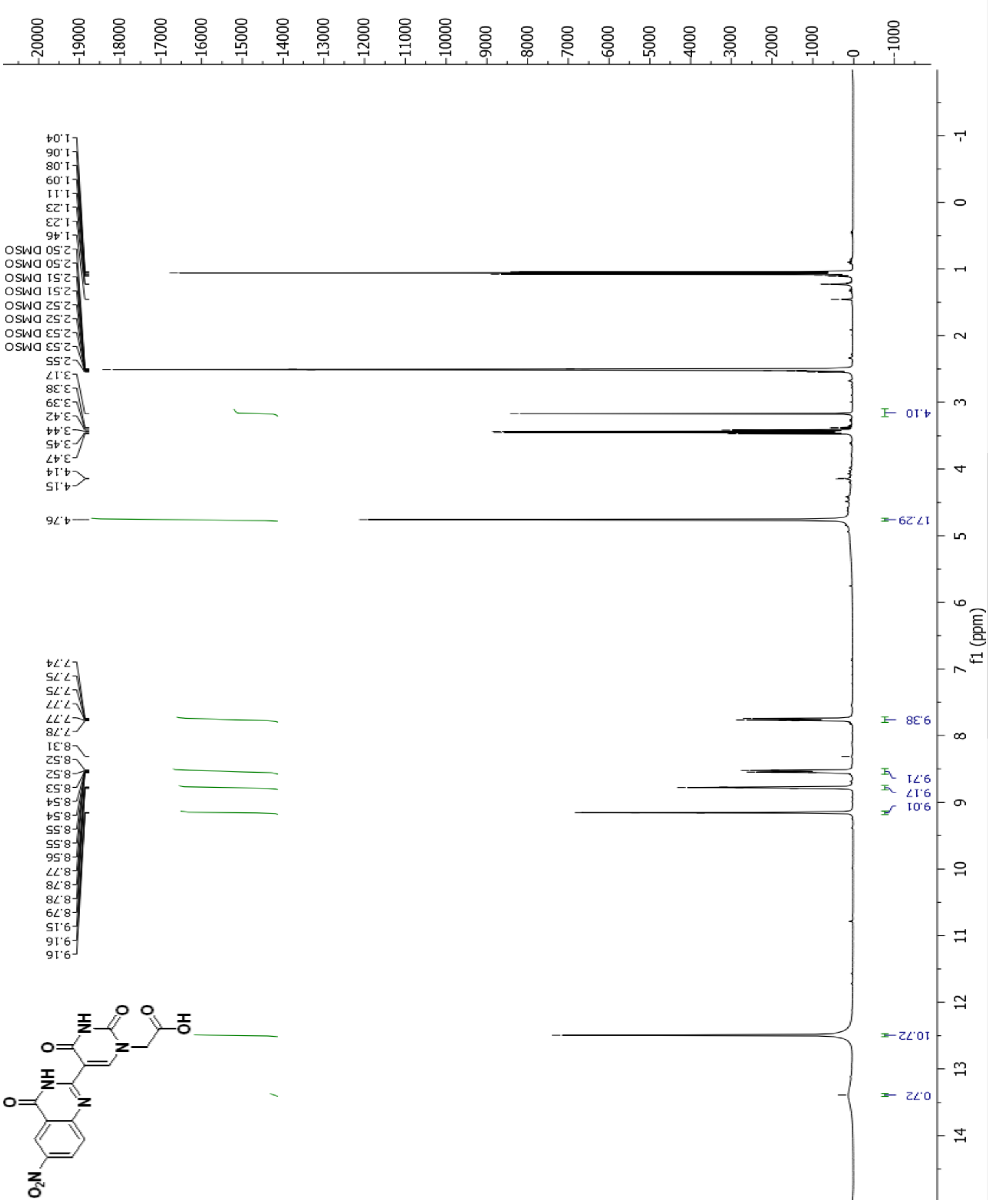

^{1}H NMR spectrum of 2-(5-(6-methoxy-4-oxo-3,4-dihydroquinazolin-2-yl)uracil-1-yl)acetic acid (**21**)

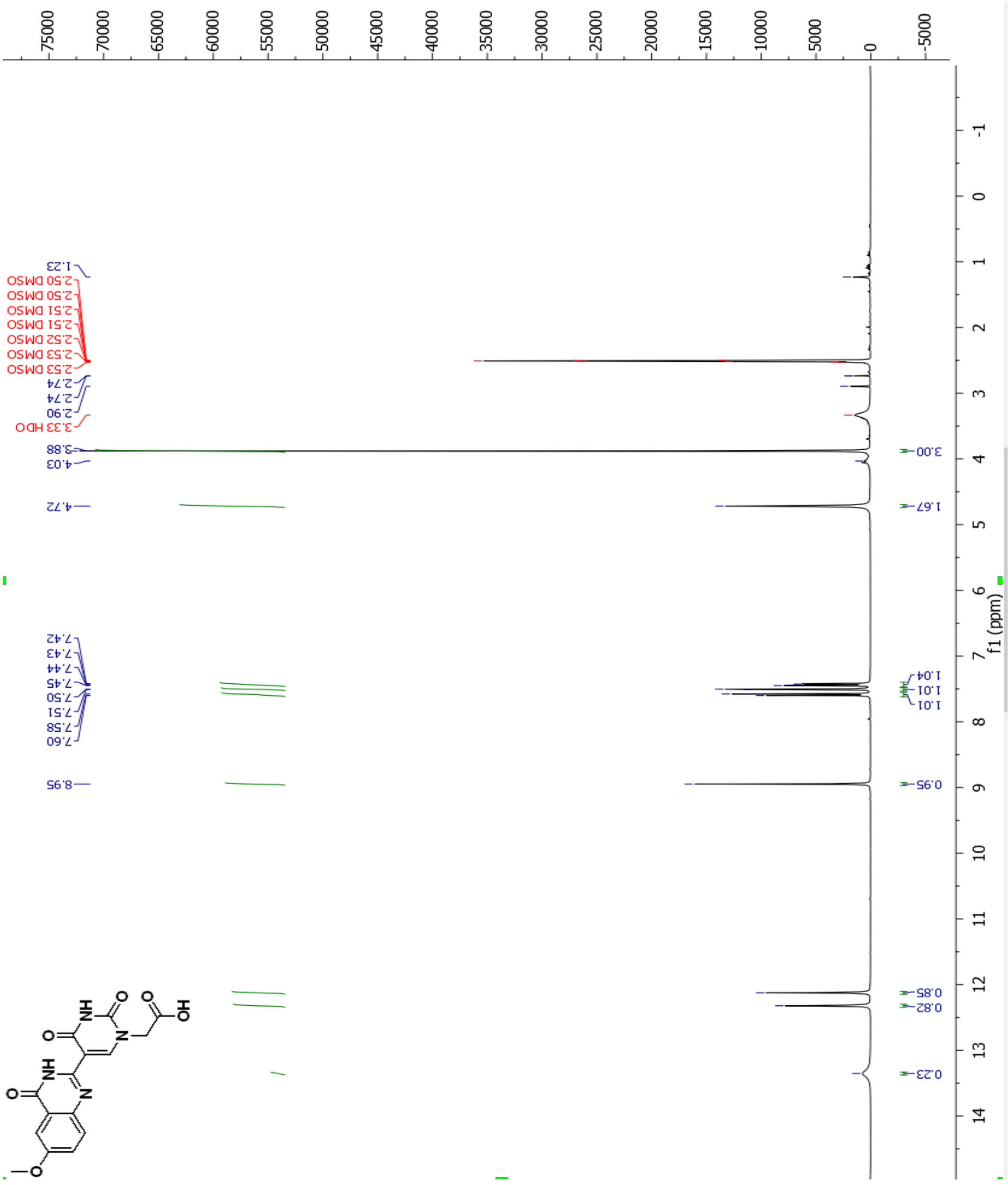

^{13}C NMR spectrum of 2-(5-(6-methoxy-4-oxo-3,4-dihydroquinazolin-2-yl)uracil-1-yl)acetic acid (**21**)

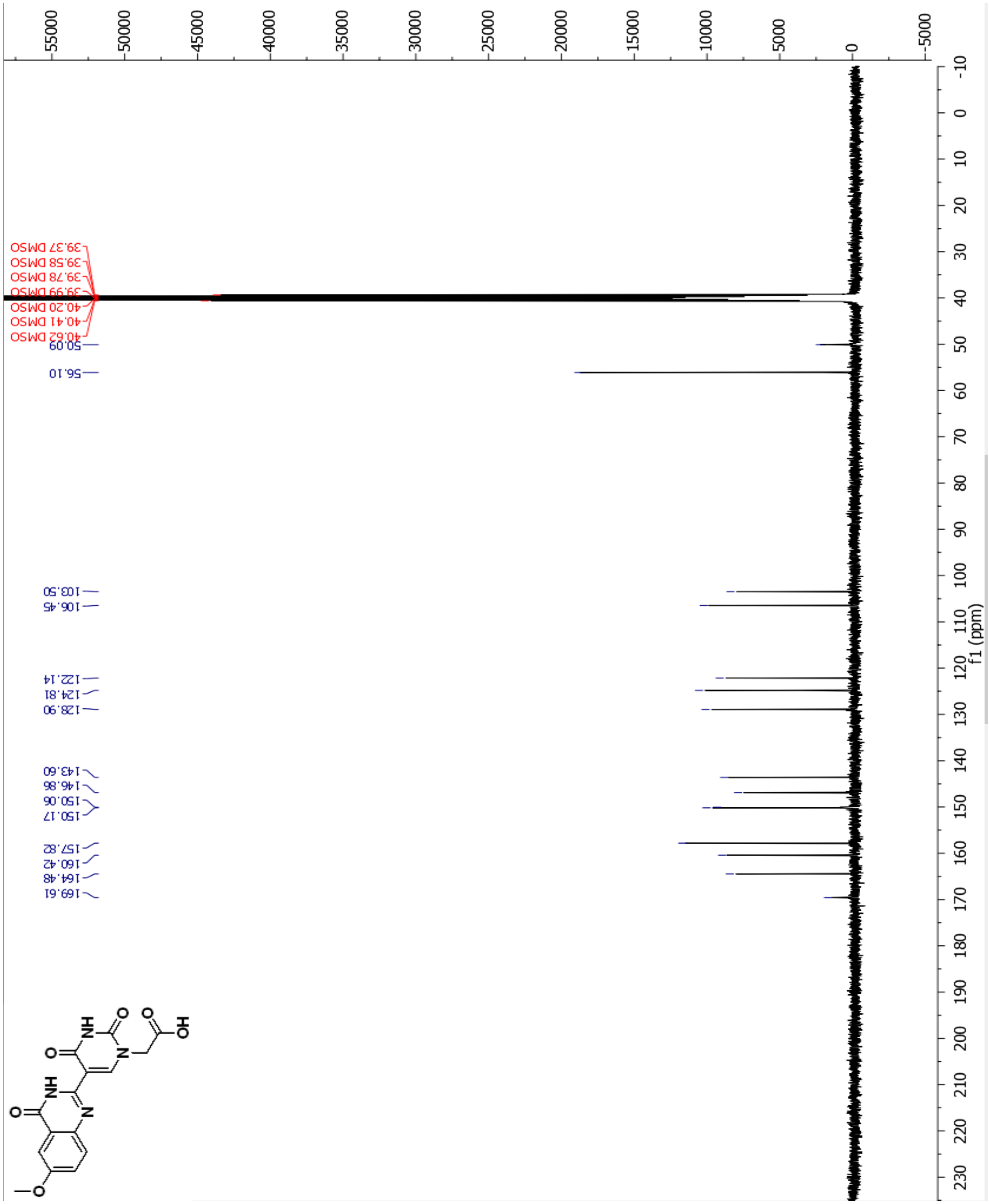

^{1}H NMR spectrum of *tert*-Butyl N-(2-((((9H-fluoren-9-yl)methoxy)carbonyl)amino)ethyl)-N-(2-(2,4-dioxo-5-(4-oxo-3,4-dihydroquinazolin-2-yl)uracil-1-yl)acetyl)glycinate (**22**)

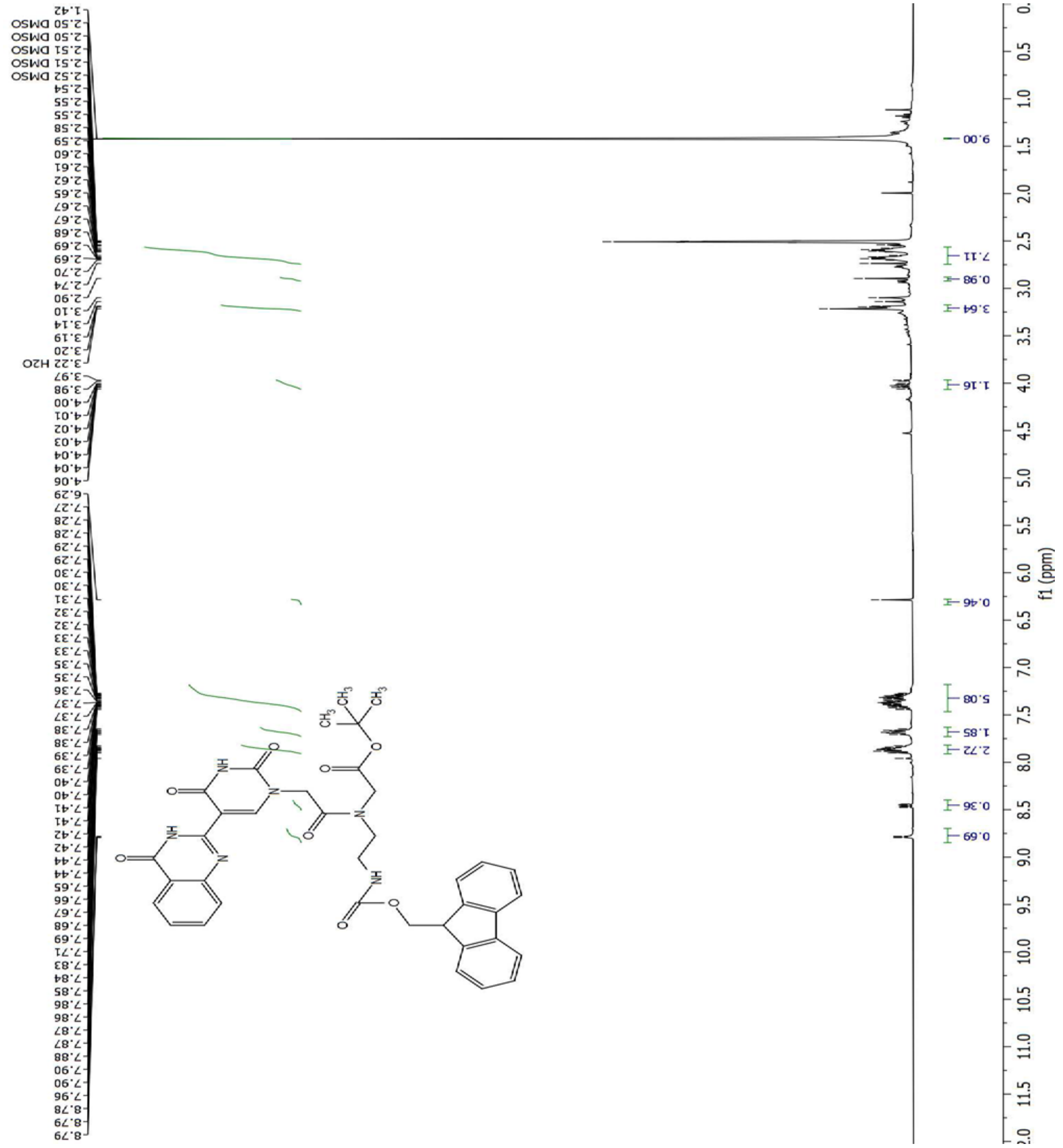

^{13}C NMR spectrum of *tert*-Butyl N-(2-((((9H-fluoren-9-yl)methoxy)carbonyl)amino)ethyl)-N-(2-(2,4-dioxo-5-(4-oxo-3,4-dihydroquinazolin-2-yl)uracil-1-yl)acetyl)glycinate (**22**)

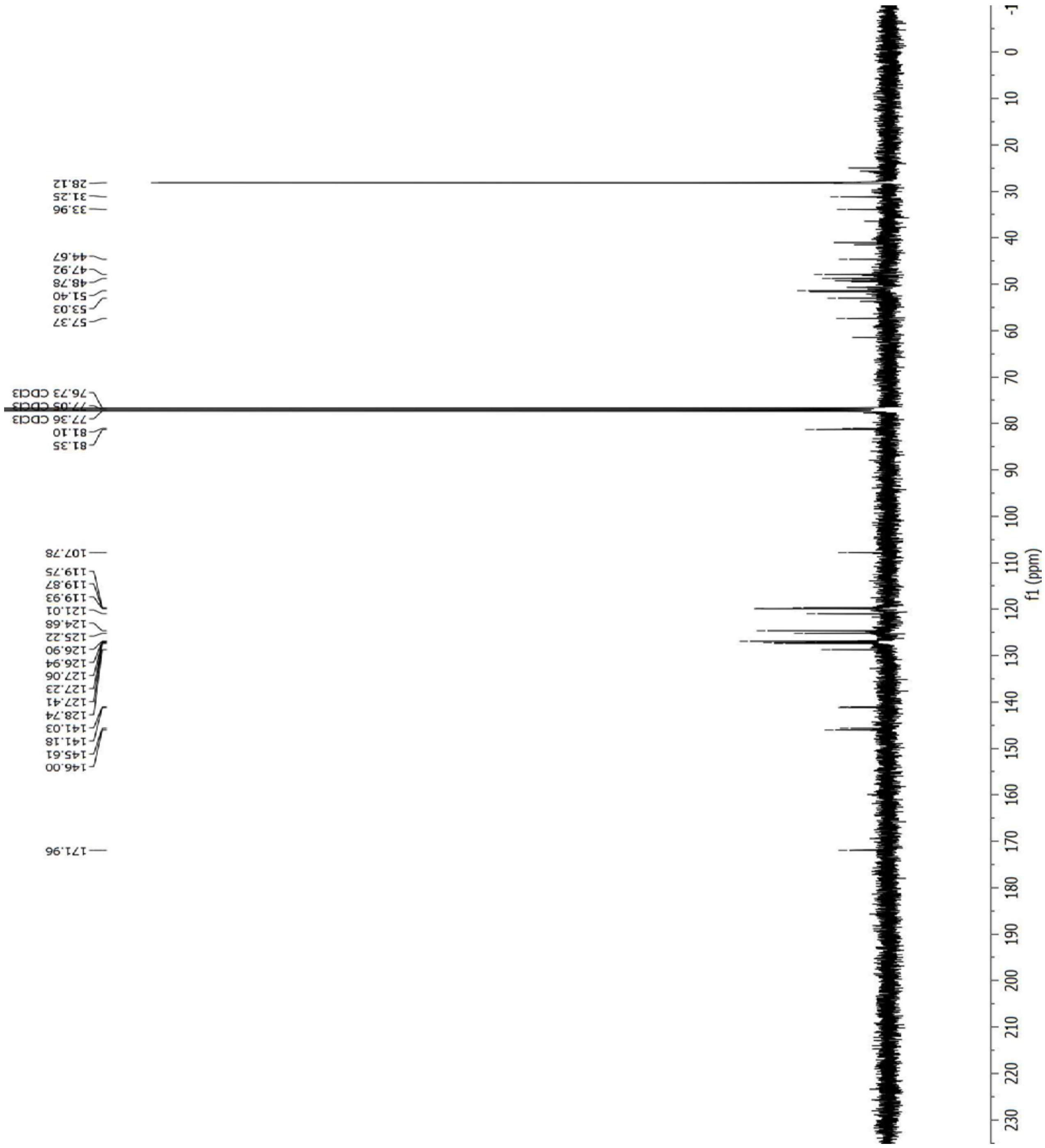

^{1}H NMR spectrum of *tert*-Butyl *N*-(2-((((9H-fluoren-9-yl)methoxy)carbonyl)amino)-ethyl)-*N*-(2-(5-(6-methoxy-4-oxo-3,4-dihydroquinazolin-2-yl)uracil-1-yl)acetyl)glycinate (**23**)

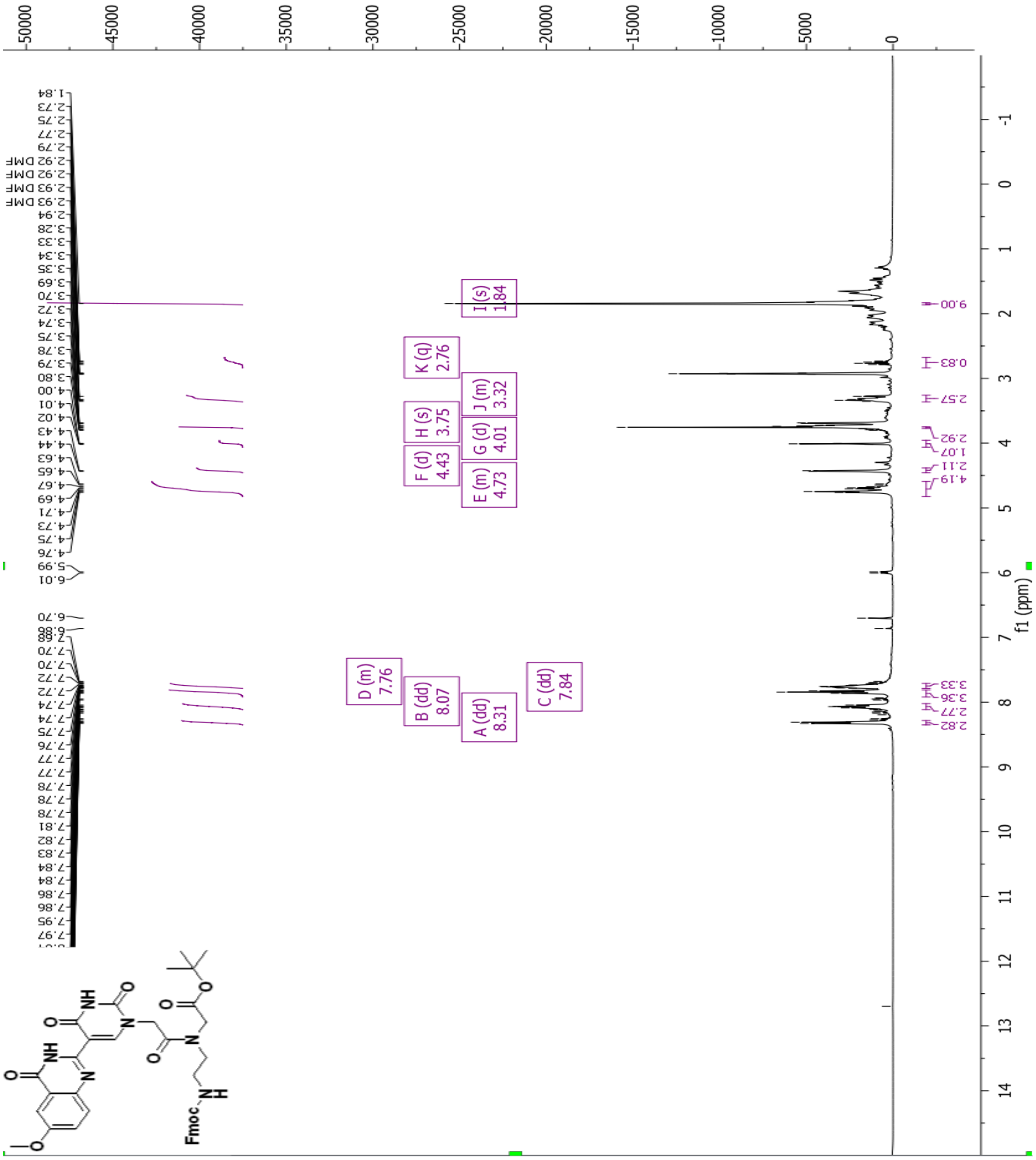

^{13}C NMR spectrum of *tert*-butyl *N*-(2-((((9H-fluoren-9-yl)methoxy)carbonyl)amino)-ethyl)-*N*-(2-(5-(6-methoxy-4-oxo-3,4-dihydroquinazolin-2-yl)uracil-1-yl)acetyl)glycinate (**23**)

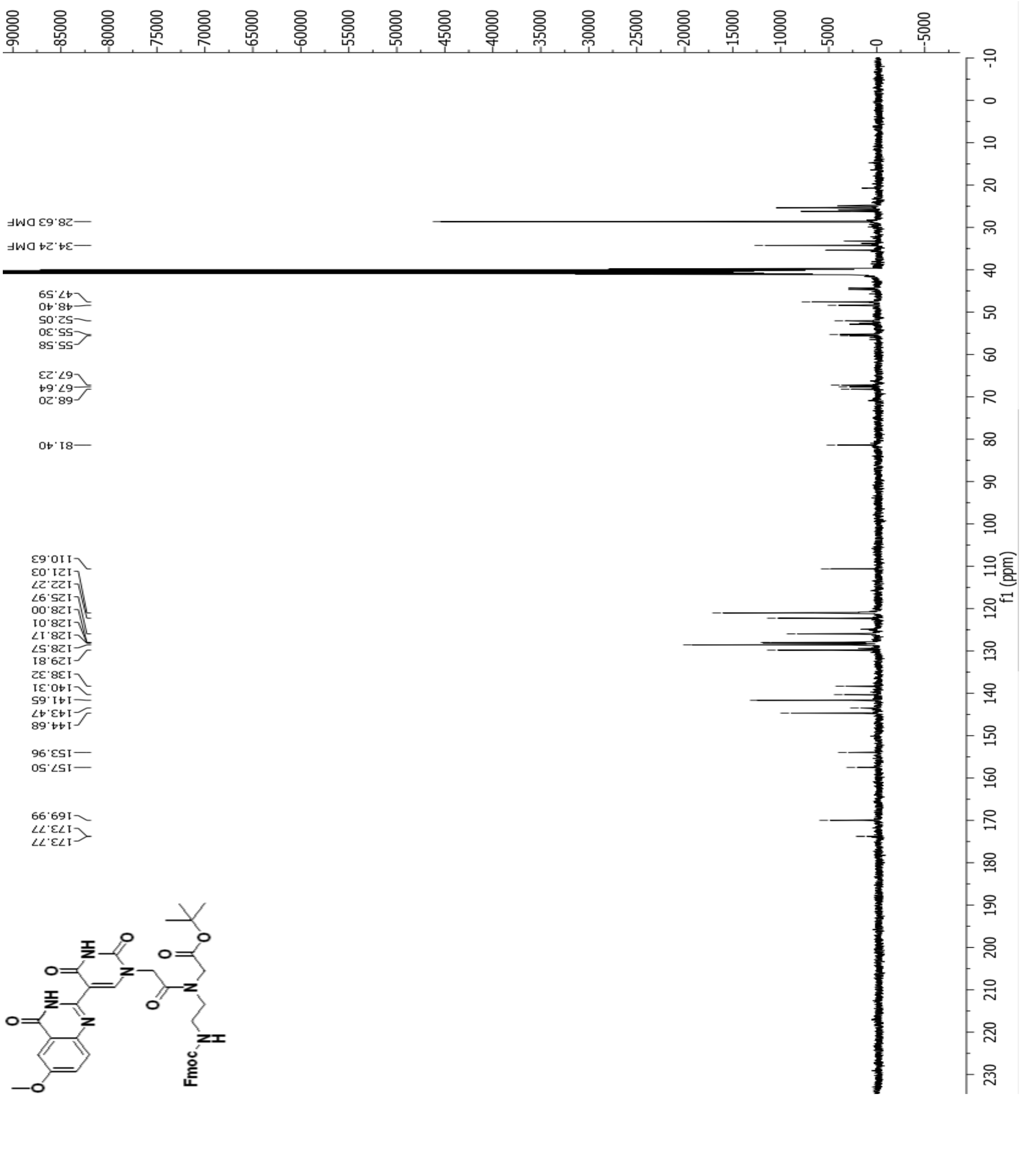

^{1}H NMR spectrum of *tert*-Butyl *N*-(2-((((9H-fluoren-9-yl)methoxy)carbonyl)amino)-ethyl)-*N*-(2-(5-(6-nitro-4-oxo-3,4-dihydroquinazolin-2-yl)uracil-1-yl)acetyl)glycinate (**24**)

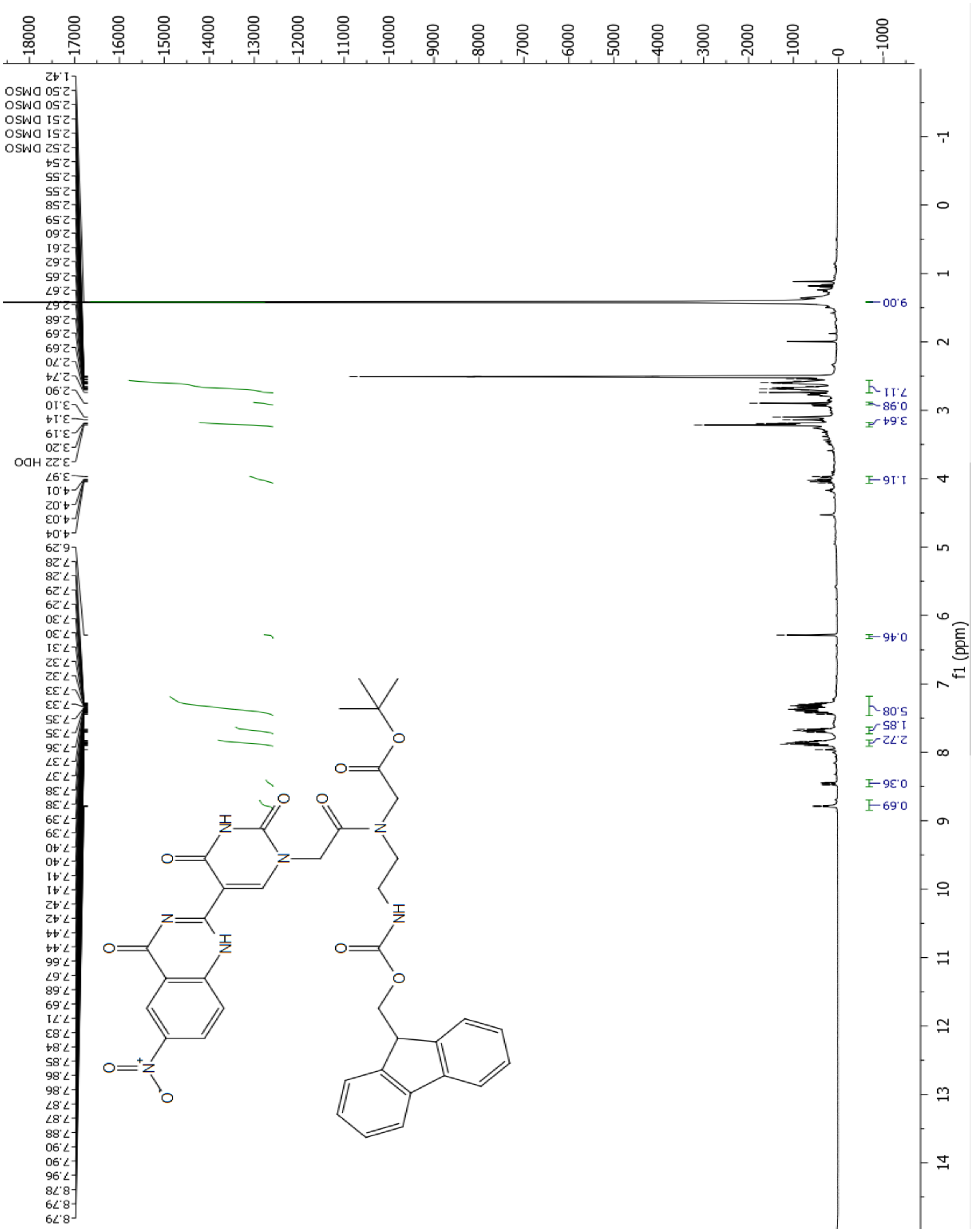

^{13}C NMR spectrum of tert-butyl N-(2-((((9H-fluoren-9-yl)methoxy)carbonyl)amino)-ethyl)-N-(2-(5-(6-nitro-4-oxo-3,4-dihydroquinazolin-2-yl)uracil-1-yl)acetyl)glycinate (**24**)

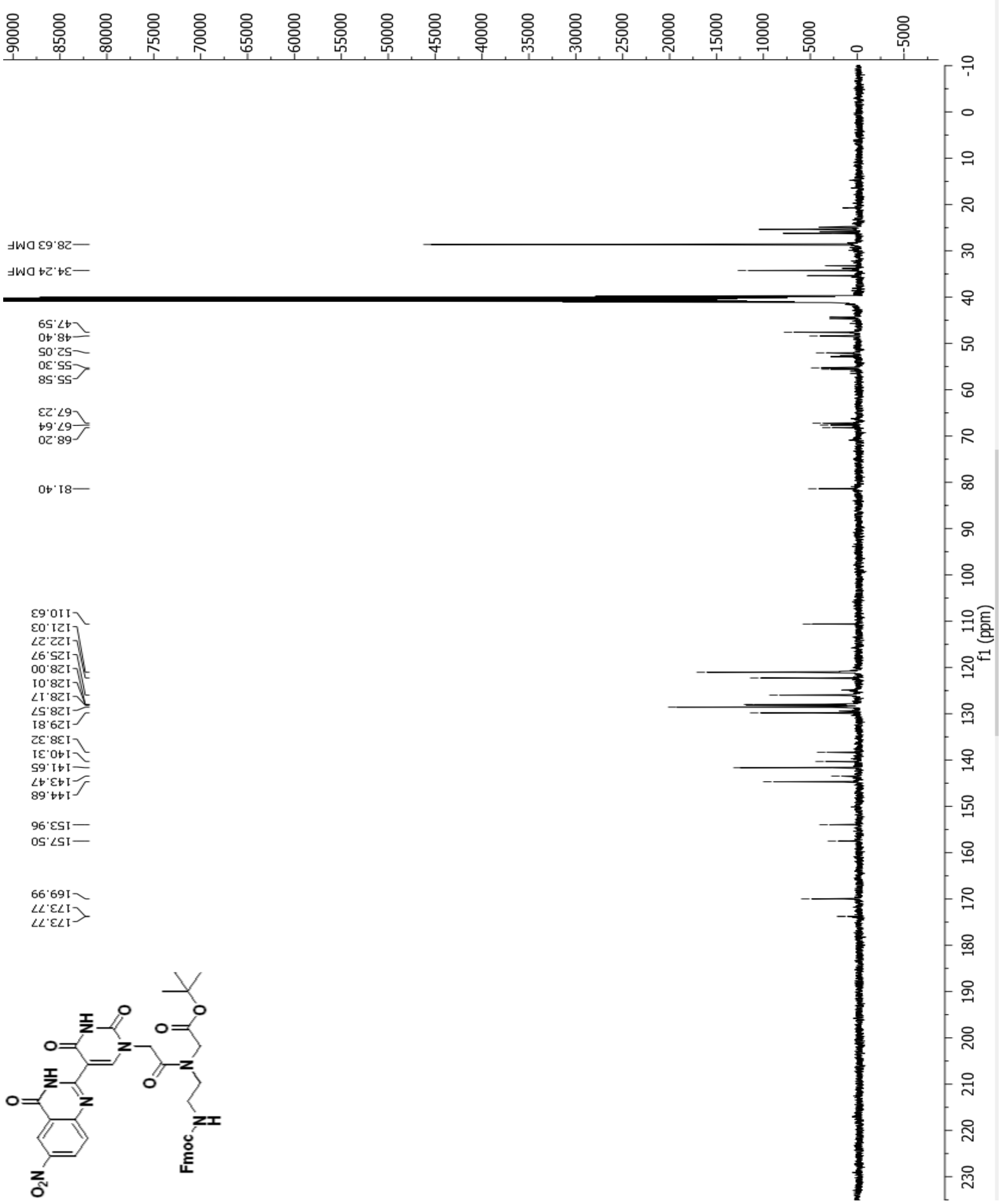

^{1}H NMR spectrum of N-(2-((((9H-fluoren-9-yl)methoxy)carbonyl)amino)ethyl)-N-(2-(2,4-dioxo-5-(4-oxo-3,4-dihydroquinazolin-2-yl)uracil-1-yl)acetyl)glycine (**25**)

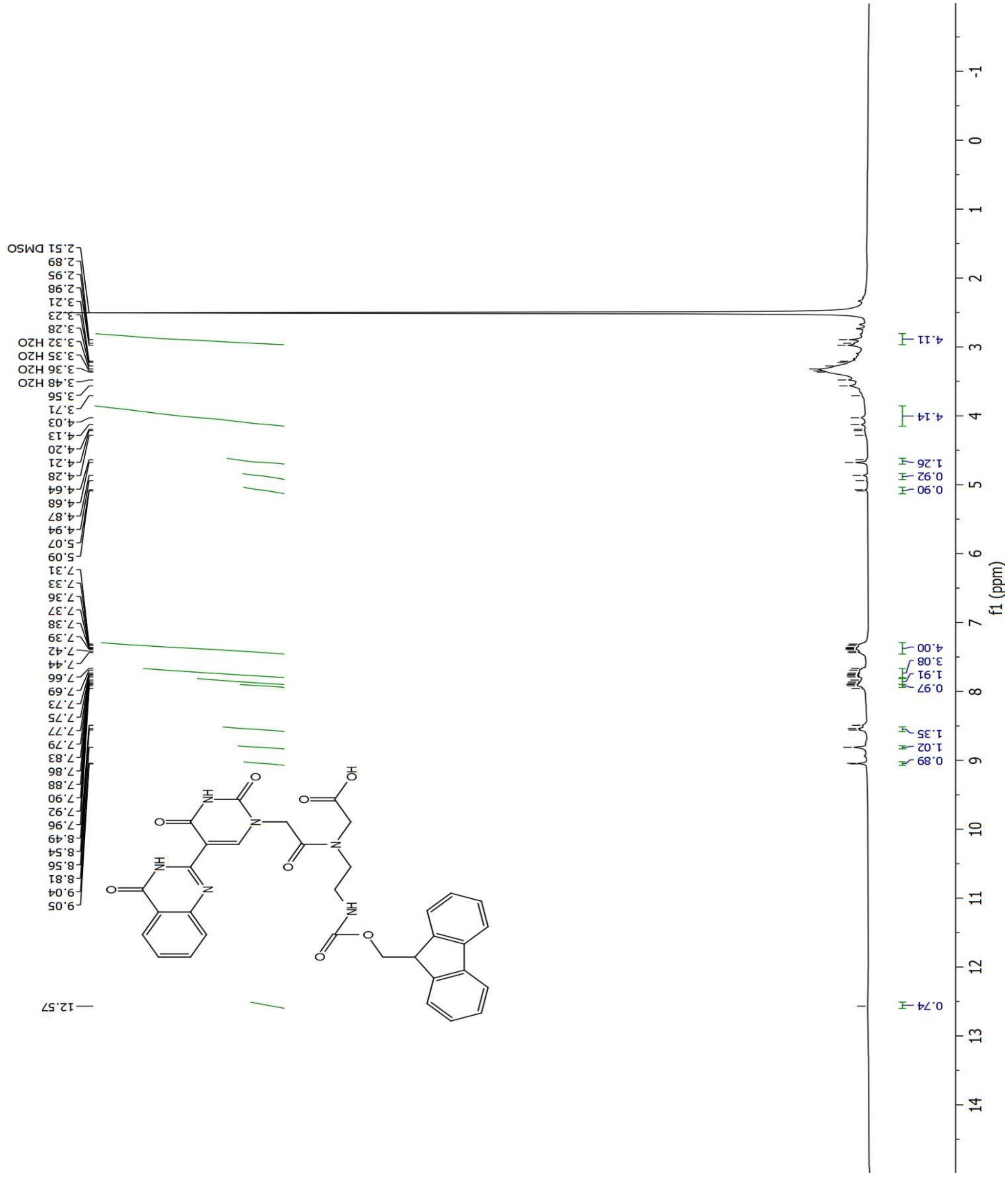

^{13}C NMR spectrum of N-(2-((((9H-fluoren-9-yl)methoxy)carbonyl)amino)ethyl)-N-(2-(2,4-dioxo-5-(4-oxo-3,4-dihydroquinazolin-2-yl)uracil-1-yl)acetyl)glycine (**25**)

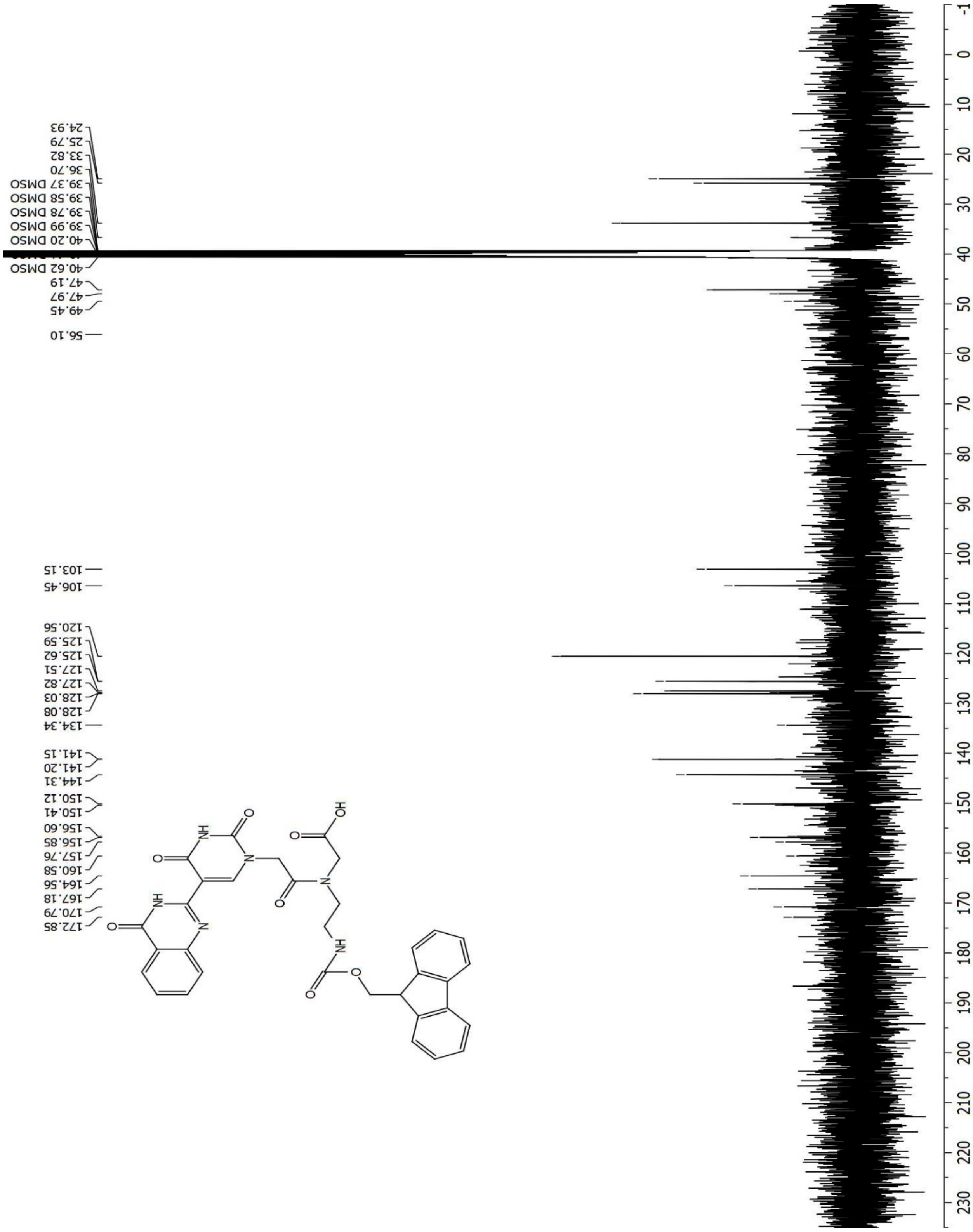

^{1}H NMR spectrum of *N*-(2-((((9H-fluoren-9-yl)methoxy)carbonyl)amino)ethyl)-*N*-(2-(5-(6-methoxy-4-oxo-3,4-dihydroquinazolin-2-yl)uracil-1-yl)acetyl)glycine (**26**)

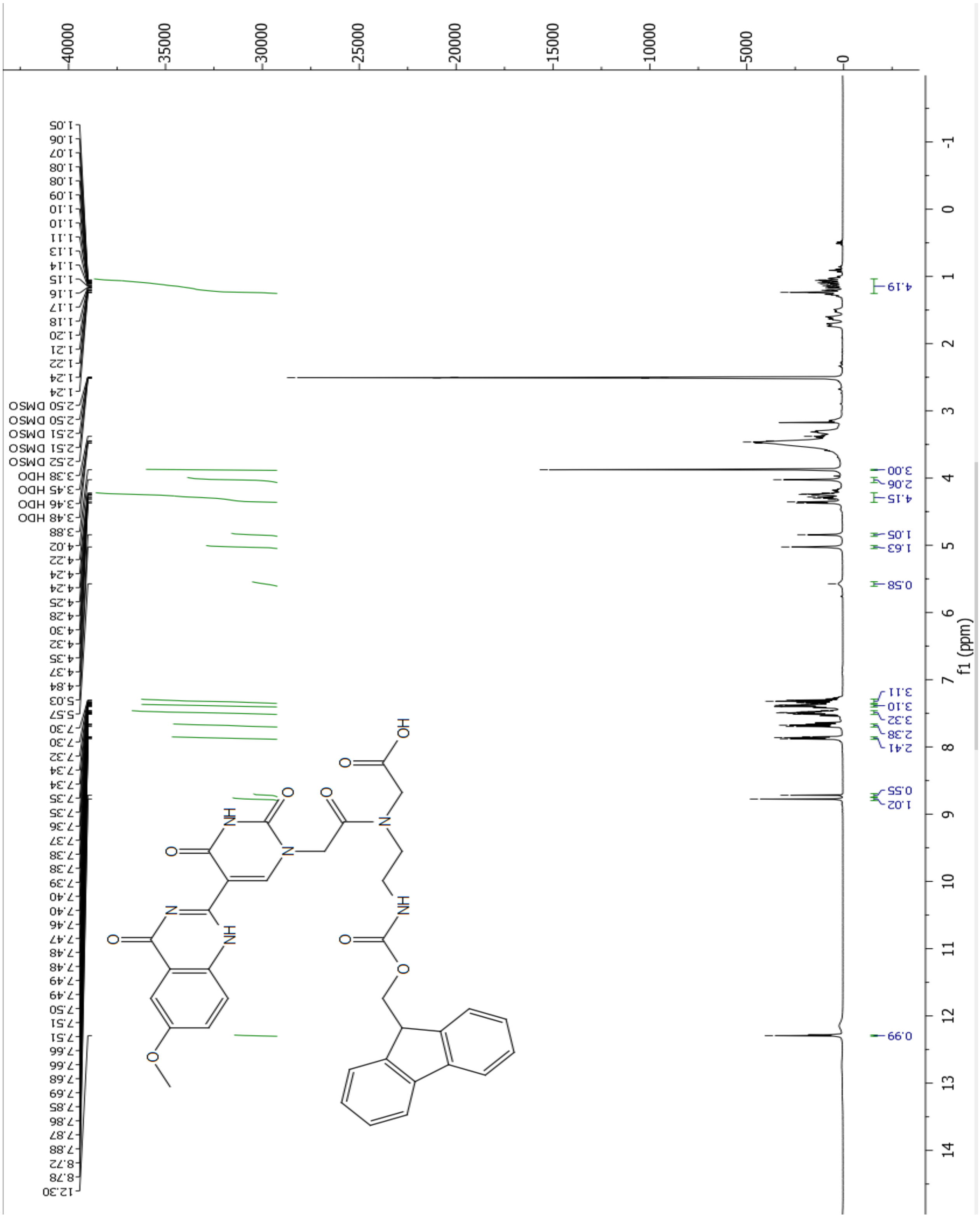

^{13}C NMR spectrum of *N*-(2-((((9H-fluoren-9-yl)methoxy)carbonyl)amino)ethyl)-*N*-(2-(5-(6-methoxy-4-oxo-3,4-dihydroquinazolin-2-yl)uracil-1-yl)acetyl)glycine (**26**)

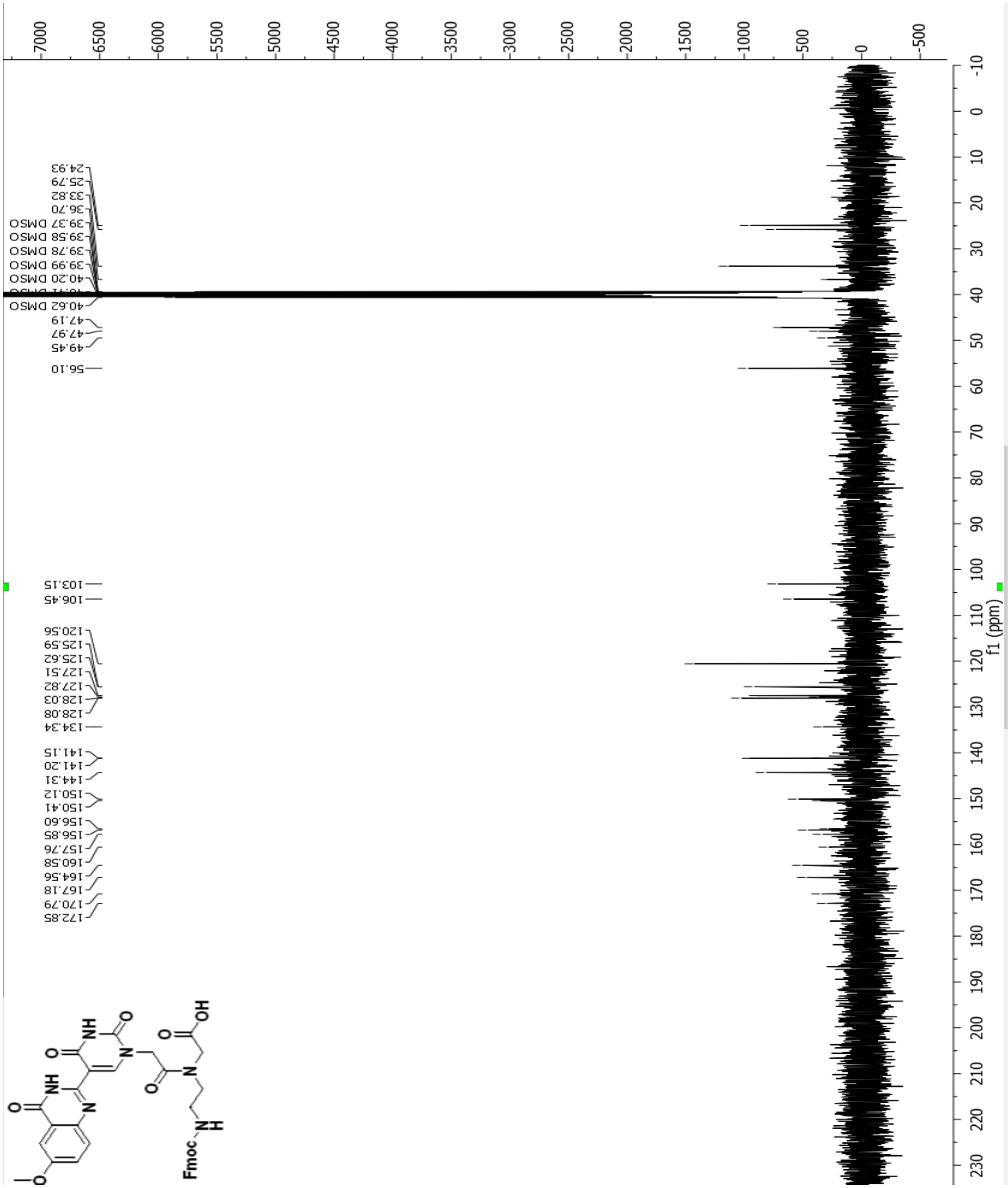

^{1}H NMR spectrum of *N*-(2-((((9H-fluoren-9-yl)methoxy)carbonyl)amino)ethyl)-*N*-(2-(5-(6-nitro-4-oxo-3,4-dihydroquinazolin-2-yl)uracil-1-yl)acetyl)glycine (**27**)

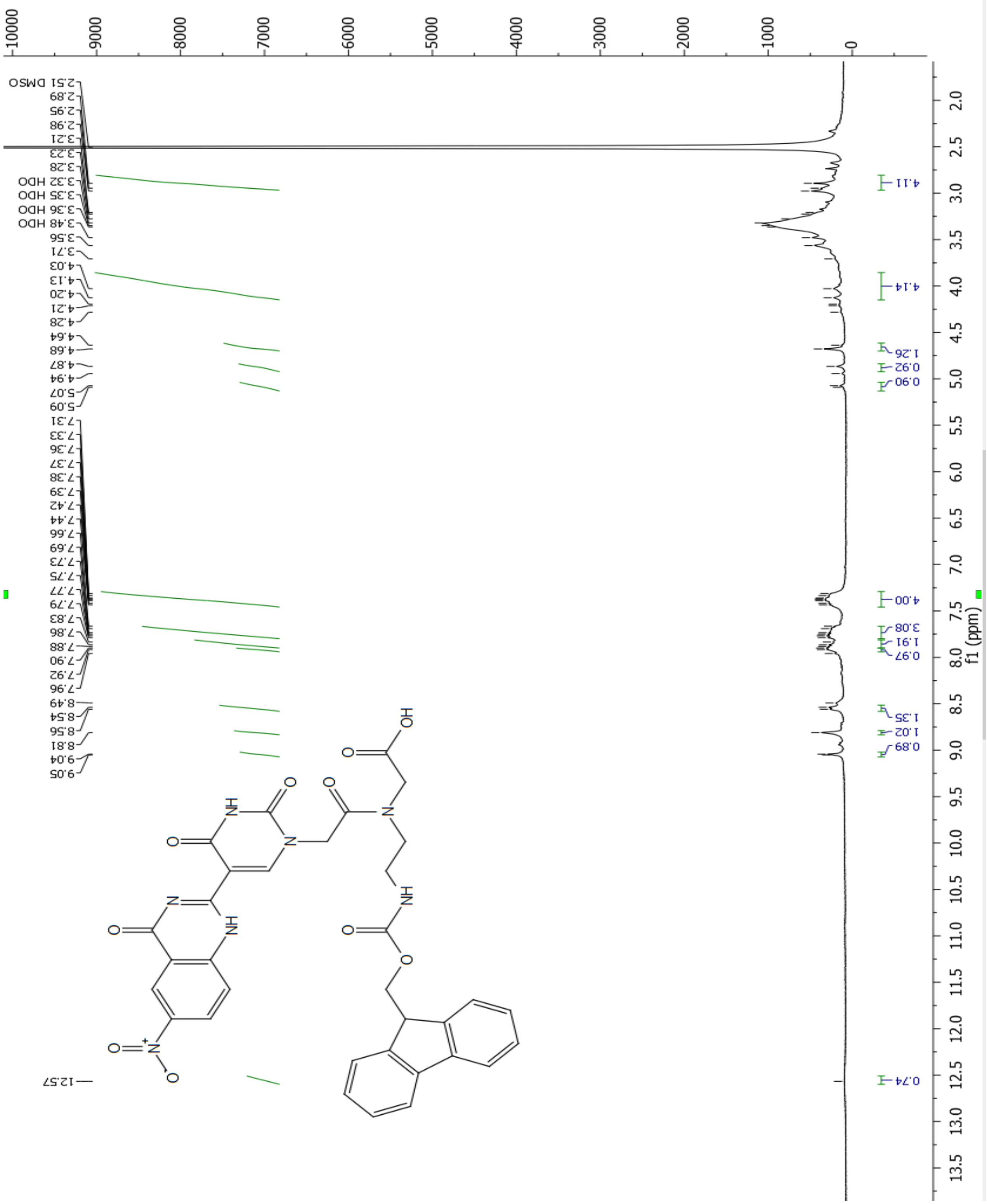

^{13}C NMR spectrum of *N*-(2-((((9H-fluoren-9-yl)methoxy)carbonyl)amino)ethyl)-*N*-(2-(5-(6-nitro-4-oxo-3,4-dihydroquinazolin-2-yl)uracil-1-yl)acetyl)glycine (**27)**

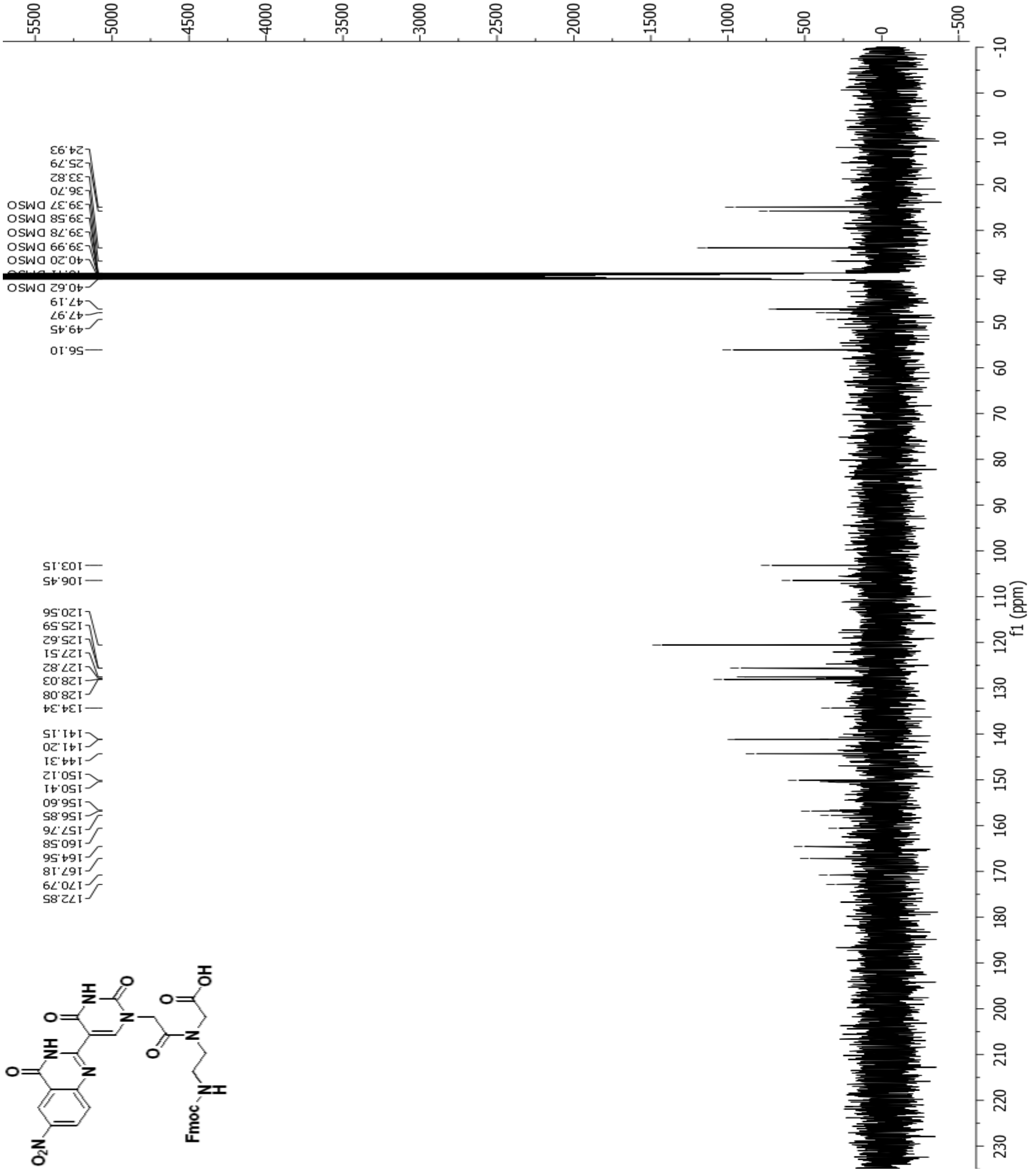

Supplement: Supplementary file 1 [file molecules-25-01995-s001.pdf]
